# Supplementary material for: Spatial Transcriptomics As Rasterized Image Tensors (STARIT) characterizes cell states with subcellular molecular heterogeneity
Source: PLoS Comput Biol. 2026 Oct 6;22(10):e1014845. doi: 10.1371/journal.pcbi.1014845 (PMC13657327; doi:10.1371/journal.pcbi.1014845)
Supplement: Supplementary file 1 [file pcbi.1014845.s021.pdf]

Point by Point Response – Overview

We sincerely thank the editor and the reviewers for their insightful and constructive feedback in helping us improve this manuscript. We have now revised the commentary to address all the points raised by the reviewers, organized herein as a point-by-point response. Throughout this point-by-point response, reviewer comments are shown in **blue**, with our responses in **green**, and changes to the manuscript in **black**.

****

Reviewer #1

Reviewer #1: The manuscript presents a novel and well-implemented framework (STARIT) for incorporating subcellular transcript distribution into spatial transcriptomics analysis. The ability to differentiate between nuclear and perinuclear transcript localization is a significant advancement over traditional count-based representations. The overall presentation is clear, and the workflow is understandable.

However, while the core idea is compelling, the manuscript would benefit from greater methodological transparency and clearer experimental organization. Specifically, stronger justification of modeling choices, clearer dataset summaries would enhance both readability and rigor.

First, a concise summary table for each dataset would be helpful. Currently, the dataset number, clustering parameters, and method performance across the simulated, noisy simulated, osmFISH, and bacterial-MERFISH datasets are described throughout the text, making it difficult for readers to compare results across conditions. A structured summary would improve clarity and transparency.

We thank the reviewer for the suggestion. We agree that key dataset characteristics, analysis settings, and performance metrics were previously distributed throughout the manuscript, making cross-dataset comparisons difficult. To improve clarity and transparency, we have added two summary tables to the Supplementary Material. Supplementary Table 1 (“Summary of datasets, analysis configurations, and clustering performance metrics”) consolidates, for every dataset and feature extraction strategy evaluated, the resulting feature matrix dimensions, whether feature weighting was applied, the full dimensionality-reduction and clustering workflow and parameters, the number of clusters identified, and ARI and mean on-diagonal Jaccard similarity where ground truth was available. Supplementary Table 3 (“Overview of datasets”) summarizes all ten datasets analyzed in this study: the seven simulated datasets, the osmFISH mouse cortex dataset, and the bacterial-MERFISH datasets (unstandardized and standardized for rotation), including a description, number of cells, number of genes/targets, and number of ground-truth cell-types or states. Together, these tables allow readers to directly compare STARIT performance and analysis configurations across all datasets without having to cross-reference the text.

Second, the choice of ResNet101 as the feature extractor needs clearer justification. ResNet101 is an older architecture trained on natural images, and it is unclear why this model was chosen over alternatives.

We thank the reviewer for the feedback. We have now evaluated multiple pretrained feature-extraction models— ResNet18, ResNet50, ResNet101, ResNet152, CTransPath, and UNI2-h—across our simulated and real imSRT datasets (Supplementary Table 1). Using a left-right polarized simulated dataset designed to test sensitivity to orientation (Supplementary Figure 1, Methods 4.4.2), ResNet101 correctly resolved all four simulated groups, while UNI2-h and CTransPath—self-supervised learning frameworks that heavily rely on data augmentations in their pretraining that enforce rotational invariance through augmentation on histopathology data—recovered only three clusters, with reduced separation of the two oppositely-polarized groups (Results 2.1, lines 154-182; Supplementary Figure 1B-C). While the other ResNet architectures (ResNet18, 50, and 152) performed comparably to ResNet101(Supplementary Table 1), we selected ResNet101 as the default feature extractor for STARIT due to its deeper architecture and representational capacity. This added depth enables the extraction of richer, finer-grained visual features compared to ResNets with less convolutional layers, a property that becomes increasingly vital as datasets scale in cells and genes when thinking about STARIT downstream analysis. For users operating under limited computational resources, ResNet50 is a lighter-weight alternative. Nonetheless, we have expanded both the Results (lines 154-181) and Discussion (lines 514-519) to clarify that there is no required or "best" feature extractor, and that STARIT is compatible with any downstream computer vision model for feature extraction. We emphasize that feature-extractor selection should be guided by the biological question and expected sources of spatial variation (e.g., whether orientation is itself biologically meaningful, as in apical–basal polarity, or a technical nuisance). The revised text is provided below for the reviewer's convenience, as well as an accompanying figure showcasing results (Supplementary Figure 1):

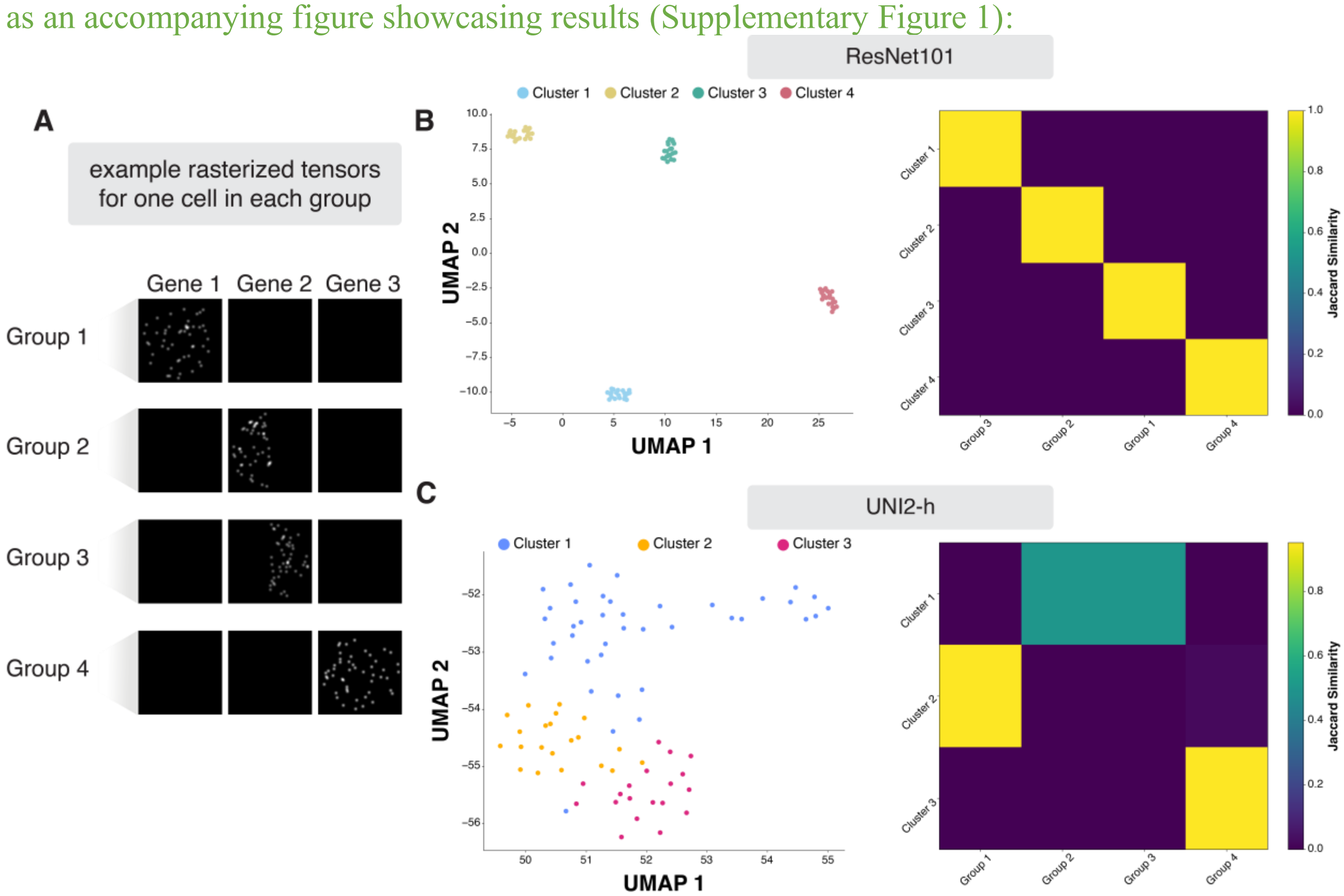

Supplementary Figure 1. Comparison of STARIT-derived ResNet101 features versus STARIT-derived UNI2-h features for left-right polarized localization simulation imSRT dataset. (A)

Representative STARIT rasterized image tensors for one cell from each of the four simulated groups. (B) Analysis results of STARIT tensors using ResNet101 feature extraction. Left: UMAP visualization based on STARIT output, embedding colored by Louvain cluster assignment. Right: Jaccard similarity heatmap between ground-truth and STARIT-based Louvain clusters. (C) Analysis results STARIT tensors using UNI2-h feature extraction. Left: UMAP visualization based on STARIT output, embedding colored by Louvain cluster assignment. Right: Jaccard similarity heatmap between ground-truth and STARIT-based Louvain clusters.

(Results 2.1, Lines 151-177) "Of note, while we used ResNet101, a feature extractor originally developed for natural images, for this analysis, STARIT is compatible with any downstream computer vision model for feature extraction. Although STARIT does not require the use of any specific feature extractor, feature extractor choice can influence which forms of spatial heterogeneity are emphasized. To demonstrate this, we generated an additional simulated dataset in which Groups 1 and 4 again uniquely expressed Gene 1 and Gene 3, respectively, while Groups 2 and 3 uniquely expressed Gene 2, now with left- versus right-polarized intracellular organization (Supplementary Figure 1A, Methods). Applying STARIT with ResNet101, we resolved four clusters corresponding to the four simulated groups, indicating that cell states defined by left- versus right-polarized transcript localization can be resolved (Supplementary Figure 1B). However, when we applied STARIT with UNI2-h, a feature extractor developed from a histopathology foundation model based on a vision transformer (ViT) encoder (ViT-H/14), to the same dataset, the analysis recovered three clusters, with reduced separation of the left- and right-polarized Groups 2 and 3 cells (Supplementary Figure 1C) [9]. Similar behavior was observed for CTransPath, another feature extractor for pathology images. In contrast, additional evaluated ResNet architectures including ResNet18, ResNet50, and ResNet152 more closely matched ResNet101 performance (Supplementary Table 1). This behavior may be expected because CTransPath, for example, is trained using frameworks that incorporate data augmentations such as random rotations and flips in learned visual representations [10]. For UNI2-h, detailed augmentation follows DINOv2 and other self supervised ViT frameworks, which typically apply geometric transforms [9]. Because standard ViTs are not rotation-equivariant by design, we hypothesize that any rotational invariance in UNI2-h and related pathology foundation models arise from its pretraining and augmentations rather than the architecture itself. This result highlights that feature extractor choice may determine whether left- versus right-polarized spatial patterns are preserved or diminished. Such sensitivity may be desirable when left- versus right-polarized spatial patterns is biologically meaningful, in the case of apical-basal organization, but undesirable when orientation reflects technical or arbitrary differences in cell positioning, for example. Thus, feature extractor selection should be guided by the biological question and expected sources of spatial variation in the dataset."

(Discussion, Lines 514-519) "The choice of feature extractor is another important consideration. We primarily used ResNet101 as the feature extractor though we also evaluated alternative architectures, including smaller and larger ResNet models as well as domain-specific or foundation-model feature extractors such as CTransPath and UNI2-h (Supplementary Table 1). These comparisons suggest that feature extractor choice can influence downstream clustering and should be selected based on the biological question, data, and expected sources of variation."

Reviewer #2

Reviewer #2: In this manuscript, the authors investigated the use of a computer vision model, ResNet101, to help identify cell-states based on imaging-based spatially resolved transcriptomics (imSRT). They identify a crucial limitation of the current single-cell gene count based methods which fail to take subcellular heterogeneity into account. They show excellent results on synthetic data but when applying their approach to real imSRT data, the results are more mixed and it is not clear they provide an improvement over existing methods. This work contributes to the field by presenting original research in a valuable area of study. However, revisions are necessary before publication.

Major comments:

1. Based on results, it is not clear to that the approach provides any practical advantage on real world data over existing gene-count approaches. When comparing Supplementary Fig. 5C with Fig. 2H, it is not clear which result is better. The authors present quantitative results for their approach (ARI and on-diagonal Jaccard similarities) on lines 204-205. However, they do not present the same for the existing approaches. I suggest adding the direct quantitative comparison of the approaches. If other comparable non-gene-count approaches exist they should also be included in this quantitative comparison if possible.

We thank the reviewer for the feedback and address this in two ways. First, we have now included the osmFISH data similar quantitative results for the gene-count clustering osmFISH (ARI, mean on-diagonal Jaccard similarity) results to aid in comparison of (now) Supplementary Fig. 8D with Fig. 2H, consistent with the quantitative results presented throughout the paper. Both weighted STARIT and gene-count clustering approaches achieve comparable overall correspondence with the annotated anatomical regions (Supplementary Figure 7) and annotated cell-types (Supplementary Figure 8), with ARIs of 0.299 and 0.342, and mean on-diagonal Jaccard similarities of 0.272 and 0.295 respectively. And, notably, both approaches independently split the annotated mature oligodendrocyte population into the same two subclusters driven by the same five differentially expressed genes (*Anln, Ctps, Gfap, Itpr2, Plp1*; Bonferroni-corrected $p < 0.001$) (Results 2.3, Figure 2D-H, Supplementary Figure 8A-D, lines 293-326). This demonstrates that STARIT captures comparable transcriptional heterogeneity to conventional gene-count analysis on this dataset. The relevant text is provided below for the reviewer’s convenience, as well as accompanying figures showcasing results:

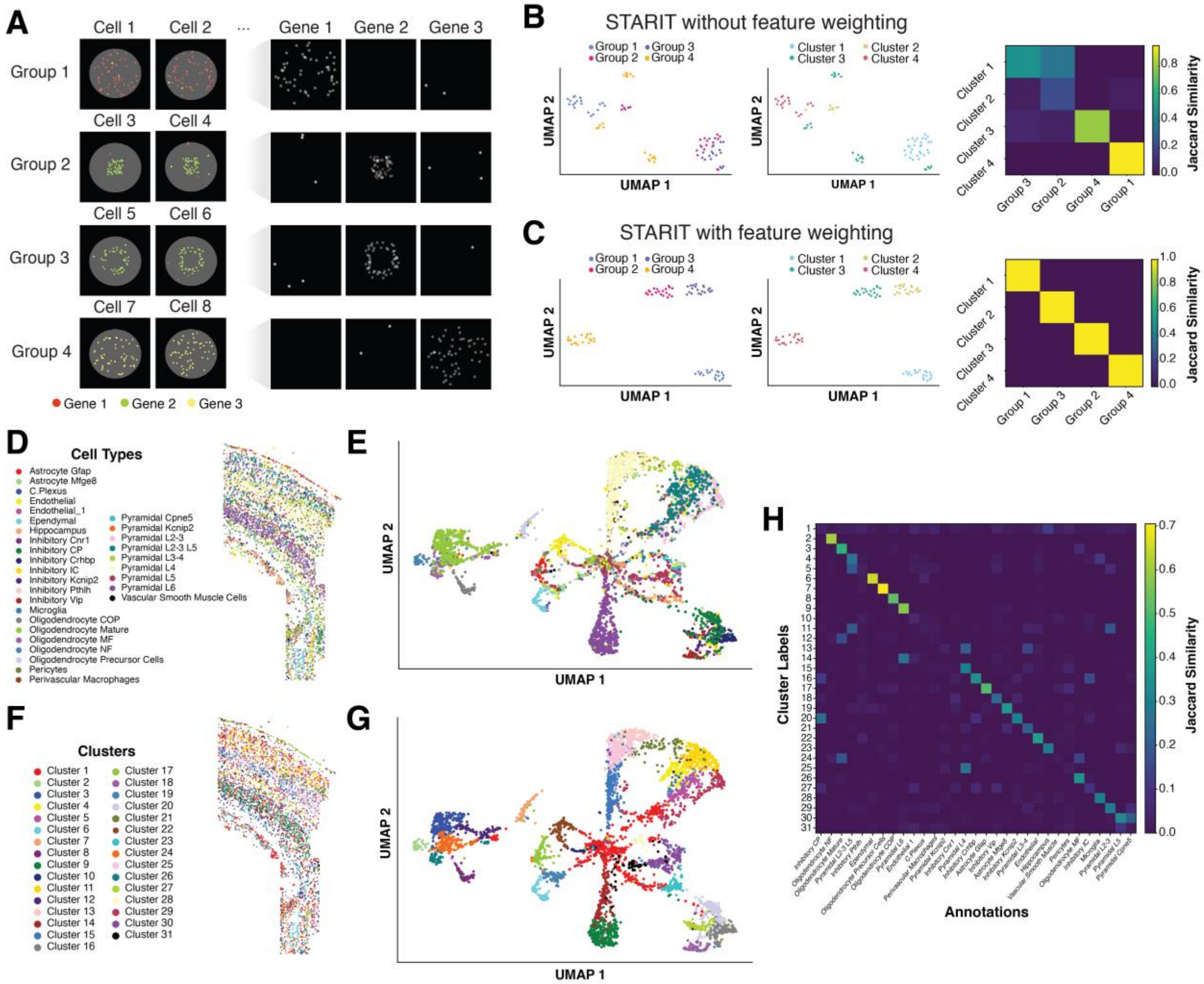

Figure 2. STARIT with feature weighting and application to osmFISH mouse cortex dataset. (A) Simulated imSRT data comprises four groups of cells (Groups 1–4) that exhibit distinct subcellular molecular heterogeneity. Left: Example simulated cells from each group. Groups are defined by distinct transcript expression and localization patterns across three genes (Gene 1, red; Gene 2, green; Gene 3, yellow). Groups 2 and 3 differ by nuclear versus perinuclear localization of Gene 2. All groups contain simulated noise across all genes. (B) STARIT analysis of simulated imSRT data with introduced noise, without feature weighting. Left: UMAP visualization of cells colored by ground-truth group annotation. Middle: UMAP visualization of cells colored by Louvain cluster assignment. Right: Jaccard similarity heatmap comparing ground-truth group annotations to Louvain clusters. (C) STARIT analysis of simulated imSRT data with introduced noise, with gene expression–based feature weighting. Left: UMAP visualization of cells colored by ground-truth group annotation. Middle: UMAP visualization of cells colored by Louvain cluster assignment. Right: Jaccard similarity heatmap comparing ground-truth group annotations to Louvain clusters. (D) Ground-truth cell-type annotations of osmFISH mouse somatosensory cortex dataset (Codeluppi *et al.*, 2018) in tissue space. (E) UMAP visualization of osmFISH mouse somatosensory cortex ground-truth cell-types on ResNet101-extracted STARIT features. (F) STARIT-derived Louvain clustering of osmFISH mouse somatosensory cortex dataset in tissue space. (G) UMAP visualization of STARIT-derived Louvain clusters of osmFISH mouse somatosensory cortex from

ResNet101-extracted STARIT features. (H) Jaccard similarity heatmap comparing ground-truth osmFISH mouse somatosensory cortex annotations to STARIT-derived Louvain clusters.

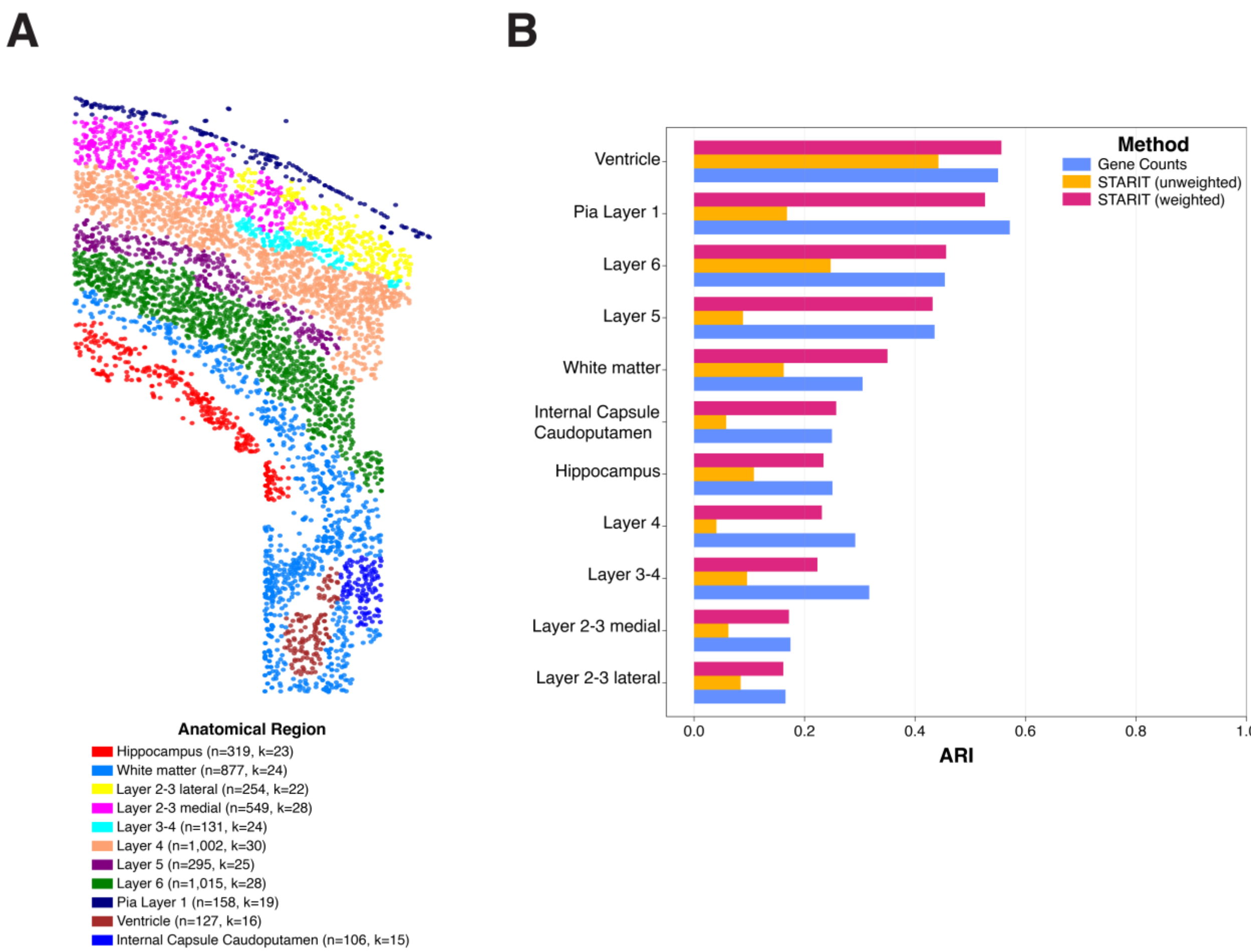

Supplementary Figure 7. Clustering agreements of osmFISH ground-truth cell types by anatomical region. (A) Visualization of the 4,833 profiled cells in the osmFISH mouse somatosensory cortex dataset reported by Codeluppi *et al.* (2018) in tissue space, colored by the 11 annotated anatomical regions. Legend indicates the number of cells (*n*) and ground-truth annotated cell types (*k*) within each region. (B) Grouped horizontal bar chart evaluating Adjusted Rand Index (ARI) (x-axis) scores comparing ground-truth cell-type annotations with Louvain clusters derived from scaled gene counts (blue), unweighted STARIT image features (orange), and weighted STARIT image features (pink) across anatomical regions (y-axis). Regions are ordered by weighted STARIT ARI score (descending).

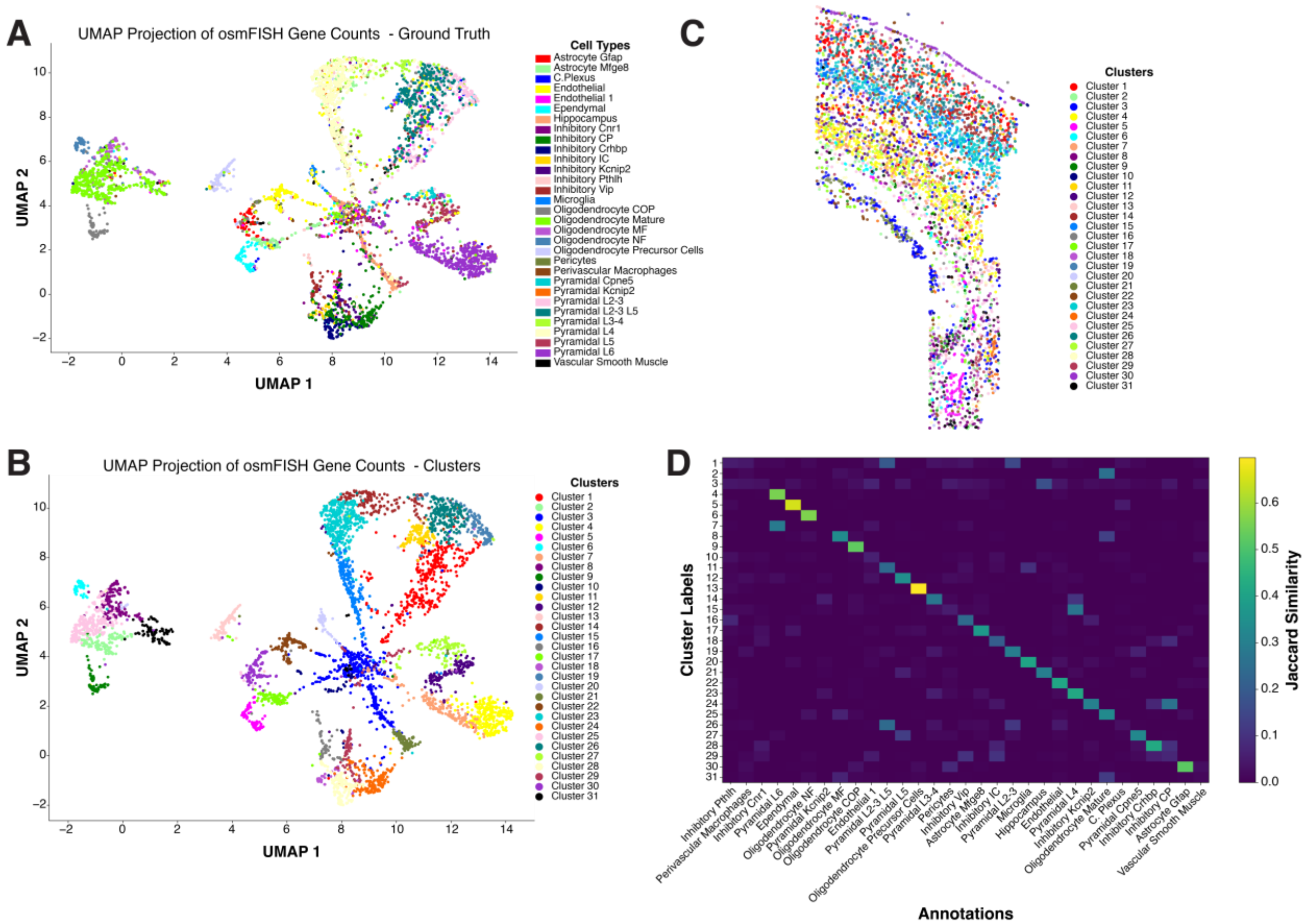

Supplementary Figure 8. Gene count–based clustering of osmFISH mouse cortex data. (A) UMAP embedding of PCs from the gene count matrix of the osmFISH mouse cortex dataset, colored by the 31 previously annotated cell types from *Codeluppi et al.* (2018). (B) UMAP embedding of the same gene count–derived features colored by 31 Louvain clusters from STARIT-derived ResNet101 image features followed by agglomerative clustering. (C) Gene counts clustering of osmFISH mouse somatosensory cortex dataset in tissue space. (D) Jaccard similarity matrix comparing gene count–derived Louvain clusters (rows) to annotated cell types (columns).

(Results 2.3, Lines 293-306) "Having demonstrated that feature weighting can be an effective strategy to handle potential minor segmentation errors and/or noisy gene detection, we incorporate such feature weighting into our analysis of the osmFISH data. With feature weighting, we observe improved correspondence between our identified clusters and the original cell-type annotations, as reflected in the similar patterns in tissue and UMAP space as well as improved ARI with original cell types (score: 0.299) and higher on-diagonal Jaccard similarities (mean: 0.272) (Figs. 2D-H). Furthermore, feature weighting led to higher clustering agreement with the annotated anatomical regions, yielding an improved ARI compared to unweighted STARIT features (Supplementary Figure 7). We do observe some new clusters unique to our STARIT analysis. For example, mature oligodendrocyte cells from the original osmFISH annotation are now split into clusters 3 and 24. As shown with our simulated data, such clusters may reflect distinct subcellular heterogeneity uniquely distinguishable through our STARIT analysis. We again used the Wilcoxon-rank sum test on the features between these two clusters to identify significantly differential features

(Bonferroni-corrected p-value < 0.001) corresponding to the *Anln, Ctps, Gfap, Itpr2,* and *Plp1* genes' STARIT representations."

(Results 2.3, Lines 306-326) "To evaluate whether our newly identified clusters are solely due to STARIT or could be attributable to inherent instability in gene count clustering analysis, we performed normalization, PCA dimensionality reduction, followed by Louvain graph-based and agglomerative clustering to identify 31 clusters from re-clustering on the original gene counts matrix. We were able to observe similar clustering results for the gene counts re-clustering as from our STARIT analysis, reflected in the similar patterns in tissue and UMAP space (Supplementary Figure 7, Supplementary Figure 8), with a comparable ARI score of 0.342 and on-diagonal Jaccard similarity of 0.295 (Supplementary Figure 8D). These clustering results also included a splitting of mature oligodendrocyte cells (Supplementary Figure 8D). Consistent with this, when we used the Wilcoxon-rank sum test on the gene counts between the cells in the mature oligodendrocyte subclusters (cluster 3 and 24) from STARIT, we were able to identify the same genes (*Anln, Ctps, Gfap, Itpr2, Plp1*) as significantly differentially expressed (Bonferroni-corrected p-value < 0.001). These results suggest that STARIT identified new clusters that split annotated mature oligodendrocytes, potentially reflecting distinct cell states. However, re-clustering of gene counts could produce similar results, potentially indicating how subcellular localization changes often accompany magnitude changes. Nonetheless, STARIT captures comparable aspects of transcriptional heterogeneity as conventional gene count analysis. Furthermore, analysis of STARIT-derived image features identified genes driving cluster differences comparable to differential expression with gene count clustering analysis."

Second, we now have a new analysis with bacterial-MERFISH data that better demonstrates STARIT's advantage on real world imSRT data. Our bacterial-MERFISH analysis (Results 2.4, Figure 3) demonstrates a case where STARIT identifies subpopulations showcasing molecular heterogeneity that conventional gene-count clustering could not have found. After standardizing bacterial orientation and applying STARIT with UNI2-h, we identify 4 clusters of *E. coli* (Figure 3A) that are not explained by fliC-fliX expression magnitude (Figure 3B, E) and are not attributable to whole-cell rotation (Supplementary Figure 12), each cluster exhibiting distinct significant extracted features (Supplementary Figure 13). Using supervised interpretable metrics (Methods 4.15), we show, for example, that Clusters 2 and 3 have equivalent cell size and transcript abundance yet differ significantly in total intensity, reflecting a more diffuse versus punctate fliC-fliX spatial distribution (Results 2.4, lines 392-400; Figure 3C-F, Supplementary Figure 14). It is a concrete example of STARIT recovering molecular heterogeneity that a conventional gene-count approach would miss entirely, addressing the reviewer's concern more directly than the osmFISH comparison alone. The relevant text is provided below for the reviewer's convenience as well as accompanying figures showcasing these results:

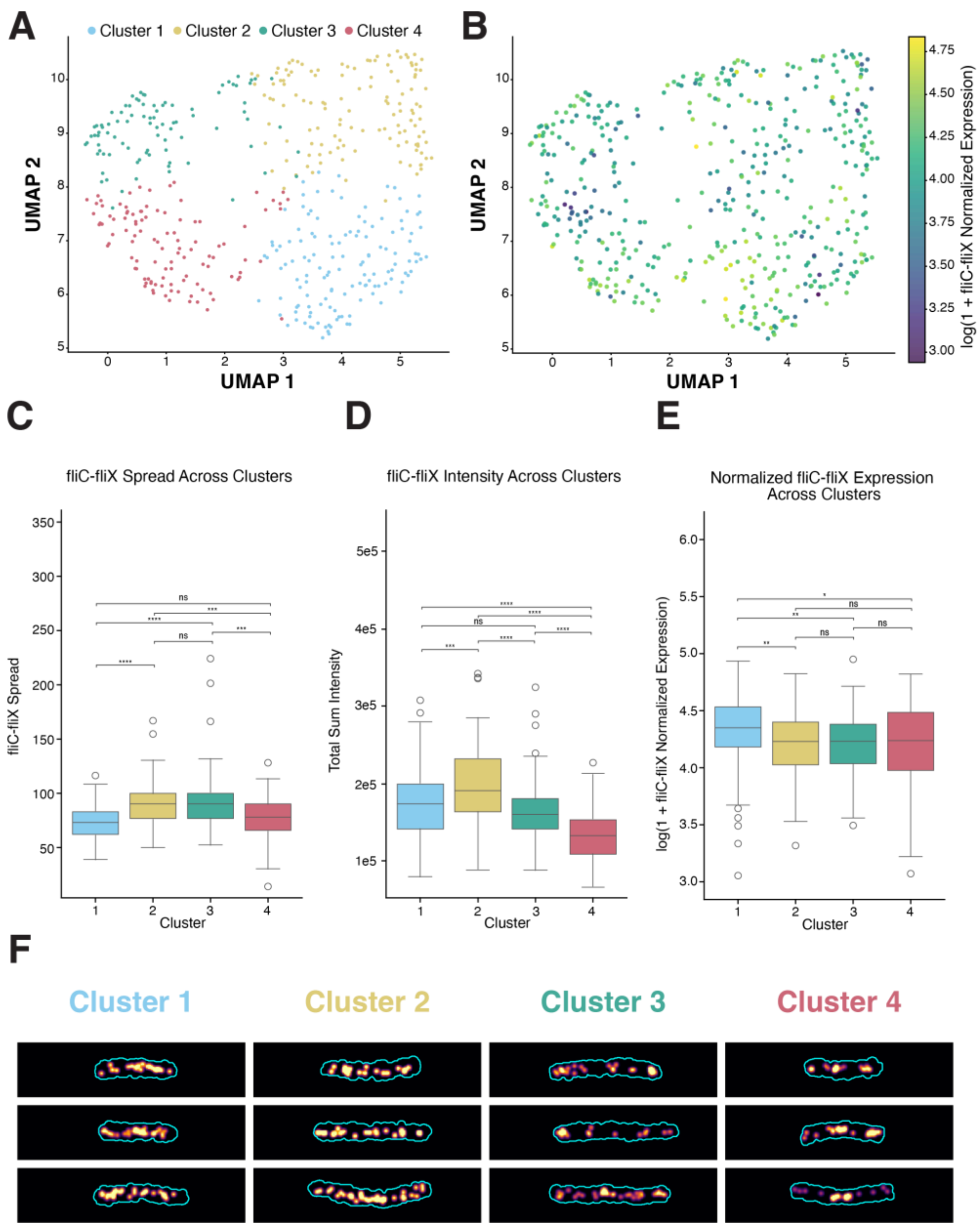

Supplementary Figure 3. STARIT-derived ResNet101 features for simulated cut-volume molecule count localization datasets. (A-D) Representative STARIT rasterized image tensors for one cell from each simulated group at (A) 100%, (B) 50%, (C) 25%, and (D) 12.5% retained cell volume. Within each panel, columns correspond to Gene 1, Gene 2, and Gene 3, and rows correspond to the four simulated groups. (E-G) Clustering analyses for simulations with (E) 50%, (F) 25%, and (G) 12.5% retained cell volume. (E) Left: UMAP visualization based on ground-truth, with embedding colored by group. Middle: UMAP visualization based on STARIT output, with embedding colored by Louvain cluster assignment. Right: Jaccard similarity heatmap between ground-truth and STARIT-based Louvain clusters. (F) Left: UMAP visualization based on ground-truth, with embedding colored by group. Middle: UMAP visualization based on STARIT output, with embedding colored by Louvain cluster assignment. Right: Jaccard similarity heatmap between ground-truth and STARIT-based Louvain clusters. (G) Left: UMAP visualization based on ground-truth, with embedding colored by group. Middle: UMAP visualization

based on STARIT output, with embedding colored by Louvain cluster assignment. Right: Jaccard similarity heatmap between ground-truth and STARIT-based Louvain clusters.

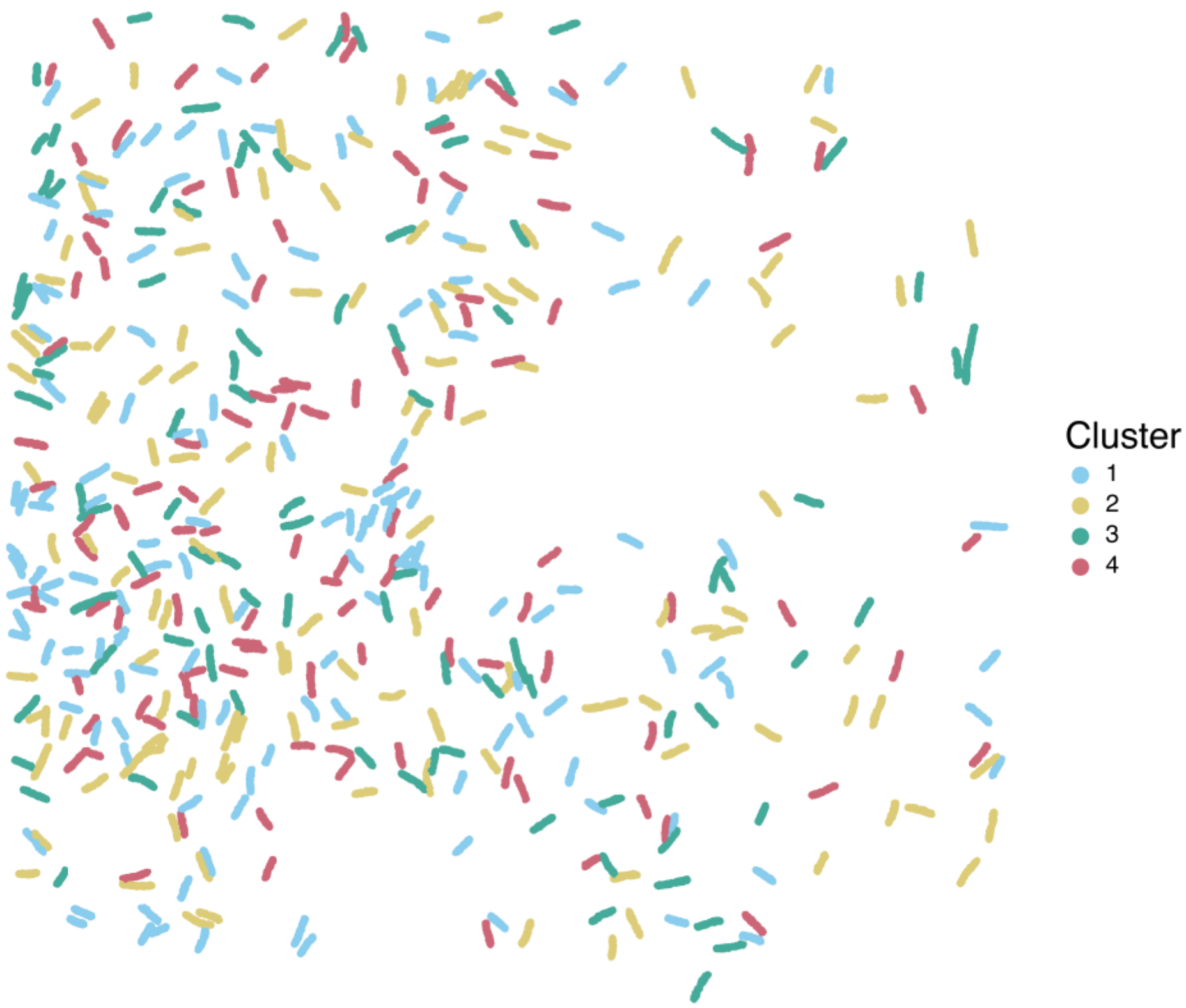

Supplementary Figure 12. Physical-space visualization of standardized STARIT-identified clusters in Bacterial-MERFISH *E. coli* data. Shown are the spatial positions of 463 *E. coli* cells assayed by Bacterial-MERFISH after 1000-fold volumetric expansion, plotted in their original physical coordinates and colored by the four Louvain clusters identified from STARIT-derived UNI2-h image features.

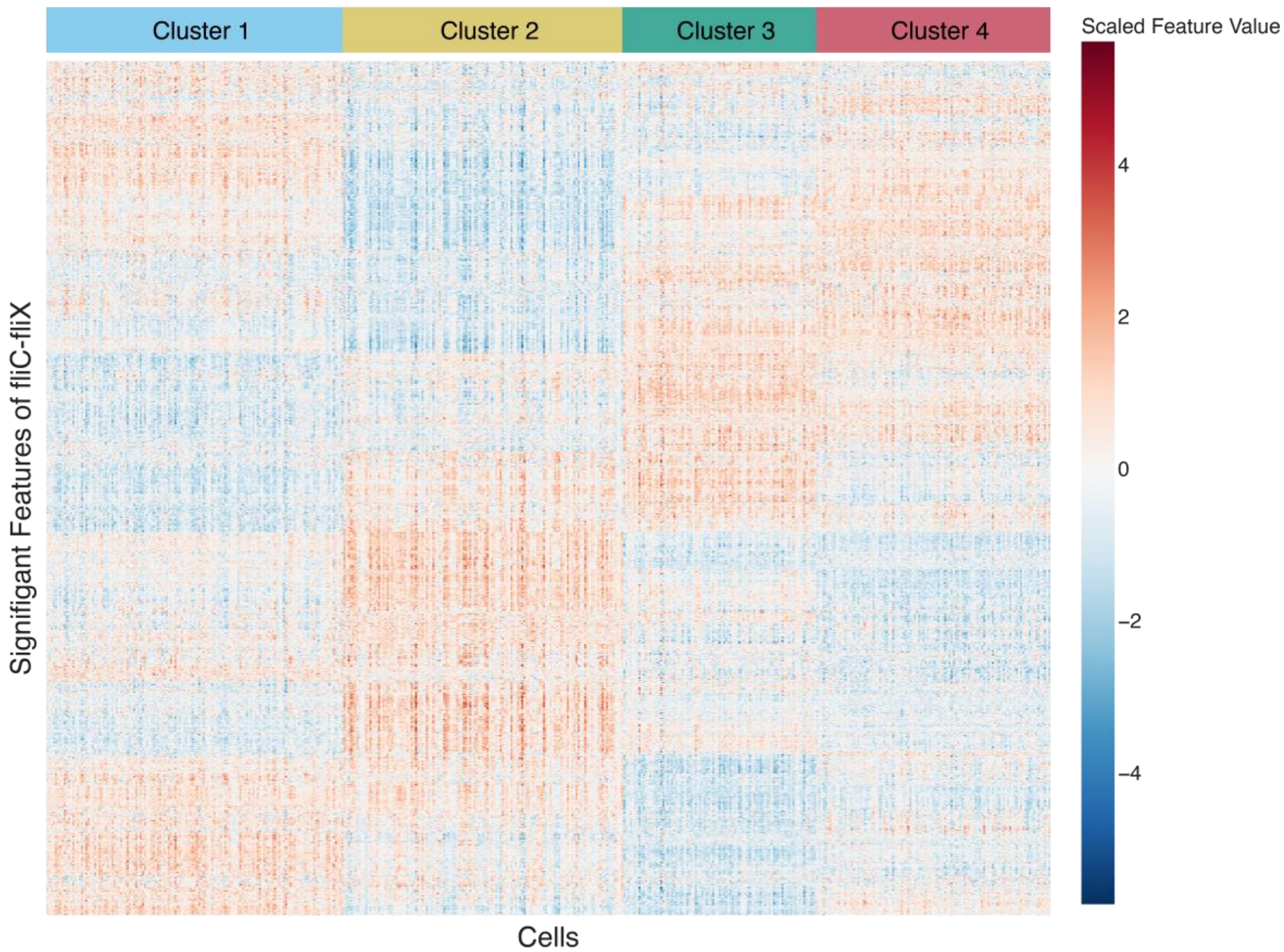

Supplementary Figure 13. Heatmap of significant extracted UNI2-h image features for STARIT-identified clusters in standardized Bacterial-MERFISH *E. coli* data. Heatmap of scaled significant (p-value < 0.001) STARIT features for fliC-fliX, shown across the identified clusters. Rows denote extracted features, and columns denote cells.

(Results 2.4, Lines 392-400) “In contrast, Cluster 2 and 3 neither significantly differ in their normalized fliC-fliX expression magnitude, spread, cell length, nor total operon count (Figure 3C, E; Supplementary Figure 14A-B). Yet, they differ significantly in fliC-fliX total intensity, with Cluster 2 cells exhibiting higher total intensities than Cluster 3 cells (Figure 3B). We can thus interpret Cluster 2 cells as having a more diffuse distribution of fliC-fliX transcripts throughout the bacterium and Cluster 3 cells as having a more punctated distribution of fliC-fliX transcripts (Figure 3F). Again, these difference in subcellular spatial organization would be missed by conventional gene count clustering and highlights the ability of STARIT to resolve cells with distinct spatial heterogeneity.”

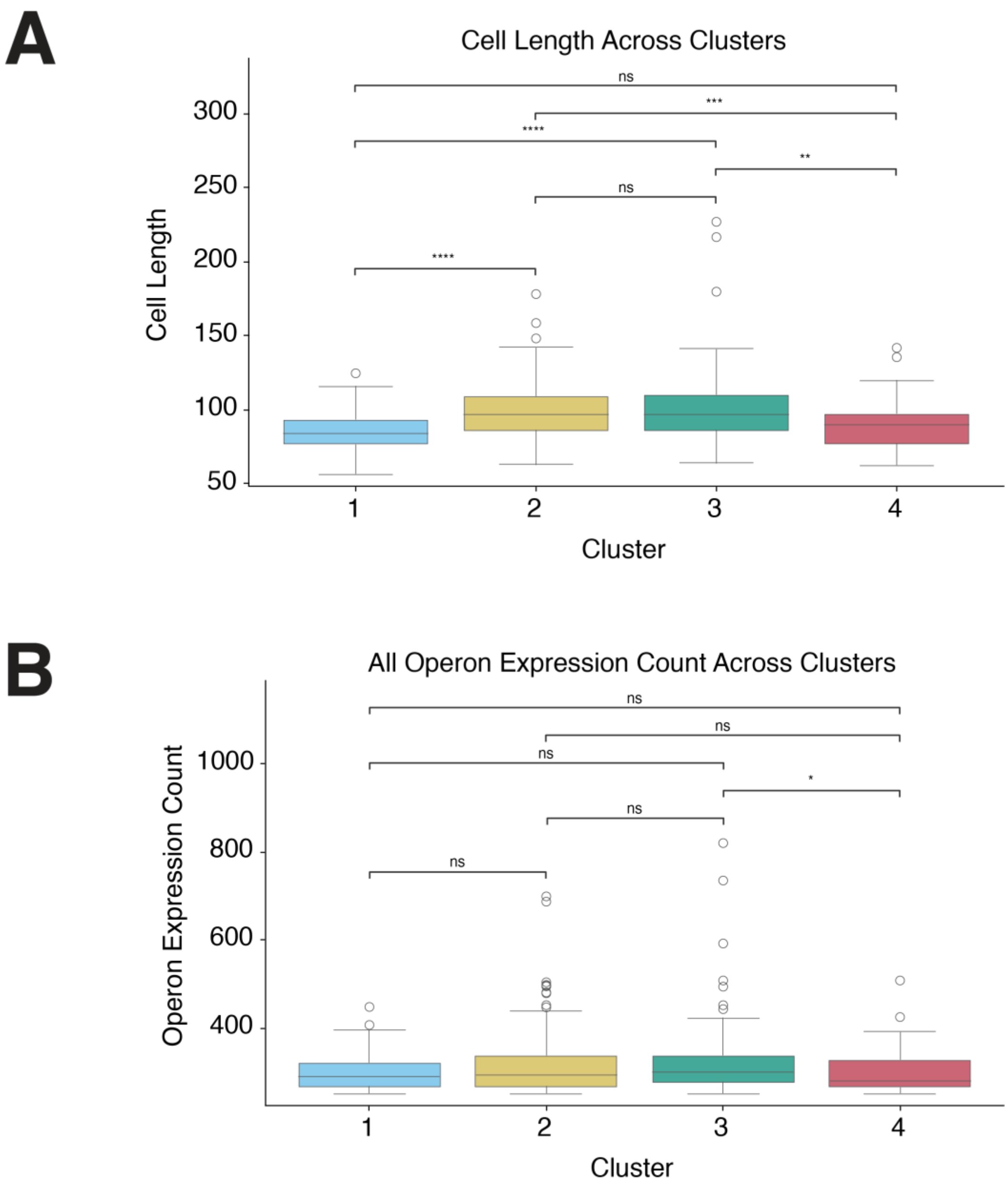

Supplementary Figure 14. Distributions of selected expression and morphology metrics among STARIT-identified bacterial clusters. Additional boxplots showing selected supervised metrics for the four clusters identified from standardized bacterial-MERFISH *E. coli* data. (A) Distribution of total cell length in X across bacterial clusters. Boxplots display median intensity (line), interquartile range (box), and outliers, with colors denoting cluster. Statistical significance determined by Mann-Whitney U test (Bonferroni corrected). (B) Distribution of all operon expression count across bacterial clusters. Boxplots display median intensity (line), interquartile range (box), and outliers, with colors denoting cluster. Statistical significance determined by Mann-Whitney U test (Bonferroni corrected).

We have not identified an existing unsupervised non-gene-count computational method for identifying subcellular heterogeneity directly comparable to STARIT for a quantitative

benchmark. We discuss graph- and point-cloud-based alternatives currently used for other tasks like cell-type annotation, cell segmentation, and subcellular pattern classification in the Discussion (lines 566-575).

(Discussion, Lines 566-575) “Although STARIT currently uses rasterized image representations, rasterization is only one strategy for encoding subcellular transcript organization. Alternative representations may directly model transcript coordinates without converting them into pixels through rasterization. Graph-based approaches have already been used in spatial omics to model spatial organization at the tissue or cellular-neighborhood level [37–39]. Likewise, such graph-based representations of transcript locations within cells has been demonstrated to improve cell type annotation [40] and cell segmentation [41]. Alternative point-cloud-based representations have also been developed to classify subcellular RNA localization patterns [42]. Future work comparing these representations can help determine which best captures biologically relevant information for specific analytical objectives.”

2. I am not convinced that ResNet101 is well suited for this task for a variety of reasons.
a. CNNs are known to struggle with sparse data which seems to be the case here (Fig. 1C).
b. With a learned feature vector for each gene, the resulting feature vector is very large (>67,000 here) and uninterpretable. It is unclear how much of the information in this large feature vector is meaningful rather than artifacts of the rasterization or other factors.
c. CNNs are not invariant to orientation or scale.
d. ResNet101 is very large and perhaps a smaller version like ResNet18 (or even simpler CNNs) might provide similar results at reduced computational cost and potentially less overfitting.

We thank the reviewer for these thoughtful points, which we have addressed both empirically and in the Discussion. Regarding (a)-(d), we systematically compared ResNet18, ResNet50, ResNet101, ResNet152, CTransPath, and UNI2-h across our simulated and real imSRT datasets (Supplementary Table 1). ResNet18 and the other smaller/larger ResNet variants performed comparably to ResNet101 in our simulations, supporting the reviewer’s intuition that a smaller model can suffice. However, in certain simulations, smaller ResNet variants like ResNet18 faced performance loss compared to larger ResNet variants, no longer reliably preserving the underlying spatial patterns (simulated dataset #4, 7; Supplementary Table 1). We recommend ResNet50 as an option for users with limited computational resources (Results 2.1, lines 151-177; Discussion, lines 514-519). We agree that the >67,000 dimensional feature vector is not fully interpretable. We discuss this limitation explicitly (Discussion, lines 496-513) and address it empirically for the bacterial-MERFISH data by deriving a set of interpretable, supervised morphological and intensity metrics (spatial spread, total intensity, normalized expression, cell length, etc.; Methods 4.15, Supplementary Table 2; Figure 3C-F; Supplementary Figure 14) that we correlate with the raw extracted image features (max Spearman values = 0.61-0.64) and use to characterize what distinguishes each cluster (Results 2.4, lines 376-409; Figure 3C-F). The revised text is provided below for the reviewer’s convenience, along with accompanying figures showcasing results:

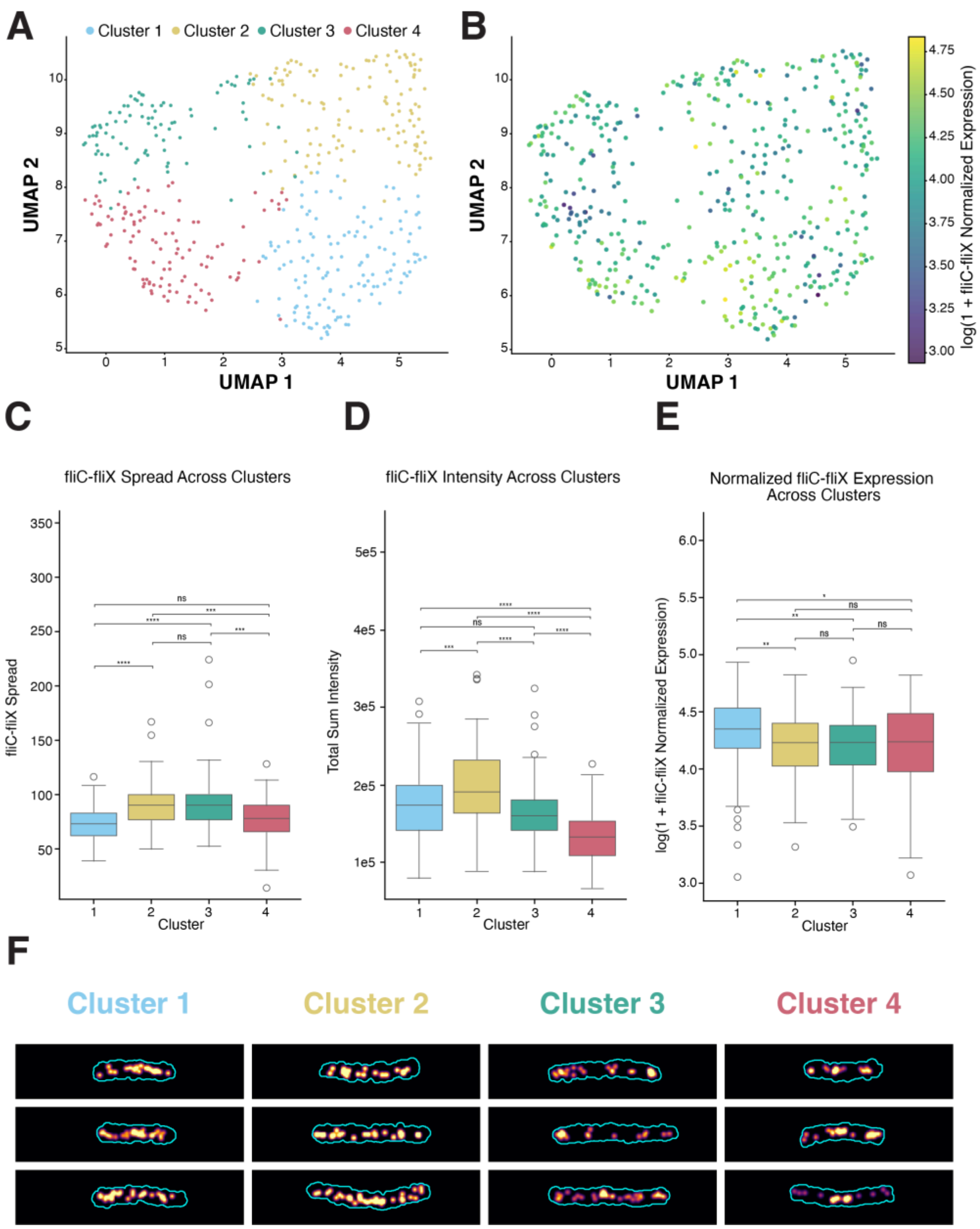

Figure 3. STARIT performance on standardized bacterial-MERFISH 1000X data. (A) Uniform Manifold Approximation and Projection (UMAP) of STARIT-derived UNI2-h features from rotationally standardized 463 *E. coli* cells based fliC-fliX operon localization, imaged with bacterial-MERFISH at 1000X volumetric expansion. (B) UMAP embedding of cells colored by log-transformed fliC-fliX gene expression levels. (C) Distribution of variation in normalized fliC-fliX gene expression across bacterial clusters. Expression levels normalized to total molecular counts per 1000 and log-transformed. Boxplots display median intensity (line), interquartile range (box), and outliers, with colors denoting cluster. Statistical significance determined by Mann-Whitney U test (Bonferroni corrected). (D) Distribution of spatial spread in X of fliC-fliX across bacterial clusters. Boxplots display median intensity (line), interquartile range (box), and outliers, with colors denoting cluster. Statistical significance determined by Mann-Whitney U test (Bonferroni corrected). (E) Distribution of total fliC-fliX signal across bacterial clusters. Boxplots display median intensity (line), interquartile range (box), and outliers, with colors

denoting cluster. Statistical significance determined by Mann-Whitney U test (Bonferroni corrected). (F) Select examples of rasterized fliC-fliX transcript distributions for cells in clusters 1-4.

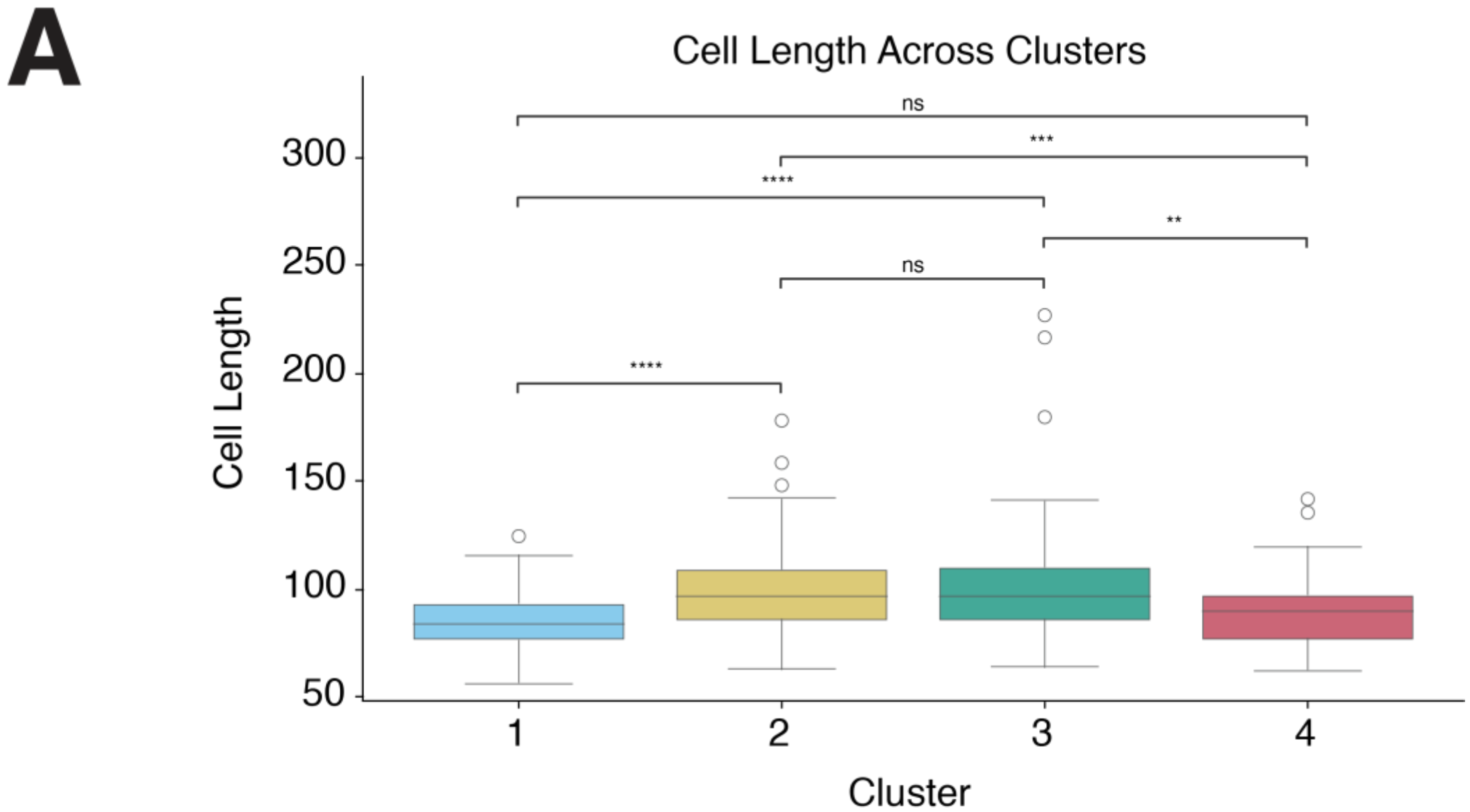

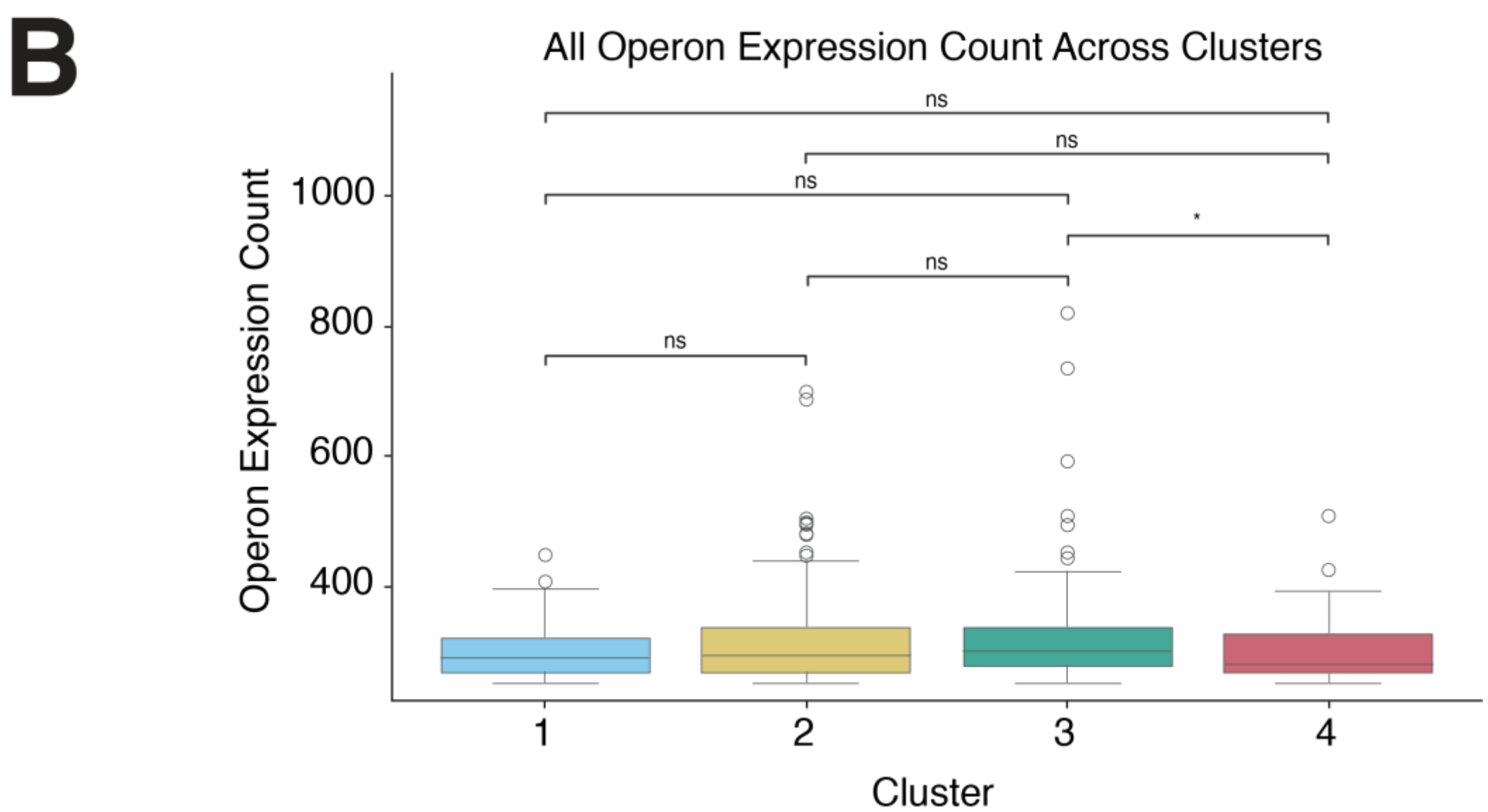

Supplementary Figure 14. Distributions of selected expression and morphology metrics among STARIT-identified bacterial clusters. Additional boxplots showing selected supervised metrics for the four clusters identified from standardized bacterial-MERFISH *E. coli* data. (A) Distribution of total cell length in X across bacterial clusters. Boxplots display median intensity (line), interquartile range (box), and outliers, with colors denoting cluster. Statistical significance determined by Mann-Whitney U test (Bonferroni corrected). (B) Distribution of all operon expression count across bacterial clusters. Boxplots display median intensity (line), interquartile range (box), and outliers, with colors denoting cluster. Statistical significance determined by Mann-Whitney U test (Bonferroni corrected).

(Results 2.1, Lines 151-177) "Of note, while we used ResNet101, a feature extractor originally developed for natural images, for this analysis, STARIT is compatible with any downstream computer vision model for feature extraction. Although STARIT does not require the use of any specific feature extractor, feature extractor choice can influence which forms of spatial heterogeneity are emphasized. To demonstrate this, we generated an additional simulated dataset in which Groups 1 and 4 again uniquely expressed Gene 1 and Gene 3, respectively, while Groups 2 and 3 uniquely expressed Gene 2, now with left- versus right-polarized intracellular organization (Supplementary Figure 1A, Methods). Applying STARIT with ResNet101, we resolved four clusters corresponding to the four simulated groups, indicating that cell states defined by left- versus right-polarized transcript localization can be resolved (Supplementary Figure 1B). However, when we applied STARIT with UNI2-h, a feature extractor developed from a histopathology foundation model based on a vision transformer (ViT) encoder (ViT-H/14), to the same dataset, the analysis recovered three clusters, with reduced separation of the left- and right-polarized Groups 2 and 3 cells (Supplementary Figure 1C) [9]. Similar behavior was observed for CTransPath, another feature extractor for pathology images. In contrast, additional evaluated ResNet architectures including ResNet18, ResNet50, and ResNet152 more closely matched ResNet101 performance (Supplementary Table 1). This inability to distinguish left- and right-polarized groups may be expected because CTransPath, for example, is trained using frameworks that incorporate data augmentations such as random rotations and flips in learned visual representations [10]. Likewise, for UNI2-h, augmentation follows DINOv2 and other self-supervised ViT frameworks, which apply similar geometric transforms [9]. This result highlights that feature extractor choice may determine whether rotated and flipped spatial patterns such as left- versus right-polarization are preserved or diminished. Such sensitivity may be desirable when left- versus right-polarized spatial patterns is biologically meaningful, in the case of apical-basal organization, but undesirable when orientation reflects technical or arbitrary differences in cell positioning, for example. Thus, feature extractor selection should be guided by the biological question and expected sources of spatial variation in the dataset. "

(Discussion, Lines 514-519) "The choice of feature extractor is another important consideration. We primarily used ResNet101 as the feature extractor though we also evaluated alternative architectures, including smaller and larger ResNet models as well as domain-specific or foundation-model feature extractors such as CTransPath and UNI2-h (Supplementary Table 1). These comparisons suggest that feature extractor choice can influence downstream clustering and should be selected based on the biological question, data, and expected sources of variation."

(Discussion, Lines 496-513) "Feature interpretability also presents an ongoing challenge. As we've demonstrated, STARIT is compatible with any downstream computer vision model for feature extraction, but these models generally function as a "black box" with limited biological interpretability. For example, simply applying ResNet-based feature extraction to one rasterized image per gene for thousands of cells can generate a large feature matrix, with each feature potentially reflecting a mixture of signal related to transcript abundance, spatial spread, local density, shape, orientation, or potential technical artifacts. STARIT currently uses all extracted features for downstream dimensionality reduction and clustering without explicit feature selection before dimensionality reduction. While differential testing can identify features that significantly differ between identified clusters, as we have demonstrated, their biological meaning often remains open to interpretation. Likewise, while explainable AI approaches such as GradCAM and

GAINext can provide coarse localization of model attention, they do not explain why specific features drive downstream cluster assignments [28,29]. In the bacterial-MERFISH analysis, supervised metrics helped characterize STARIT-derived clusters, but no single metric fully explained cluster identity. These results suggest that while supervised metrics can improve interpretability, they function as approximations of complex latent image features. We also anticipate that future incorporation of semi-supervised statistics regarding subcellular molecular organization could help further interpret cluster differences [30–32]."

(Results 2.4, Lines 376-409) "Therefore, to interpret the differences between the clusters identified by STARIT, we applied a supervised approach to extract a set of interpretable magnitude, morphological, and spatial organizational metrics from each bacterium (Methods, Supplementary Table 2). Specifically, we computed each bacterium's normalized fliC-fliX expression magnitude and total operon expression from the original gene counts, the spatial fliC-fliX spread (in x) and cell length from the associated transcript coordinates, and the total fliC-fliX image intensity from STARIT where higher total intensity is associated with a more diffuse spatial distribution while lower total intensity is associated with a more punctated spatial organizational pattern (Methods, Supplementary Table 2). Pairwise two-sided Wilcoxon rank-sum tests on these supervised features revealed distinct combinations of magnitude, morphological, and spatial organizational properties among the 4 clusters (Figure 3C-E; Supplementary Figure 14). For example, Cluster 3 and Cluster 4 do not significantly differ in their normalized fliC-fliX expression magnitude (Figures 3E) and thus likely would not have been distinguished by conventional gene count clustering analysis. Cluster 3 does exhibits significantly greater fliC-fliX total intensity compared to than Cluster 4 but also greater spread, cell length, and total operon count (Figures 3C-D; Supplementary Figure 14A-B). As such, the spatial heterogeneity in fliC-fliX distinguishing these two clusters may be trivially explained by cell size differences. In contrast, Cluster 2 and 3 neither significantly differ in their normalized fliC-fliX expression magnitude, spread, cell length, nor total operon count (Figure 3C, E; Supplementary Figure 14A-B). Yet, they differ significantly in fliC-fliX total intensity, with Cluster 2 cells exhibiting higher total intensities than Cluster 3 cells (Figure 3B). We can thus interpret Cluster 2 cells as having a more diffuse distribution of fliC-fliX transcripts throughout the bacterium and Cluster 3 cells as having a more punctated distribution of fliC-fliX transcripts (Figure 3F). Again, these difference in subcellular spatial organization would be missed by conventional gene count clustering and highlights the ability of STARIT to resolve cells with distinct spatial heterogeneity. Importantly, we note that these supervised metrics are highly correlated with some STARIT-derived UNIh-2 extracted raw image features (spread maximum Spearman = 0.61; intensity maximum Spearman = 0.64). This correlation suggests that the high-dimensional STARIT-derived features capture these magnitude, morphological, and spatial organizational properties. Still, while fliC-fliX spread and total intensity are significantly different between our 4 identified clusters, these two supervised features do not as clearly delineate all cells across the 4 clusters as well as the UNI2-h raw extracted features (Supplementary Figure 15), suggesting that the bacterial subpopulations may be distinguished by a multivariate combination of features rather than by any two interpretable metrics."

Regarding orientation/scale invariance (c), we agree this is an important limitation of CNN-based extractors and have addressed it directly for the bacterial-MERFISH analysis by both standardizing cell orientation prior to rasterization (Supplementary Figure 11) and using UNI2-h, a non-CNN vision transformer pathology foundation-model feature extractor, which our own simulations show

is comparatively more rotation-invariant than ResNet101 (Supplementary Figure 1B-C; Results 2.4, lines 358-376; Methods 4.13-4.14). The revised text is provided below for the reviewer's convenience, along with accompanying figures showcasing results:

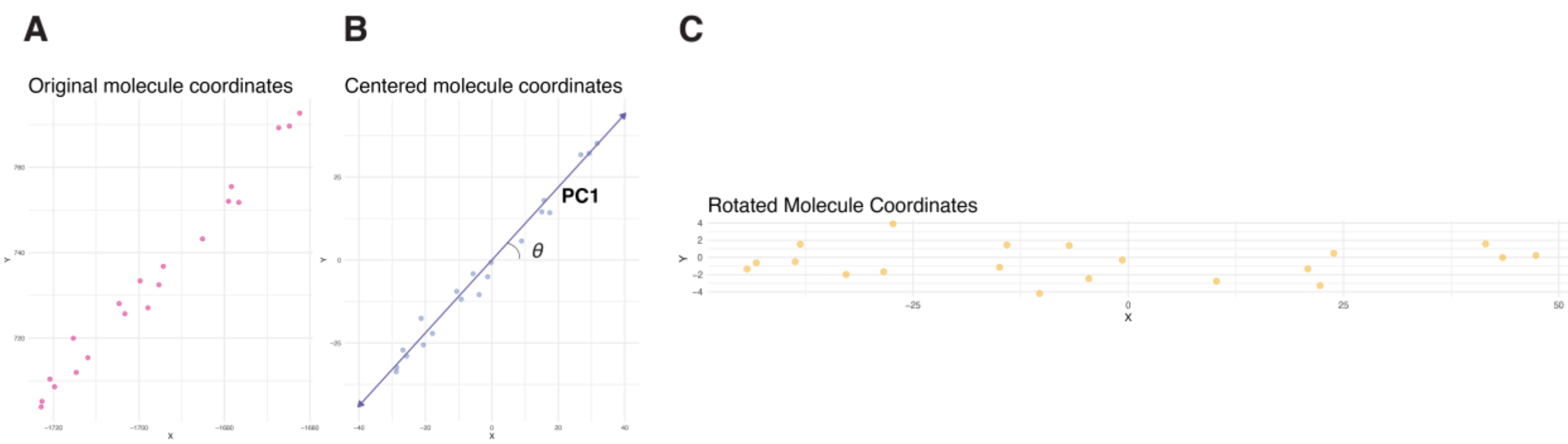

Supplementary Figure 11. Centering and principal-axis alignment of bacterial-MERFISH molecular coordinates. Visualization of the coordinate-standardization procedure for one representative *E. coli* cell. (A) Original spatial coordinates of the detected fliC-fliX molecules. (B) Molecular coordinates after centering by subtraction of the coordinate centroid. The first principal component, PC1, indicates the major axis of the molecular distribution, and θ denotes its angle relative to the horizontal axis. (C) Centered molecular coordinates after rotation by the estimated angle to align PC1 with the horizontal axis. Each point represents one detected molecule.

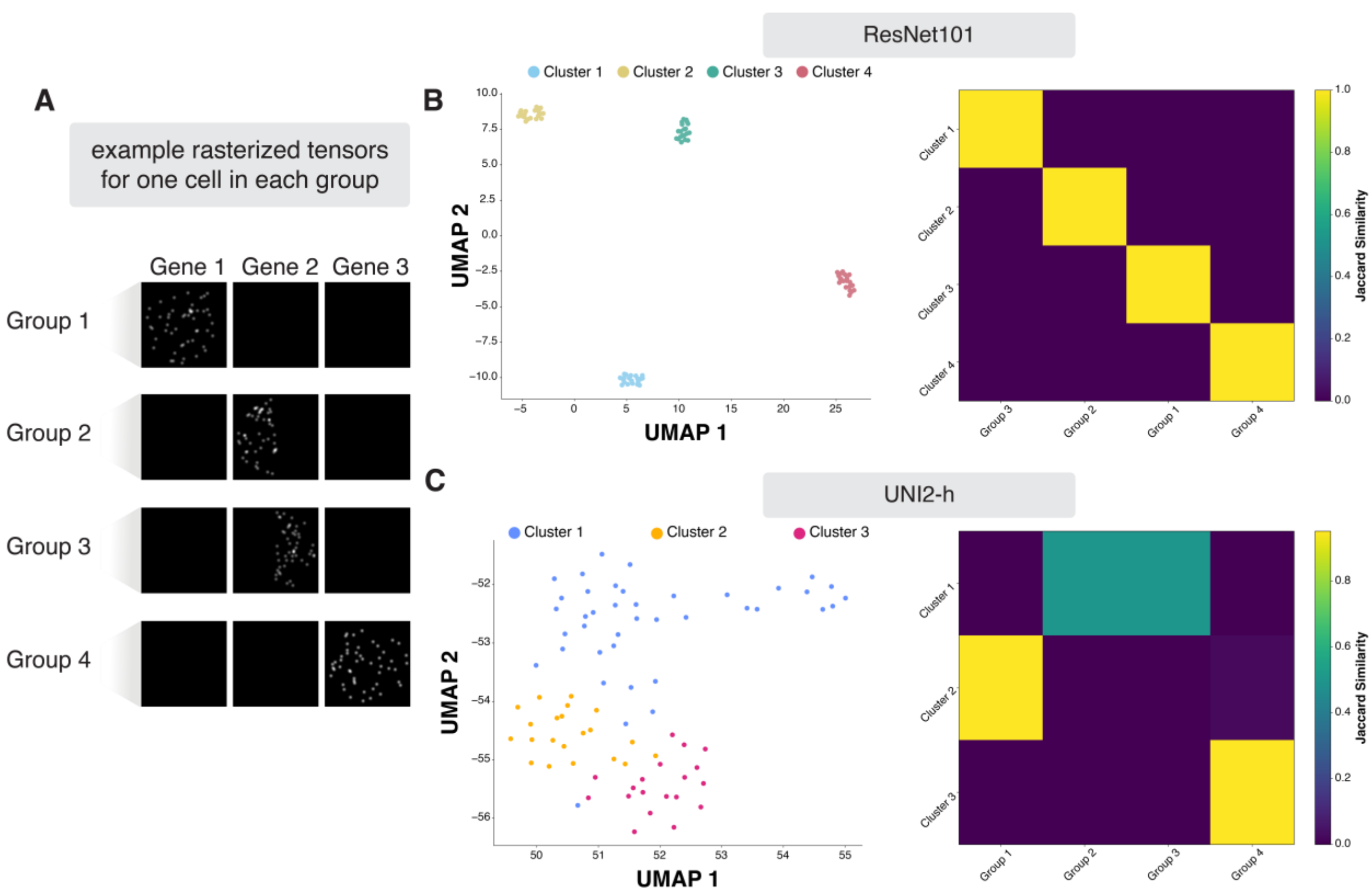

Supplementary Figure 1. Comparison of STARIT-derived ResNet101 features versus STARIT-derived UNI2-h features for left-right polarized localization simulation imSRT dataset. (A)

Representative STARIT rasterized image tensors for one cell from each of the four simulated groups. (B) Analysis results of STARIT tensors using ResNet101 feature extraction. Left: UMAP visualization based on STARIT output, embedding colored by Louvain cluster assignment. Right: Jaccard similarity heatmap between ground-truth and STARIT-based Louvain clusters. (C) Analysis results STARIT tensors using UNI2-h feature extraction. Left: UMAP visualization based on STARIT output, embedding colored by Louvain cluster assignment. Right: Jaccard similarity heatmap between ground-truth and STARIT-based Louvain clusters.

(Results 2.4, Lines 353-371) "To characterize potential biologically meaningful molecular spatial organizational heterogeneity that are not driven by the trivial rotation of the entire bacterium, we next sought to control for this rotational variation. To accomplish this, we standardized bacterial orientation across all 463 bacterial cells expressing fliC-fliX prior to rasterization (Methods). Briefly, for each bacterium, we centered all molecular coordinates and performed PCA in order to obtain the axis of maximal spatial variance, represented by the first principal component, to compute a rotation angle (θ) aligning the bacterium's major axis to the x-axis. A corresponding counterclockwise 2-D affine transformation was then applied to all molecular coordinates to standardize orientation across cells (Supplementary Figure 11). To further minimize the impact of rotational variation, we further applied STARIT with the UNI2-h feature extractor based on its previously demonstrated invariance to rotations via our simulations (Supplementary Figure 1C). We applied STARIT to these rotationally standardized molecular coordinates with UNI2-h to create a 463 cell by 1,536 feature matrix, followed by PCA and Louvain graph-based clustering. We identify 4 clusters of bacteria (Figure 3A). Again, these 4 clusters of bacteria are not obviously distinguished based on fliC-fliX magnitude (Figure 3B). When visualizing the physical bacterial coordinates of these 4 clusters in culture, we no longer observe that cells from different clusters exhibit distinct spatial rotations as expected (Supplementary Figure 12). We further perform the Wilcoxon-rank sum test on the UNI2-h features between cells in the 4 identified clusters to identify significant extracted features differentiating the 4 identified clusters (Supplementary Figure 13)."

Based on these factors, perhaps a graph neural network or point cloud-based approach makes more sense. Another idea would be to combine the gene-count data with the ResNet101 feature vector to ensure you are not losing important gene-count information that ResNet101 may not pick up effectively. I do not think it is necessary to run additional calculations but additional motivation for ResNet101 over alternatives would be beneficial.

We thank the reviewer for the suggestion. In this work, we chose an image-based representation because rasterization enables compatibility with existing pretrained computer vision models and traditional downstream pipelines and provides a standardized framework for encoding intracellular molecular spatial organization. We agree that graph neural networks and point cloud–based approaches represent promising alternative strategies for directly modeling spatial transcript coordinate data and describe them explicitly in the Discussion, citing recent related work (Discussion, lines 566-575). We additionally agree that an integration of conventional gene-count information with STARIT-derived image features would be a good idea to ensure we are not losing important gene-count information. Our existing STARIT feature-weighting strategy (Methods 4.3) already integrates gene-count magnitude information into the STARIT feature representation by multiplying each gene's image-derived features by that gene's scaled count in each cell, thereby addressing the reviewer's concern. We identify opportunities to integrate image-based

representations of imSRT data with many other data types, formats, and applications as a future direction in the Discussion (lines 577-588). The revised text is provided below for the reviewer's convenience:

(Discussion, Lines 566-575) "Although STARIT currently uses rasterized image representations, rasterization is only one strategy for encoding subcellular transcript organization. Alternative representations may directly model transcript coordinates without converting them into pixels through rasterization. Graph-based approaches have already been used in spatial omics to model spatial organization at the tissue or cellular-neighborhood level [37–39]. Likewise, such graph-based representations of transcript locations within cells has been demonstrated to improve cell type annotation [40] and cell segmentation [41]. Alternative point-cloud-based representations have also been developed to classify subcellular RNA localization patterns [42]. Future work comparing these representations can help determine which best captures biologically relevant information for specific analytical objectives."

(Discussion, Lines 576-588) "Looking forward, by providing an alternative image-based representation of imSRT data, STARIT opens opportunities to integrate image-based representations of imSRT data with many downstream applications. Emerging frameworks such as scPortrait, a scverse-native toolkit that standardizes single-cell microscopy images in an AnnData-compatible format, illustrate how STARIT and related rasterization workflows could be wrapped into interoperable packages within broader computational infrastructure [43]. More generally, as imSRT technologies advance and cell segmentation improves, and likewise computer vision models advance and interpretable feature extraction improves, we anticipate that image-based representations of spatial transcriptomics will become a useful approach for resolving subcellular organization and for generating biologically meaningful interpretations. Overall, we anticipate that STARIT will enhance the identification and characterization of novel cell-types and states by capturing subcellular molecular heterogeneity, providing deeper insights into cellular organization and functional diversity that were overlooked by gene count analysis methods alone."

3a. In section 3.4 you discuss the ability of STARIT to cluster E. coli cells based on the orientation of fliC-fliX molecules. However, given that CNNs are not rotationally invariant, it would be a surprising result if ResNet101 was unable to separate cells with different orientation. Given that the orientation was entirely and trivially driven by the cell orientation, this does not seem like a meaningful result. It would be easy to develop a method to classify cells by orientation without the use of a large DL model like ResNet101. I suggest the authors add further justification/discussion as to why this result is meaningful. Otherwise it just seems to highlight one of the biggest weaknesses of the approach (see 3b below).

3b. Building off 3a above, the fact that a trivial pattern (rotation of cells) can have such a large impact on final clusters is a reason for serious concern and highlights one key limitation of CNNs here. The authors have acknowledged this limitation in their discussion (lines 289-297). As an area of future research, I suggest addressing this issue by augmenting the data (random rotations for example) and then finetuning the model using a contrastive loss to encourage feature vectors for the same cell to be similar. In theory, this could help the approach be more invariant to rotation without any change in overall approach. Alternatively, this could be partially achieved without retraining by passing several

augmented inputs for each gene and aggregating the feature vectors (possibly average or max pooling). This is not a comment that needs to be directly addressed, but a suggestion to the authors and something potentially worth mentioning in future directions.

We thank the reviewer for raising this important issue and agree that the original (unstandardized) bacterial-MERFISH analysis with ResNet101 was substantially driven by whole-cell orientation. To characterize biologically meaningful spatial organization independent of trivial rotation, we have now standardized bacterial orientation prior to rasterization (Results 2.4, lines 353-369; Supplementary Figure 11; Methods 4.13) and additionally applied the UNI2-h feature extractor—a non-CNN, vision transformer-based pathology foundation-model feature extractor—which our left-right polarized simulation results shows is comparatively more robust to rotational variation than ResNet101 (Supplementary Figure 1B-C). This standardized analysis (Figure 3, Methods 4.14) identifies 4 clusters that are no longer explained by whole-cell rotation (Supplementary Figure 12) and are not explained by fliC-fliX expression magnitude alone (Figure 3B, E). Using supervised interpretable metrics (Methods 4.15), we show the clusters instead reflect biologically distinguishable combinations of spatial spread, intensity, and cell length (Results 2.4, lines 376-400; Figure 3C-E, Supplementary Figure 14A). For example, Clusters 2 and 3 have equivalent cell size and transcript abundance but differ significantly in total intensity, reflecting a more diffuse versus punctate fliC-fliX spatial distribution (Results 2.4, lines 397-405). We believe this addresses the reviewer's concern that the original result was trivial and have updated the Results and Discussion sections accordingly. We explicitly discuss rotational sensitivity as a key limitation of CNN-based feature extractors for imSRT data (Discussion, lines 541-553). We also thank the reviewer for the specific suggestions regarding contrastive learning, data augmentation, and feature aggregation strategies to improve rotational invariance. We now discuss these approaches as promising future directions in the Discussion section (Discussion, lines 541-553). The revised text is provided below for the reviewer's convenience as well as accompanying figures showcasing these results:

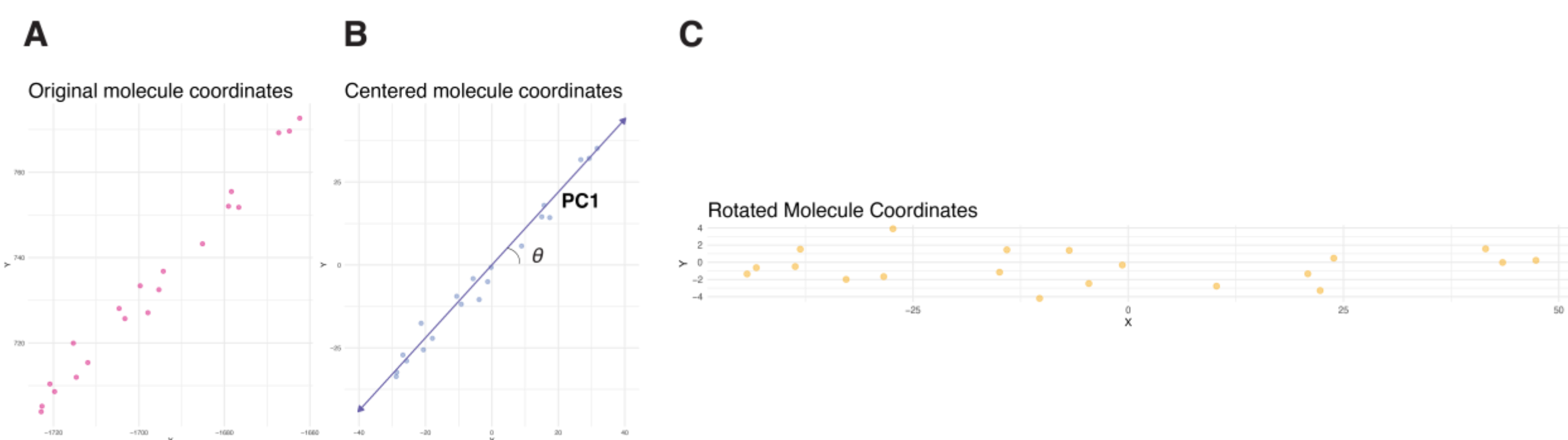

Supplementary Figure 11. Centering and principal-axis alignment of bacterial-MERFISH molecular coordinates. Visualization of the coordinate-standardization procedure for one representative *E. coli* cell. (A) Original spatial coordinates of the detected fliC-fliX molecules. (B) Molecular coordinates after centering by subtraction of the coordinate centroid. The first principal component, PC1, indicates the major axis of the molecular distribution, and θ denotes its angle relative to the horizontal axis. (C) Centered molecular coordinates after rotation by the estimated angle to align PC1 with the horizontal axis. Each point represents one detected molecule.

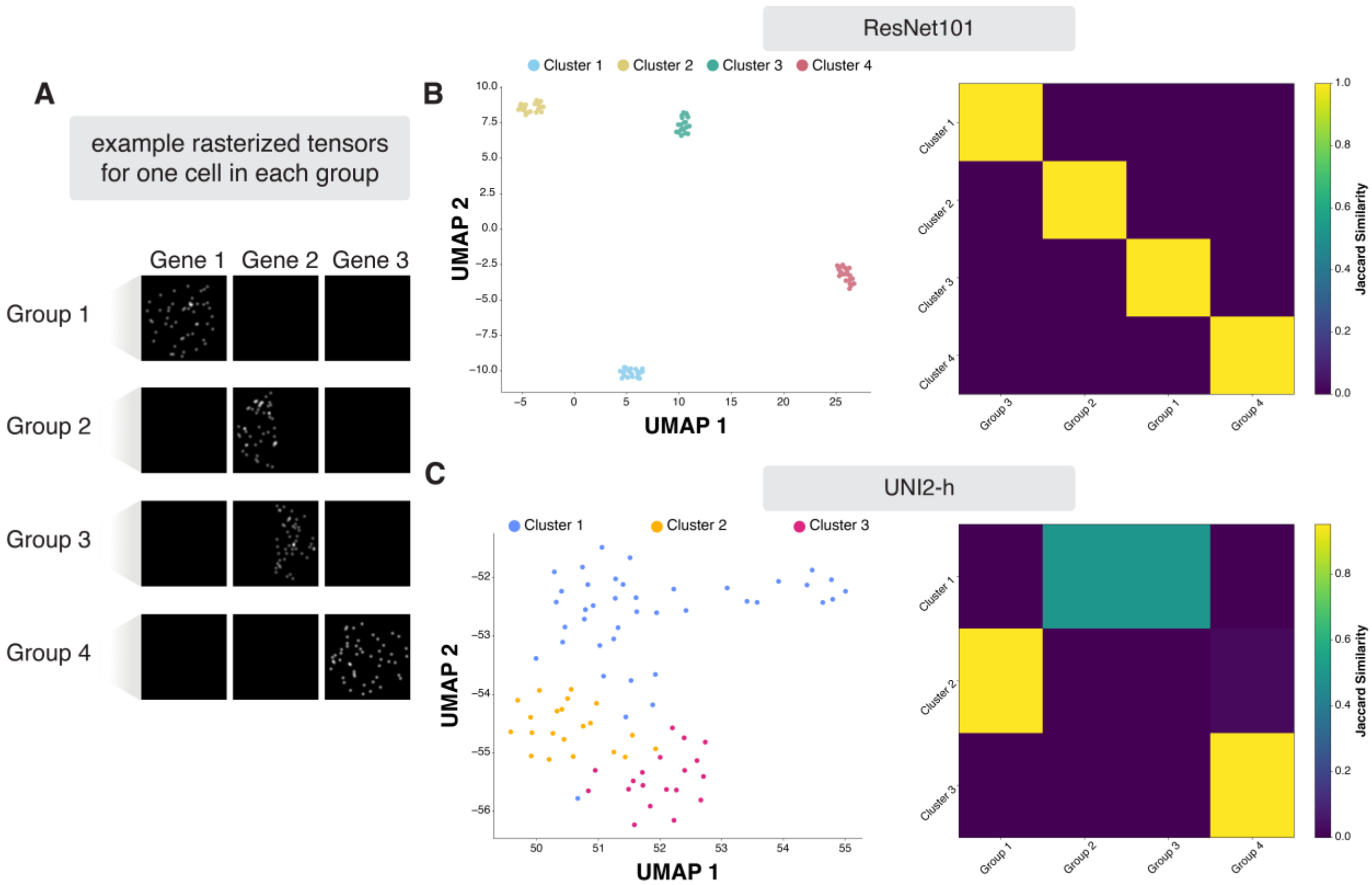

Supplementary Figure 1. Comparison of STARIT-derived ResNet101 features versus STARIT-derived UNI2-h features for left-right polarized localization simulation imSRT dataset. (A) Representative STARIT rasterized image tensors for one cell from each of the four simulated groups. (B) Analysis results of STARIT tensors using ResNet101 feature extraction. Left: UMAP visualization based on STARIT output, embedding colored by Louvain cluster assignment. Right: Jaccard similarity heatmap between ground-truth and STARIT-based Louvain clusters. (C) Analysis results STARIT tensors using UNI2-h feature extraction. Left: UMAP visualization based on STARIT output, embedding colored by Louvain cluster assignment. Right: Jaccard similarity heatmap between ground-truth and STARIT-based Louvain clusters.

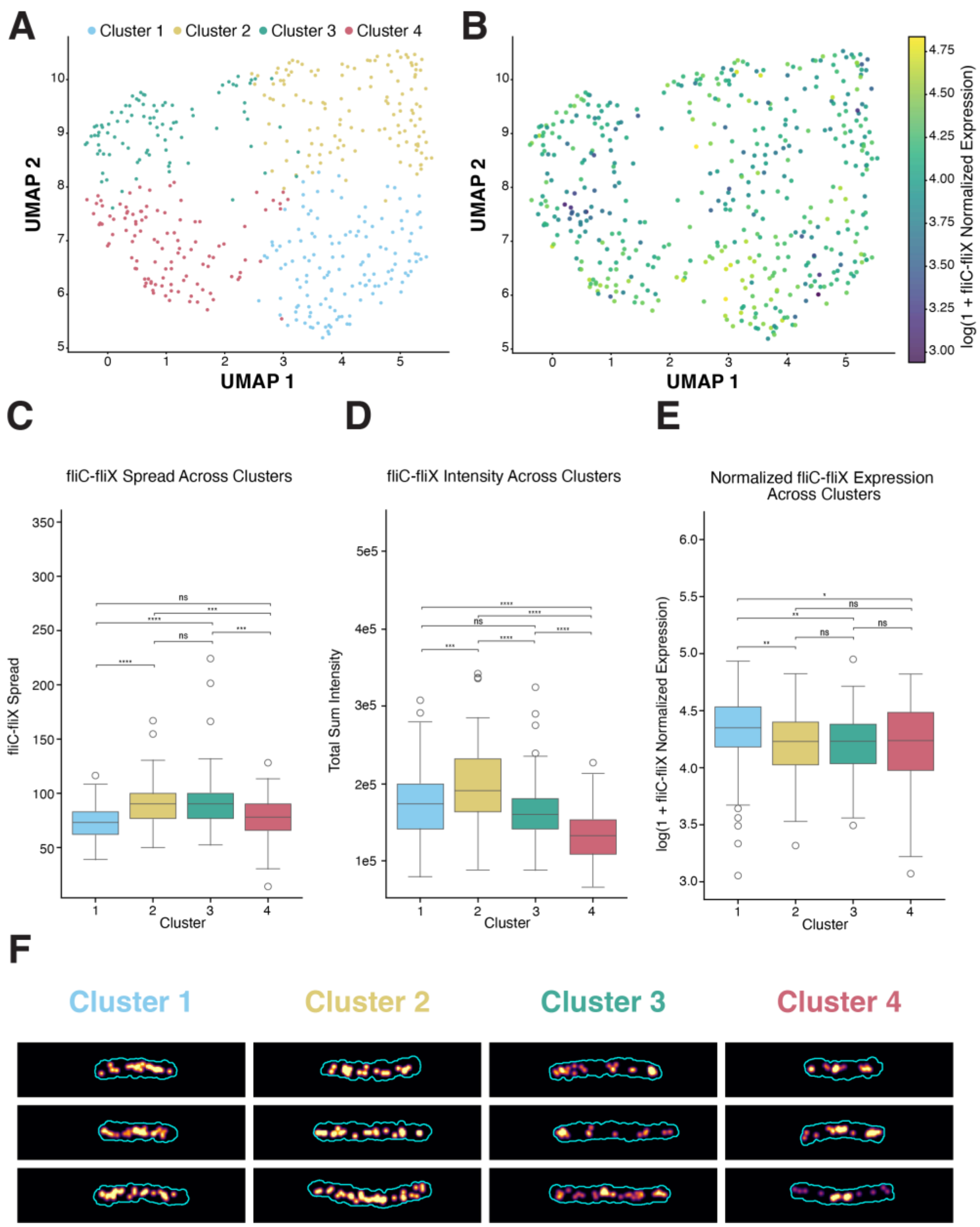

Figure 3. STARIT performance on standardized bacterial-MERFISH 1000X data. (A) Uniform Manifold Approximation and Projection (UMAP) of STARIT-derived UNI2-h features from rotationally standardized 463 *E. coli* cells based fliC-fliX operon localization, imaged with bacterial-MERFISH at 1000X volumetric expansion. (B) UMAP embedding of cells colored by log-transformed fliC-fliX gene expression levels. (C) Distribution of variation in normalized fliC-fliX gene expression across bacterial clusters. Expression levels normalized to total molecular counts per 1000 and log-transformed. Boxplots display median intensity (line), interquartile range (box), and outliers, with colors denoting cluster. Statistical significance determined by Mann-Whitney U test (Bonferroni corrected). (D) Distribution of spatial spread in X of fliC-fliX across bacterial clusters. Boxplots display median intensity (line), interquartile range (box), and outliers, with colors denoting cluster. Statistical significance determined by Mann-Whitney U test (Bonferroni corrected). (E) Distribution of total fliC-fliX signal across bacterial clusters. Boxplots display median intensity (line), interquartile range (box), and outliers, with colors denoting cluster. Statistical

significance determined by Mann-Whitney U test (Bonferroni corrected). (F) Select examples of rasterized fliC-fliX transcript distributions for cells in clusters 1-4.

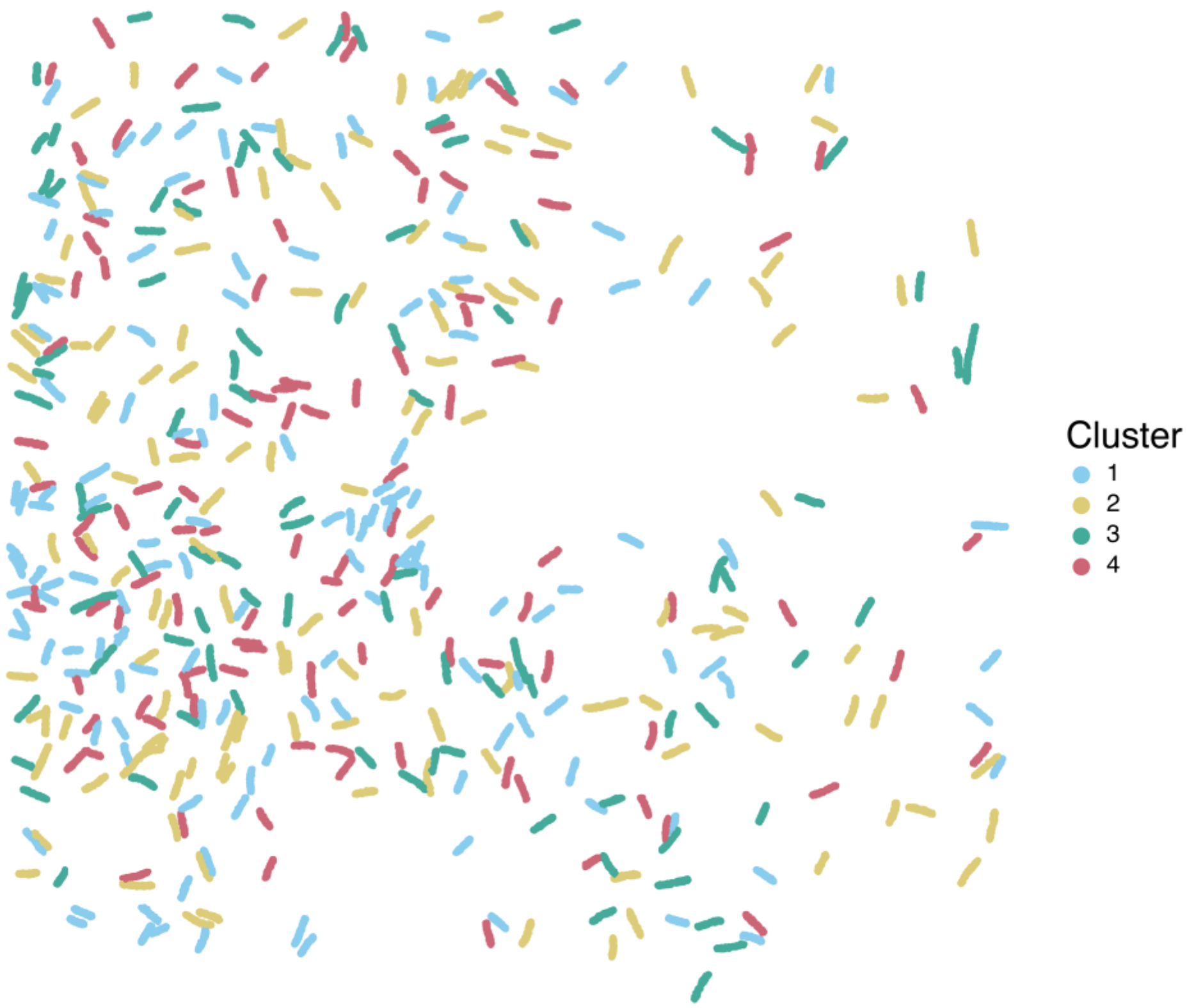

Supplementary Figure 12. Physical-space visualization of standardized STARIT-identified clusters in Bacterial-MERFISH *E. coli* data. Shown are the spatial positions of 463 *E. coli* cells assayed by Bacterial-MERFISH after 1000-fold volumetric expansion, plotted in their original physical coordinates and colored by the four Louvain clusters identified from STARIT-derived UNI2-h image features.

A

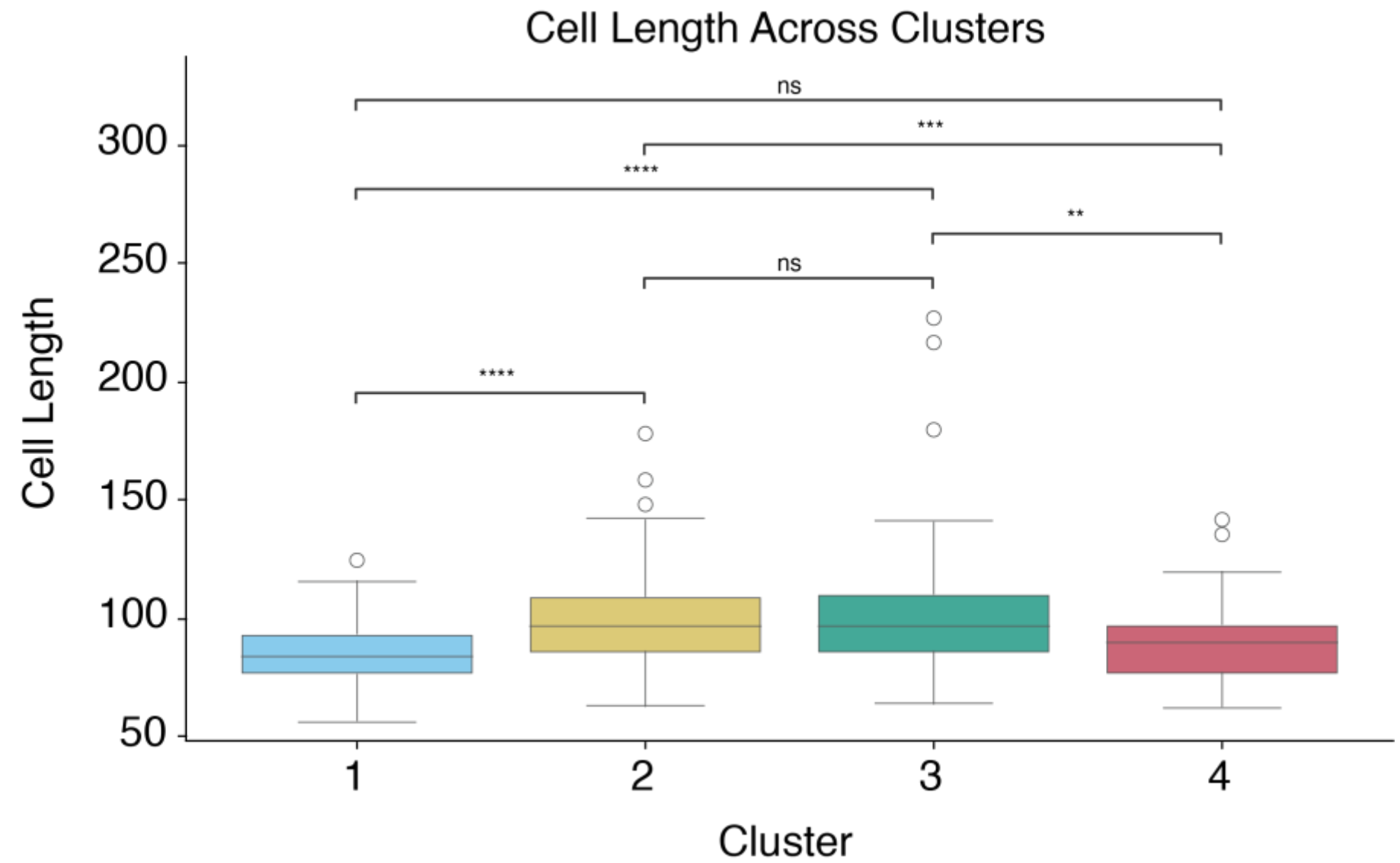

B

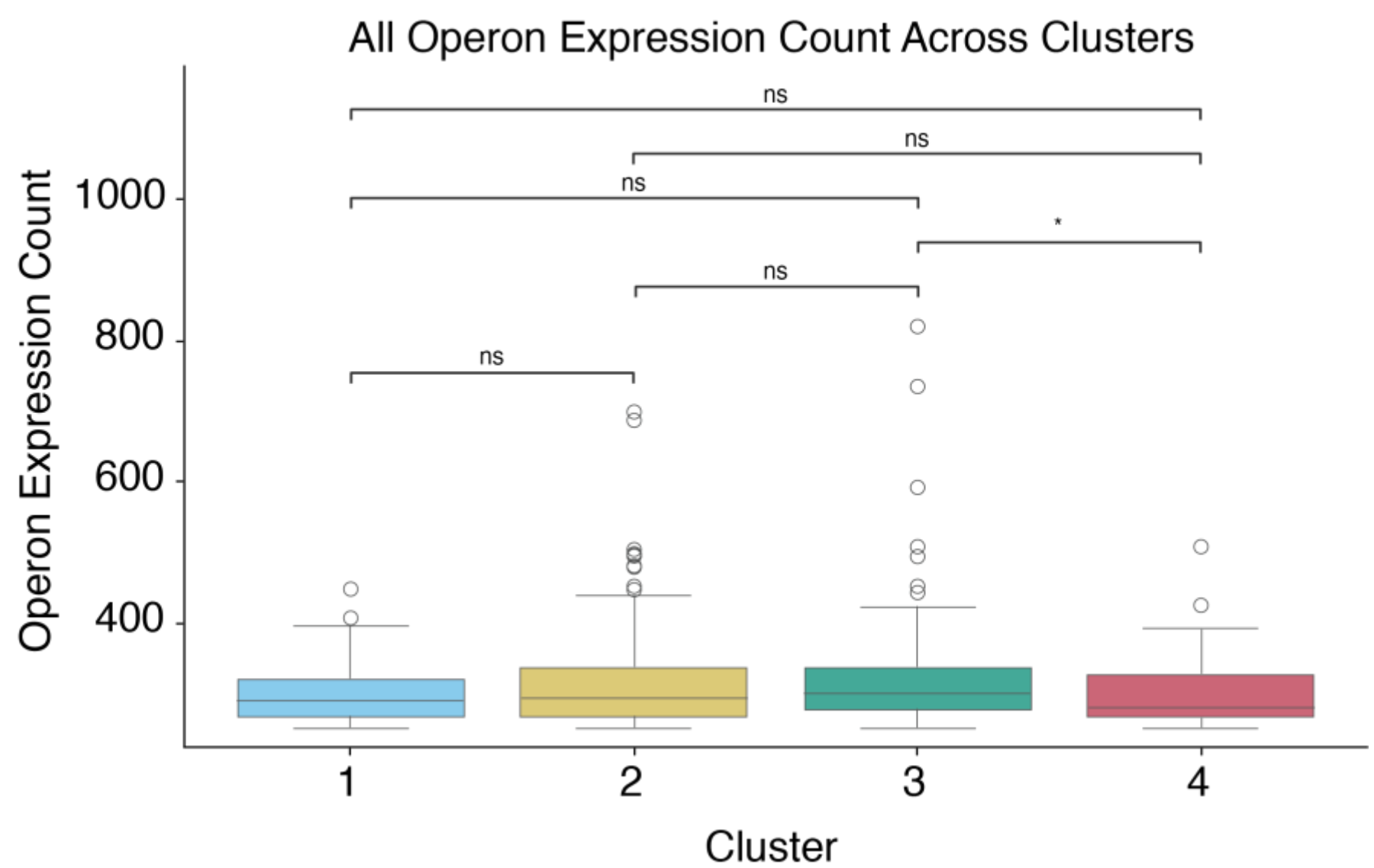

Supplementary Figure 14. Distributions of selected expression and morphology metrics among STARIT-identified bacterial clusters. Additional boxplots showing selected supervised metrics for the four clusters identified from standardized bacterial-MERFISH *E. coli* data. (A) Distribution of total cell length in X across bacterial clusters. Boxplots display median intensity (line), interquartile range (box), and outliers, with colors denoting cluster. Statistical significance determined by Mann-Whitney U test (Bonferroni corrected). (B) Distribution of all operon expression count across bacterial clusters. Boxplots display median intensity (line), interquartile range (box), and outliers, with colors denoting cluster. Statistical significance determined by Mann-Whitney U test (Bonferroni corrected).

(Results 2.4, Lines 353-369) "To characterize potential biologically meaningful molecular spatial organizational heterogeneity that are not driven by the trivial rotation of the entire bacterium, we

next sought to control for this rotational variation. To accomplish this, we standardized bacterial orientation across all 463 bacterial cells expressing fliC-fliX prior to rasterization (Methods). Briefly, for each bacterium, we centered all molecular coordinates and performed PCA in order to obtain the axis of maximal spatial variance, represented by the first principal component, to compute a rotation angle (θ) aligning the bacterium's major axis to the x-axis. A corresponding counterclockwise 2-D affine transformation was then applied to all molecular coordinates to standardize orientation across cells (Supplementary Figure 11). To further minimize the impact of rotational variation, we further applied STARIT with the UNI2-h feature extractor based on its previously demonstrated invariance to rotations via our simulations (Supplementary Figure 1C). We applied STARIT to these rotationally standardized molecular coordinates with UNI2-h to create a 463 cell by 1,536 feature matrix, followed by PCA and Louvain graph-based clustering. We identify 4 clusters of bacteria (Figure 3A). Again, these 4 clusters of bacteria are not obviously distinguished based on fliC-fliX magnitude (Figure 3B). When visualizing the physical bacterial coordinates of these 4 clusters in culture, we no longer observe that cells from different clusters exhibit distinct spatial rotations as expected (Supplementary Figure 12)."

(Results 2.4, Lines 376-400) "Therefore, to interpret the differences between the clusters identified by STARIT, we applied a supervised approach to extract a set of interpretable magnitude, morphological, and spatial organizational metrics from each bacterium (Methods, Supplementary Table 2). Specifically, we computed each bacterium's normalized fliC-fliX expression magnitude and total operon expression from the original gene counts, the spatial fliC-fliX spread (in x) and cell length from the associated transcript coordinates, and the total fliC-fliX image intensity from STARIT where higher total intensity is associated with a more diffuse spatial distribution while lower total intensity is associated with a more punctated spatial organizational pattern (Methods, Supplementary Table 2). Pairwise two-sided Wilcoxon rank-sum tests on these supervised features revealed distinct combinations of magnitude, morphological, and spatial organizational properties among the 4 clusters (Figure 3C-E; Supplementary Figure 14). For example, Cluster 3 and Cluster 4 do not significantly differ in their normalized fliC-fliX expression magnitude (Figures 3E) and thus likely would not have been distinguished by conventional gene count clustering analysis. Cluster 3 does exhibits significantly greater fliC-fliX total intensity compared to than Cluster 4 but also greater spread, cell length, and total operon count (Figures 3C-D; Supplementary Figure 14A-B). As such, the spatial heterogeneity in fliC-fliX distinguishing these two clusters may be trivially explained by cell size differences. In contrast, Cluster 2 and 3 neither significantly differ in their normalized fliC-fliX expression magnitude, spread, cell length, nor total operon count (Figure 3C, E; Supplementary Figure 14A-B). Yet, they differ significantly in fliC-fliX total intensity, with Cluster 2 cells exhibiting higher total intensities than Cluster 3 cells (Figure 3B). We can thus interpret Cluster 2 cells as having a more diffuse distribution of fliC-fliX transcripts throughout the bacterium and Cluster 3 cells as having a more punctated distribution of fliC-fliX transcripts (Figure 3F). Again, these difference in subcellular spatial organization would be missed by conventional gene count clustering and highlights the ability of STARIT to resolve cells with distinct spatial heterogeneity."

(Discussion, Lines 541-553) "A related limitation of ResNet architectures is rotational and scale sensitivity. Conventional convolutional neural networks, including ResNet models, are not inherently invariant to image orientation or scale. In the bacterial-MERFISH analysis, the original orientation of cells influenced extracted image features, highlighting both a limitation and a

diagnostic property of image-based representations. To address this, we rotationally standardized cellular orientation before rasterization. Additional robustness could be achieved at the feature extraction step itself, for example by fine-tuning the existing pretrained feature extractor with random rotation and scale augmentations, utilizing contrastive learning to encourage augmented views of the same rasterized gene images within cells or cell states to produce similar feature vectors, or adopting rotation-invariant architectures or equivariant neural networks as feature extractors [33–35]. These strategies may be particularly important for tissues with polarized structures, such as epithelia with apical–basal organization, where rotation may represent either a confounder or a biologically meaningful axis depending on the analysis goal."

The previous rotation-unstandardized results have been moved to the Supplementary Material (Supplementary Figures 9-10), shown below for the reviewer's convenience:

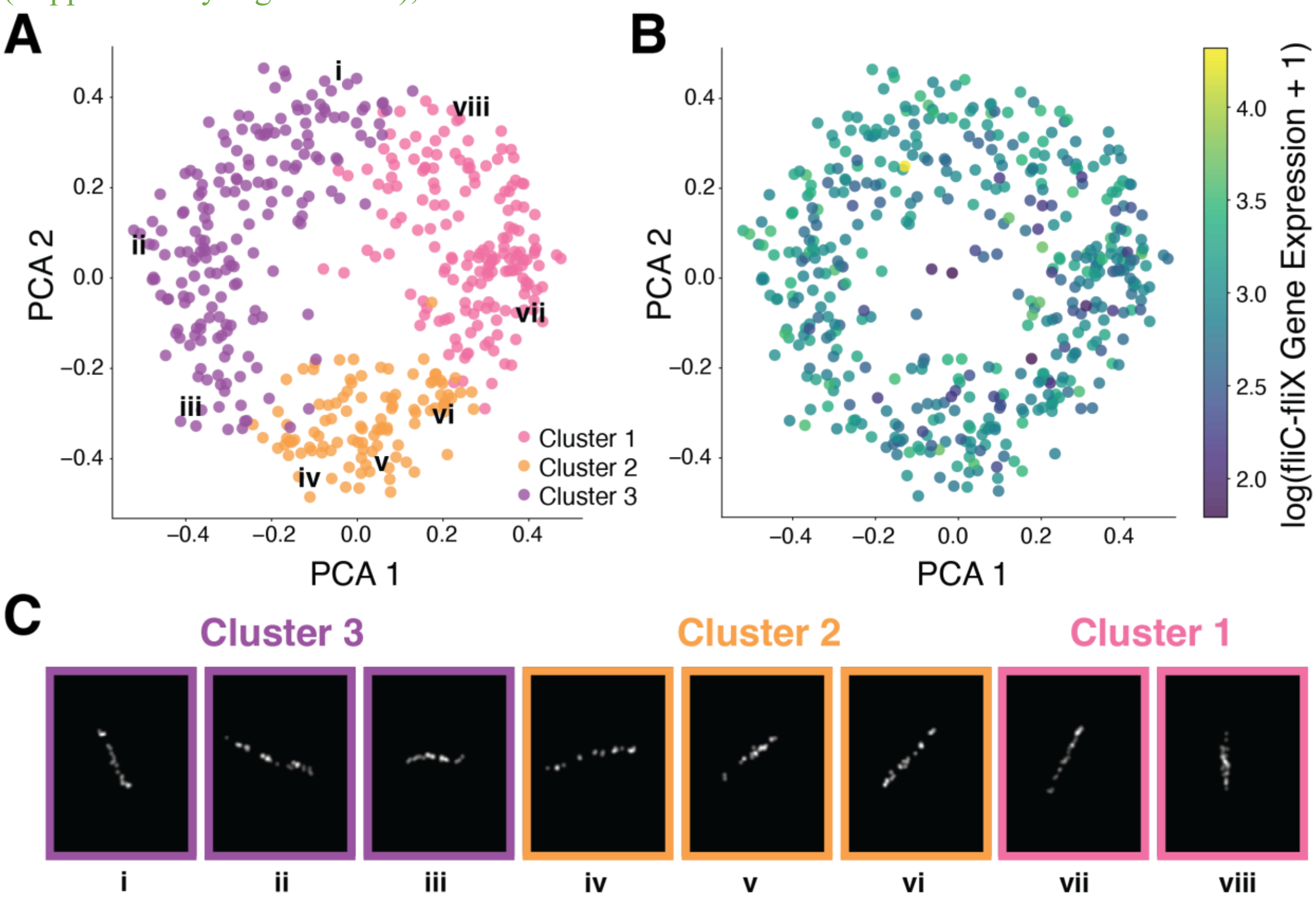

Supplementary Figure 9. STARIT performance on unstandardized bacterial-MERFISH 1000X data. (A) Principal component analysis (PCA) of STARIT-derived features from 463 *E. coli* cells based on fliC–fliX operon localization, imaged with bacterial- MERFISH at 1000X volumetric expansion. Cells are colored by Louvain graph-based cluster assignment: Cluster 1 (pink), Cluster 2 (orange), Cluster 3 (purple). Select labeled cells (i–viii) correspond to examples shown in panel C. (B) PCA embedding of cells colored by log-transformed fliC–fliX gene expression levels. (C) Representative rasterized fliC–fliX transcript distributions for cells i–viii labeled in panel A, with frame colors indicating cluster assignment.

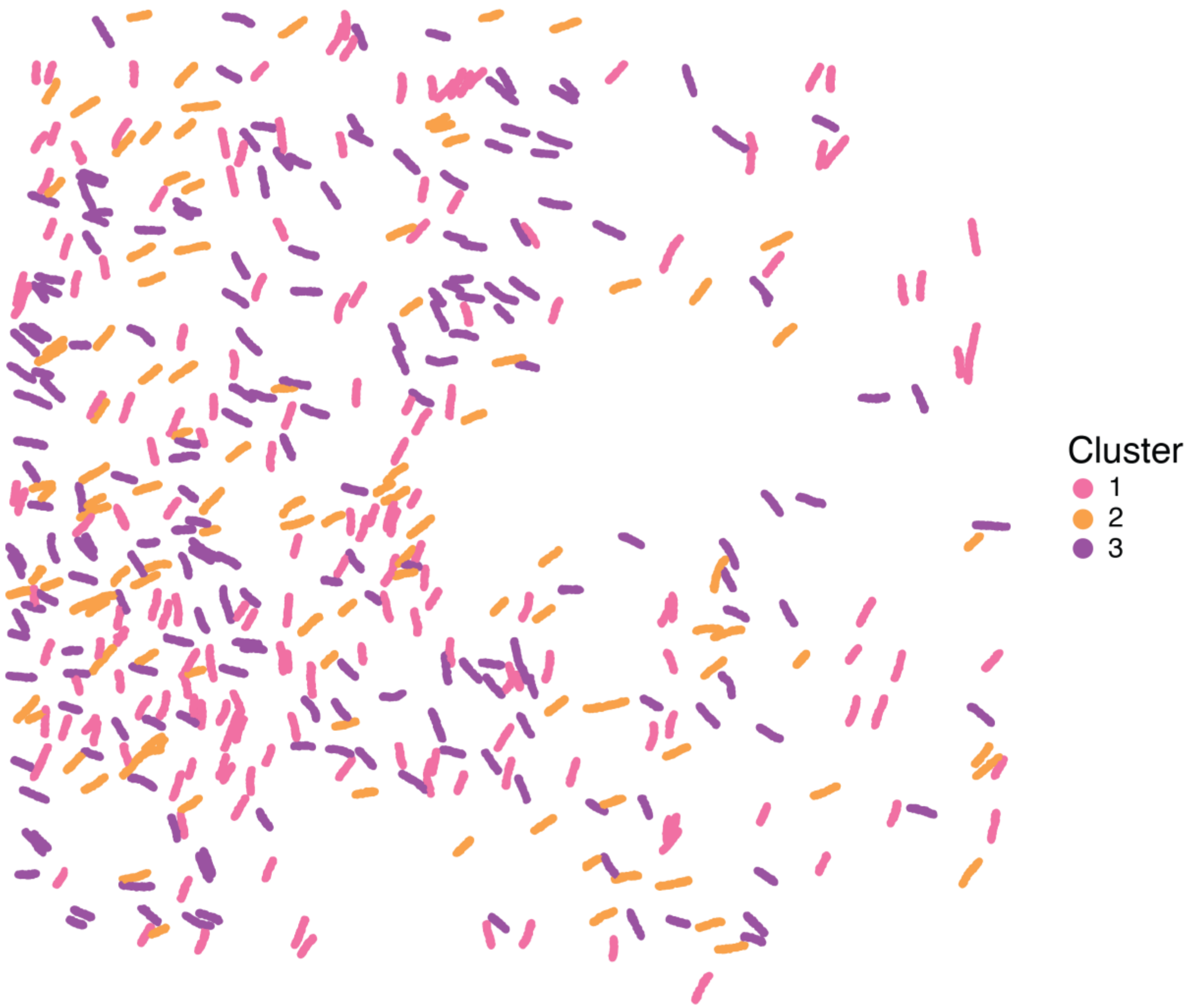

Supplementary Figure 10. Physical-space visualization of unstandardized STARIT-identified clusters in Bacterial-MERFISH *E. coli* data. Shown are the spatial positions of 463 *E. coli* cells assayed by Bacterial-MERFISH after 1000-fold volumetric expansion, plotted in their original physical coordinates and colored by the three Louvain clusters identified from STARIT-derived ResNet101 image features.

Minor comments:

4. The terminology of "Cluster 1-1", "Cluster 1-0", etc. is not really clear. It seems that the first number denotes gene-count (1) vs STARIT (2) and the second number denotes the cluster index. Perhaps use something like Cluster_GC-1 for the first gene-count cluster would make things more clear. If you keep the current labeling, I suggesting making it more clear. It also seems strange that the approach indexing starts at 1 while the cluster indexing starts at 0.

We thank the reviewer for the suggestion. We have modified the cluster terminology labeling throughout for clarity. Clusters are now referred to simply as “Cluster 1,” “Cluster 2,” etc., with consistent 1-based indexing, and the specific analysis (STARIT vs. gene-count) is always stated explicitly in the surrounding text (see, e.g., Figure 1D-G, Figure 2, Figure 3, Supplementary Figures 5-6).

Reviewer #3

Summary
In this work, Velazquez et al introduce STARIT, a workflow for cell type assignment and clustering from imaging based spatial transcriptomics data. They demonstrate the power of STARIT using simulated data and real world MERFISH data. The major contribution of STARIT is that it is able to capture cell types and states defined by differences in the spatial arrangement of molecules within cells (for example cells with distinct nuclear vs perinuclear molecular localization). Overall, I think the method is a useful contribution to the field and provide some specific comments below:

1. Spatial transcriptomics methods are often applied to 4–5-micron thick tissue sections. In any given section there are fully intact cells, as well as majority and minority intact cells where only part of the nucleus and cytoplasm are captured. STARIT, a method that uses spatial localization of molecules to define cell types and states, may be particularly sensitive to this problem. I suggest the authors conduct another simulated data experiment where they randomly cut pieces from cells and determine how well STARIT can still correctly call cell types. How does the performance of STARIT on this data compare to standard methods?

We thank the reviewer for the suggestion. In response, we generated three additional simulated imSRT datasets modeling partial transcript capture from sectioning, each containing cells with a full complement of 80 detected molecules alongside partially-captured cells retaining 40 (50%), 20 (25%), or 10 (12.5%) molecules of the same underlying nuclear, perinuclear, or diffuse localization pattern (simulated datasets #5-7; Methods 4.4.5; Supplementary Table 3). This models proportional under-sampling of an intact spatial pattern, consistent with a thin section intersecting a smaller fraction of a cell’s volume, rather than a spatial crop of the image itself (e.g., capturing only one half or the top of a cell). We discuss this distinction, and the complementary spatial truncation scenario as a direction for future evaluation, in the revised Discussion (lines 468-472). STARIT with ResNet101 continued to recover the expected localization-defined groups at 50% and 25% retained volume, indicating robustness to moderate transcript loss, but performance degraded at 12.5% retained volume, where sparse molecular sampling no longer reliably preserved the underlying spatial pattern (Results 2.1, lines 190-204; Supplementary Figure 3).

Based on these results, we now explicitly recommend in the Discussion that users apply quality-control filtering based on transcript abundance and/or segmented cell volume prior to applying STARIT, and note that STARIT’s core assumption is that the observed 2D transcript snapshot contains sufficient information to approximate the underlying subcellular organization, which weakens as captured volume shrinks (Discussion, lines 472-480). We also emphasize that in

practice, cells retaining as few transcripts as our 12.5% volumetric-capture setting would likely already be excluded by standard single-cell/spatial QC pre-filtering in most pipelines, meaning STARIT's weaker performance at very low counts is a regime that good quality control already screens against. The revised text is provided below for the reviewer's convenience as well as an accompanying figure showcasing results (Supplementary Figure 3):

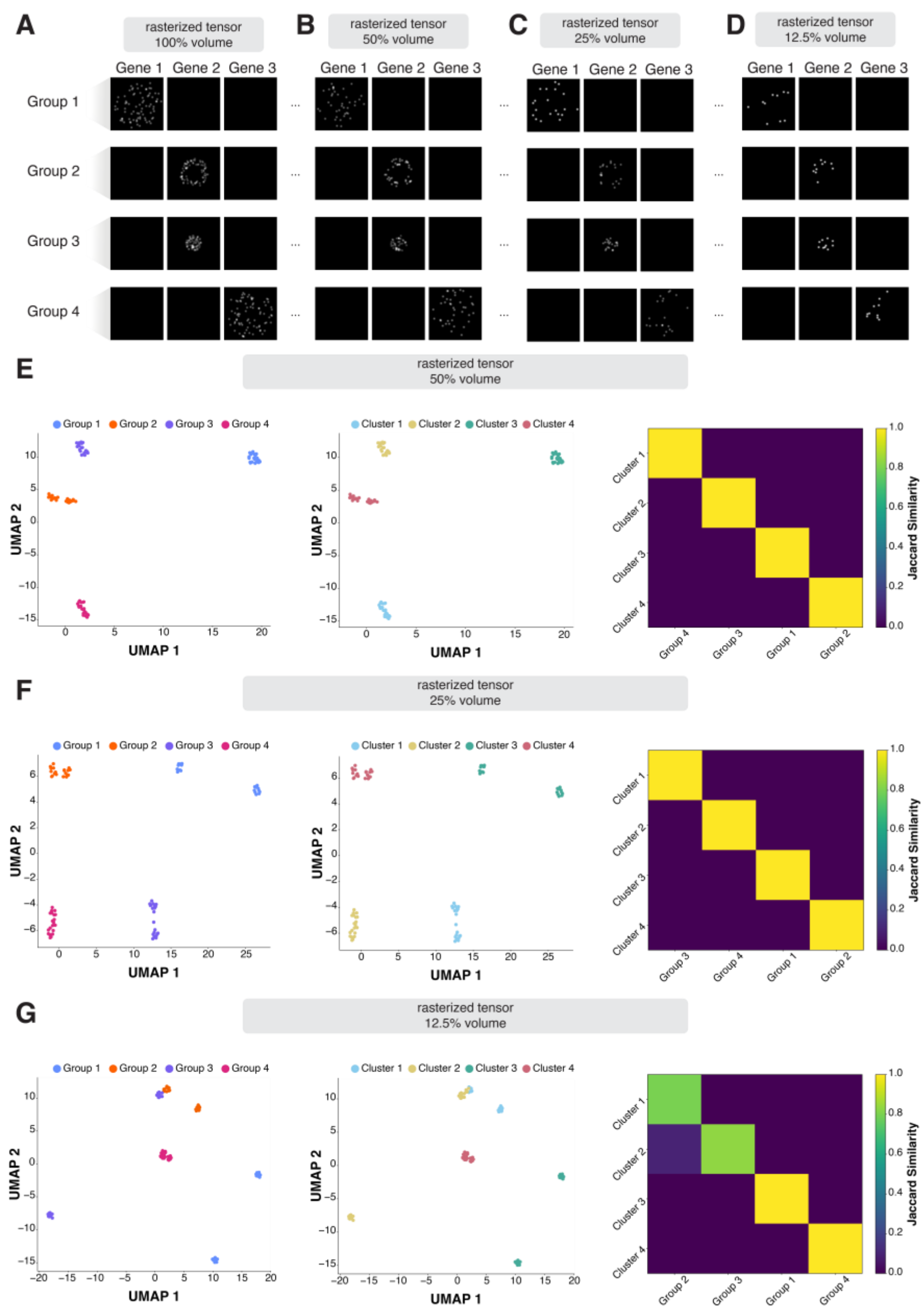

Supplementary Figure 3. STARIT-derived ResNet101 features for simulated cut-volume molecule count localization datasets. (A-D) Representative STARIT rasterized image tensors for one cell from each simulated group at (A) 100%, (B) 50%, (C) 25%, and (D) 12.5% retained cell volume. Within each panel, columns correspond to Gene 1, Gene 2, and Gene 3, and rows correspond to the four simulated groups. (E-G) Clustering analyses for simulations with (E) 50%, (F) 25%, and (G) 12.5% retained cell volume. (E) Left: UMAP visualization based on ground-truth, with embedding colored by group. Middle:

UMAP visualization based on STARIT output, with embedding colored by Louvain cluster assignment. Right: Jaccard similarity heatmap between ground-truth and STARIT-based Louvain clusters. (F) Left: UMAP visualization based on ground-truth, with embedding colored by group. Middle: UMAP visualization based on STARIT output, with embedding colored by Louvain cluster assignment. Right: Jaccard similarity heatmap between ground-truth and STARIT-based Louvain clusters. (G) Left: UMAP visualization based on ground-truth, with embedding colored by group. Middle: UMAP visualization based on STARIT output, with embedding colored by Louvain cluster assignment. Right: Jaccard similarity heatmap between ground-truth and STARIT-based Louvain clusters.

(Results 2.1, Lines 190-204) "Finally, a common challenge with current imSRT technologies, particularly in a tissue-setting, is that a whole cell may not always be captured in the associated thin tissue section. When a partial cell volume is captured, proportionally fewer transcripts may be detected. As such, we sought to evaluate whether STARIT remains robust when variable fractions of the cell volume is captured. We therefore generated additional simulated imSRT datasets to approximate partial cell volume capture in half the cells by systematically reducing molecule counts to represent 50%, 25%, and 12.5% volumetric capture variants, respectively (Supplementary Figure 3A-D, Methods). STARIT recovered the expected localization-defined groups in the 50% and 25% volumetric capture simulations, indicating robustness to moderate volumetric capture variation (Supplementary Figure 3E-F). However, performance degraded in the 12.5% volumetric capture setting, where sparse molecular sampling likely limited reliable recovery of subcellular localization patterns (Supplementary Figure 3G). These results suggest that STARIT can tolerate moderate reductions in transcript detection due to decreased volumetric capture. Based on these simulations, we recommend appropriate quality control filtering to remove cells based on low transcript abundance or segmented cell volume prior to applying STARIT."

(Discussion, Lines 466-480) "Likewise, thin tissue sections currently used in imSRT introduce an additional source of variation because measurements may capture fully intact cells as well as partially sectioned cells with reduced volume. Our partial volume capture simulations suggest that STARIT can tolerate moderate transcript loss when the underlying localization pattern remains sufficiently represented. Still, sectioning through a cell may remove transcriptionally distinct structures to alter observed transcript localization patterns beyond transcript loss. Evaluating the robustness of STARIT under spatial truncation scenarios remains a direction for future work. Nevertheless, as with other single-cell and spatial analysis methods, these results demonstrate how applications of STARIT to thin tissue sections will require quality control filtering to remove cells based on low transcript counts or small captured cell volumes. More generally, STARIT assumes that the observed two-dimensional transcript pattern snapshot contains enough information to approximate biologically meaningful subcellular organization. While this assumption may be weakened when only a small fraction of the cell volume is captured, ongoing advances in imSRT technology development toward applications in thicker tissue sections will strengthen its validity [22–25]."

2. How well does STARIT apply to diseased tissues where cell states are not discrete but exist along a continuum? For example, cancer cells treated with chemotherapy may possess a heterogeneous mix of responding and resistant characteristics. How can this be captured using the STARIT method?

We thank the reviewer for the question. In response, we generated an additional simulated dataset (simulated dataset #4; Methods 4.4.4) in which cells contain a constant total of 80 transcripts of a single gene—the total transcript count per cell does not vary—while the fraction of those 80 molecules assigned to a perinuclear annulus versus the nucleus increases monotonically from 0% to 100% across the cell population. This isolates a continuous shift in subcellular spatial organization at fixed expression magnitude, distinct from any change in overall transcript abundance. Applying STARIT to this dataset, the resulting embeddings preserved the gradual progression of this localization trajectory (Results 2.1, lines 178-189; Supplementary Figure 2), suggesting STARIT can capture continuous variation in subcellular molecular organization in addition to discrete cell states.

We now discuss potential applications of STARIT to continuous biological processes such as disease progression and treatment response in the Discussion (lines 481-486). The revised text is provided below for the reviewer's convenience as well as an accompany figure showcasing these results (Supplementary Figure 2):

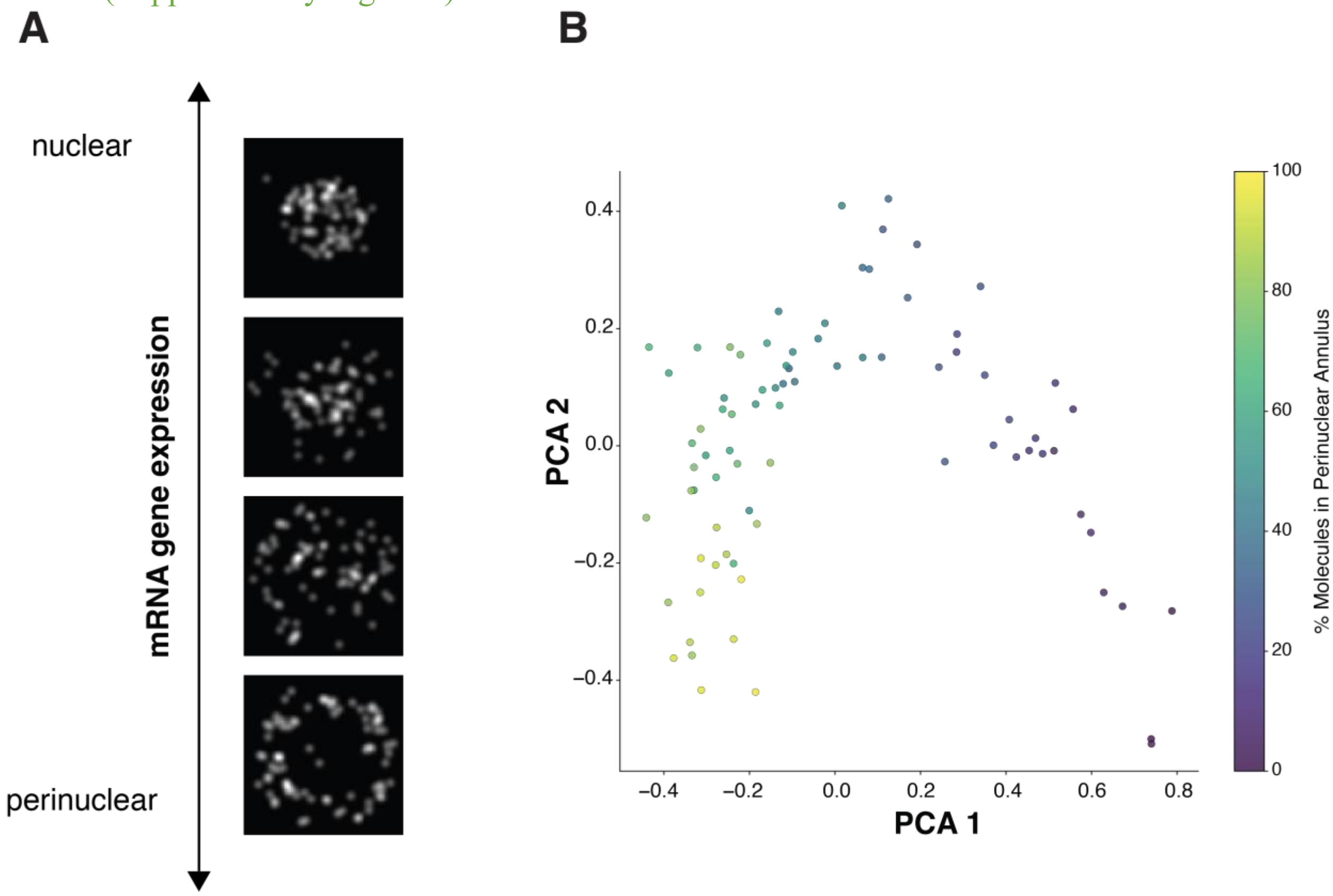

Supplementary Figure 2. STARIT-derived ResNet101 features for the nuclear-perinuclear continuum simulation dataset. (A) Representative cell examples from the nuclear-perinuclear continuum simulation dataset, illustrating the spatial transition of mRNA transcript localization from the nucleus (top) to the outer perinuclear annulus (bottom). (B) Principal Component Analysis (PCA) projection of extracted ResNet101 features across simulated cells, colored by the percentage of molecules localized to the perinuclear annulus.

(Results 2.1, Lines 178-189) "The simulated datasets thus far evaluate STARIT in settings where cell states are represented as discrete. However, cell states may vary continuously across

developmental, environmental, or disease-associated trajectories [11,12]. We therefore sought to evaluate whether STARIT could capture continuous variation in subcellular organization. To model a continuous localization trajectory, we simulated cells with molecules sampled from either a circular nuclear region or a surrounding perinuclear annulus where the fraction of molecules assigned to the annulus increased monotonically from 0% to 100% across cells (Supplementary Figure 2A, Methods). As such, this simulation generated a gradual transition from nuclear to perinuclear transcript localization while holding total expression magnitude constant. STARIT preserved this gradual progression in various dimensional embedding spaces, suggesting that STARIT can capture continuous trajectories represented in a gradual transition of intracellular transcript organization (Supplementary Figure 2B)."

(Discussion, Lines 481-486) "Moving beyond snapshots, many biological processes — such as disease progression, differentiation, and treatment response — progress continuously. Our nuclear-perinuclear continuum simulation suggests that STARIT-derived feature embeddings can preserve gradual transitions in subcellular transcript organization in addition to discrete states. Thus, evaluating STARIT on real imSRT datasets to discover such continuous transitions in subcellular organization along trajectories remains a potential direction for future exploration."

3. The authors demonstrated their method on simulated data as well as two FISH data sets. Can they demonstrate the applicability of their software on another imSRT dataset such as Xenium and comment on how the performance is similar or different to MERFISH?

We thank the reviewer for the suggestion. While a full Xenium analysis is beyond the scope of the current study, we have expanded the Discussion to clarify that STARIT is designed as a general framework compatible with multiple imSRT technologies, including Xenium, MERFISH, seqFISH, CosMx, and related coordinate-based technology platforms. We additionally discuss important platform-specific considerations—segmentation quality, transcript detection sensitivity, probe specificity, and off-target detection—that can alter the apparent subcellular distribution of transcripts and therefore may influence downstream image-based feature extraction. We have cited recent work characterizing technical artifacts, off-target probe binding, and segmentation challenges across imSRT technologies, and discussed how these factors may impact STARIT analyses (Discussion, lines 4872-495). We further note that applying STARIT to Xenium and other large-scale imSRT datasets represents an important future direction for evaluating platform-specific robustness and discovering biologically meaningful subcellular heterogeneity. The revised text is provided below for the reviewer's convenience:

(Discussion, Lines 487-495) "STARIT is designed as a general framework for coordinate-based imSRT technologies and can in principle be applied across technologies including but not limited to MERFISH, seqFISH, Xenium, CosMx, and related platforms. However, platform-specific technical properties may influence performance. For example, differences in segmentation strategy, transcript detection sensitivity, probe specificity, and off-target detection can alter the apparent subcellular distribution of transcripts [21,26,27]. As such, the robustness of STARIT to platform-specific technical variation represents an important future direction for determining whether image-based transcript localization features can consistently reveal biologically meaningful subcellular heterogeneity across platforms."

Reviewer #4

Summary

The authors have developed the STARIT framework to identify cell states using subcellular molecular information derived from imSRT data. The framework converts transcripts within segmented cells in imaging data into a tensor representation that can be combined with deep learning computer vision models for downstream analysis. It provides deeper insights into subcellular heterogeneity and improves the identification and characterization of cell types and states that may be overlooked when using only gene-count representations.

Major Issues:

1. **The Introduction lacks why authors have developed STARIT and how it is better than already existing methods. The section needs more reasoning and background on the existing tools and their limitations.**

We thank the reviewer for bringing attention to the issue. In lines 80-99, we provide background on existing methods and explain the limitation of conventional gene count-based analyses and the motivation behind STARIT, to incorporate the subcellular organization of mRNA transcripts to better characterize cellular identity and uncover new cellular states. We have moved a section from Methods to reiterate the knowledge gap STARIT addresses, including citing evidence linking RNA mislocalization to neurodegenerative disease and Alzheimer's-associated synaptic RNA mislocalization, and how it goes a step beyond existing methods. We have also added more detail on how STARIT improves the identification and characterization of cell types and states. The revised text noted by the reviewer is provided below for convenience:

(Introduction, Lines 80-99) "Conventionally, analysis of such imSRT data has relied on assigning and tallying the number of transcripts detected for each gene per segmented cell (Figure 1A). This single-cell gene count matrix can then be used to identify transcriptionally distinct cell-types and states using dimensionality reduction and clustering approaches, analogous to conventional single-cell RNA-sequencing data analyses. However, these conventional analysis approaches inherently neglect the subcellular heterogeneity of these transcripts within cells such as potential nuclear, perinuclear, cytoplasmic, polarized, and other compartment-associated localizations. Such subcellular heterogeneity can play important roles in cellular function, as RNA localization regulates processes including local translation and intracellular signaling [4,5]. Likewise, disruptions in subcellular RNA localization have been linked to disease. For example, previous differential expression analysis of spatially-resolved RNA sequencing data has characterized the composition of synaptic RNAs in Alzheimer's disease, implicating mislocalization of synaptic RNAs within neurons as a feature of a disease-associated cell-state [6]. While imSRT offers transcriptome-wide profiling to map subcellular RNA heterogeneity, computational frameworks capable of systematically incorporating such information remain limited. Considering subcellular heterogeneity in the analysis of imSRT data may enable the identification of functionally

distinct cellular states previously unrecognized through conventional gene count analysis. As an alternative way to represent imSRT data to incorporate subcellular molecular spatial information and address this knowledge gap, we introduce STARIT (Spatial Transcriptomics As Rasterized Image Tensors) (Figure 1B)."

2. **The Introduction also does not include much information about rasterization and any other methods based on image rasterization.**

We thank the reviewer for the suggestion. We have expanded the Introduction to provide additional background on rasterization and its role in STARIT (Introduction, lines 99-115). Specifically, we now describe how transcript coordinates are converted into pixel-based density representations through Gaussian-kernel rasterization to preserve both magnitude and local molecular distributions while enabling compatibility with downstream workflows.

We additionally clarified STARIT relative to prior existing rasterization-based approaches in spatial omics, specifically the cellular-level rasterization strategy from STalign [7] that STARIT extends to the subcellular level, and SEraster [8], a related rasterization preprocessing framework (Introduction, lines 99-109). While image-based and pixel rasterization approaches have been used in spatial omics and imaging analyses, as well as other fields, computational frameworks that specifically transform intracellular transcript coordinates from imSRT data into rasterized tensor representations for downstream cell-state analysis remain limited. We therefore clarify STARIT as an image-based representation framework designed to preserve subcellular transcript organization for downstream analysis. The revised text is provided below for the reviewer's convenience:

(Introduction, Lines 99-115) "STARIT uses a previously developed rasterization strategy to preserve subcellular spatial molecular information by transforming dimensionless transcript coordinate-based measurements into pixel-based images thereby retaining organizational patterns [7]. Briefly, given the x-y coordinates of molecules within a segmented cell, STARIT creates one image per gene by encoding the presence of associated molecules within each image pixel as bits, which are then convolved with a Gaussian blur kernel to produce a smooth image that captures local molecular density. The pixel resolution is defined such that one pixel corresponds to one unit in the input coordinate system (Methods). Such use of rasterization to create image-based representations have previously been explored in spatial omics applications at the cellular level and hence STARIT represents an extension of these similar principles to the subcellular level [7,8]. All rasterized gene images for each cell are combined, resulting in a tensor representation. These tensor representations can then be fed into deep learning computer vision models, such as the pre-trained ResNet101 or UNI model, for feature extraction, resulting in a single cell feature matrix representation that captures the spatial distribution and concentration of molecules within cells. Given this single-cell feature matrix, conventional single-cell dimensionality reduction and clustering analysis pipelines can then be applied to identify clusters of cells with similar features, potentially reflective of distinct subcellular heterogeneity."

3. **The idea of defining cell states based on subcellular heterogeneity should be supported and motivated for. Also, it is not clear what exactly subcellular means, cytoplasm or nucleus only? Should be clarified.**

We thank the reviewer for the feedback. We now explicitly define subcellular heterogeneity in the Introduction as referring broadly to intracellular spatial organization of transcripts, including but not limited to nuclear, perinuclear, cytoplasmic, and polarized/compartment-associated localizations (lines 84-86), and motivate its biological relevance by citing evidence that RNA localization regulates local translation and intracellular signaling and is disrupted in neurodegenerative disease (lines 86-92). We also clarify that STARIT is not restricted to nuclear-versus-perinuclear distinctions. Beyond our primary nuclear/perinuclear simulations, we demonstrate that STARIT also resolves cell states defined by left-right polarized (i.e., non-nuclear/perinuclear) subcellular organization (Supplementary Figure 1; Results 2.1, lines 151-177), directly supporting generalizability to other forms of subcellular heterogeneity (e.g., apical-basal polarity). The revised text is provided below for the reviewer's convenience, along with an accompanying figure (Supplementary Figure 1):

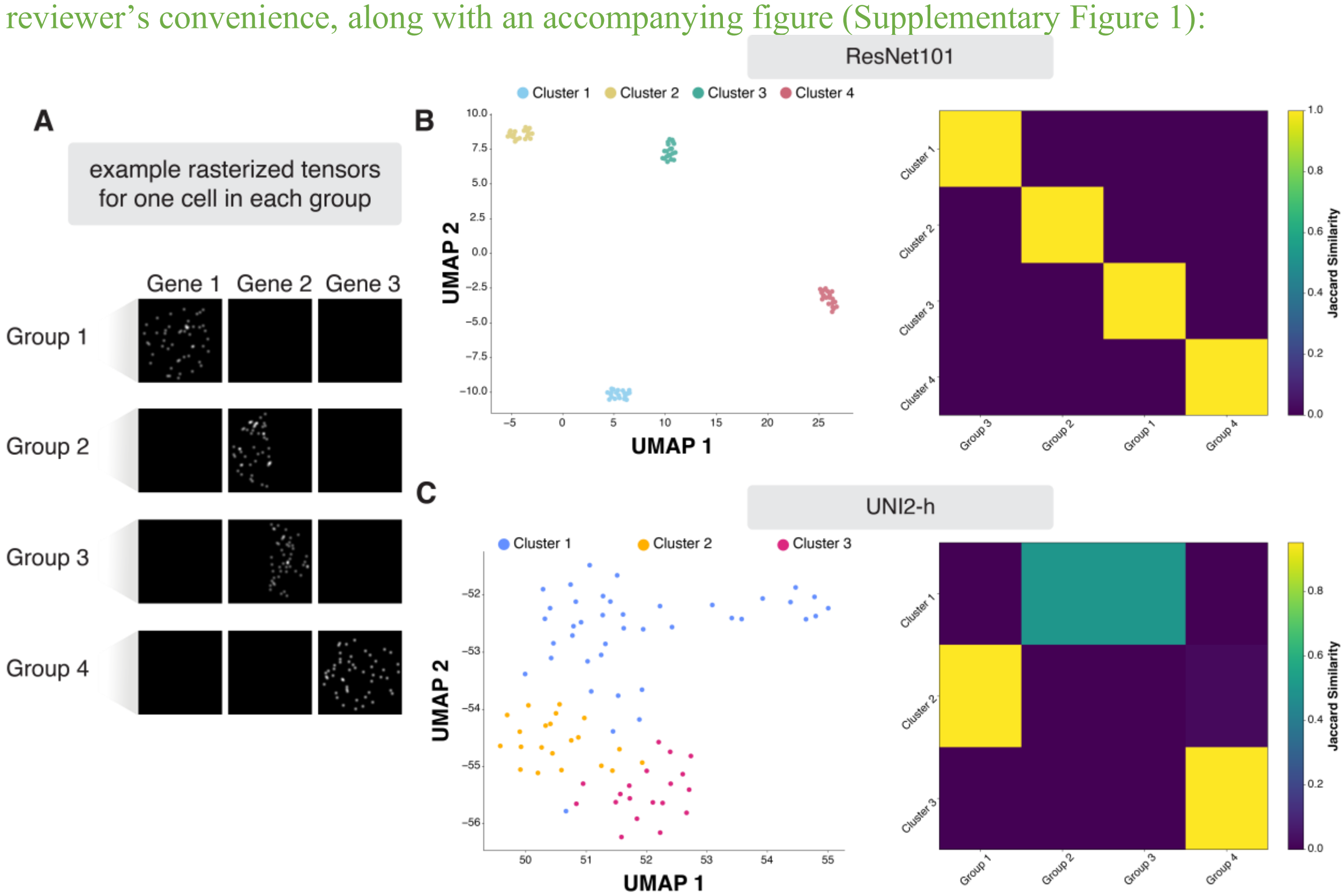

Supplementary Figure 1. Comparison of STARIT-derived ResNet101 features versus STARIT-derived UNI2-h features for left-right polarized localization simulation imSRT dataset. (A) Representative STARIT rasterized image tensors for one cell from each of the four simulated groups. (B) Analysis results of STARIT tensors using ResNet101 feature extraction. Left: UMAP visualization based on STARIT output, embedding colored by Louvain cluster assignment. Right: Jaccard similarity heatmap between ground-truth and STARIT-based Louvain clusters. (C) Analysis results STARIT tensors using UNI2-h feature extraction. Left: UMAP visualization based on STARIT output, embedding colored by

Louvain cluster assignment. Right: Jaccard similarity heatmap between ground-truth and STARIT-based Louvain clusters.

(Introduction, Lines 80-96) "Conventionally, analysis of such imSRT data has relied on assigning and tallying the number of transcripts detected for each gene per segmented cell (Figure 1A). This single-cell gene count matrix can then be used to identify transcriptionally distinct cell-types and states using dimensionality reduction and clustering approaches, analogous to conventional single-cell RNA-sequencing data analyses. However, these conventional analysis approaches inherently neglect the subcellular heterogeneity of these transcripts within cells such as potential nuclear, perinuclear, cytoplasmic, polarized, and other compartment-associated localizations. Such subcellular heterogeneity can play important roles in cellular function, as RNA localization regulates processes including local translation and intracellular signaling [4,5]. Disruptions in subcellular RNA localization have been linked to disease. For example, previous differential expression analysis of spatially-resolved RNA sequencing data has characterized the composition of synaptic RNAs in Alzheimer's disease, implicating mislocalization of synaptic RNAs within neurons as a feature of a disease-associated cell-state [6]. While imSRT offers transcriptome-wide profiling to map subcellular RNA heterogeneity, computational frameworks capable of systematically incorporating such information remain limited. Considering subcellular heterogeneity in the analysis of imSRT data may enable the identification of functionally distinct cellular states previously unrecognized through conventional gene count analysis."

(Results 2.1, Lines 151-177) "Of note, while we used ResNet101, a feature extractor originally developed for natural images, for this analysis, STARIT is compatible with any downstream computer vision model for feature extraction. Although STARIT does not require the use of any specific feature extractor, feature extractor choice can influence which forms of spatial heterogeneity are emphasized. To demonstrate this, we generated an additional simulated dataset in which Groups 1 and 4 again uniquely expressed Gene 1 and Gene 3, respectively, while Groups 2 and 3 uniquely expressed Gene 2, now with left- versus right-polarized intracellular organization (Supplementary Figure 1A, Methods). Applying STARIT with ResNet101, we resolved four clusters corresponding to the four simulated groups, indicating that cell states defined by left- versus right-polarized transcript localization can be resolved (Supplementary Figure 1B). However, when we applied STARIT with UNI2-h, a feature extractor developed from a histopathology foundation model based on a vision transformer (ViT) encoder (ViT-H/14), to the same dataset, the analysis recovered three clusters, with reduced separation of the left- and right-polarized Groups 2 and 3 cells (Supplementary Figure 1C) [9]. Similar behavior was observed for CTransPath, another feature extractor for pathology images. In contrast, additional evaluated ResNet architectures including ResNet18, ResNet50, and ResNet152 more closely matched ResNet101 performance (Supplementary Table 1). This behavior may be expected because CTransPath, for example, is trained using frameworks that incorporate data augmentations such as random rotations and flips in learned visual representations [10]. For UNI2-h, detailed augmentation follows DINOv2 and other self supervised ViT frameworks, which typically apply similar geometric transforms [9]. Because standard ViTs are not rotation-equivariant by design, we hypothesize that any rotational invariance in UNI2-h and related pathology foundation models arise from its pretraining and augmentations rather than the architecture itself. This

result highlights that feature extractor choice may determine whether left- versus right-polarized spatial patterns are preserved or diminished. Such sensitivity may be desirable when left- versus right-polarized spatial patterns is biologically meaningful, in the case of apical-basal organization, but undesirable when orientation reflects technical or arbitrary differences in cell positioning, for example. Thus, feature extractor selection should be guided by the biological question and expected sources of spatial variation in the dataset."

4. **The Methods section is too short and I believe such content from the Supplementary Material should be moved to the main text section.**

We thank the reviewer for the suggestion. We have substantially lengthened and reorganized the Methods section (per PLOS Computational Biology format, Methods now appears after the Discussion), which now includes 16 subsections (4.1-4.16) covering the STARIT rasterization algorithm, image preprocessing and feature extraction, feature weighting, all seven simulated dataset designs, downstream analysis parameters for every dataset, differential feature/expression testing, and the osmFISH and bacterial-MERFISH data processing and analysis pipelines in full detail, moving substantial content previously confined to the Supplementary Material into the main text.

5. **The authors have not described how they have computed the features using ResNet and should explain how ResNet computes them. Converting 4833x33 matrix to 4833x67584 should also be justified by discussing pros and cons and computational cost aspects more.**

We thank the reviewer for the feedback. We have added a dedicated Methods subsection (4.2, "Image Preprocessing and Feature Extraction Workflow", lines 622-634) describing image preprocessing (RGB conversion, aspect-ratio-preserving and padding resize to 224x224, ImageNet normalization) and per-gene feature extraction. Each gene-specific image is passed independently through the pretrained model (with the final classification layer removed) to produce a per-gene embedding (512-D for ResNet18; 2,048-D for ResNet50/101/152; 768-D for CTransPath; 1,536-D for UNI2-h), and per-gene embeddings are concatenated to form one cell-level feature vector. For the osmFISH dataset, this yields the reviewer's example: 33 genes x 2,048 ResNet101 features = 67,584 features per cell, and 4,833 cells x 67,584 features for the full matrix. We refer readers to the original ResNet and foundation-model publications for the internal computation of the embeddings themselves. We discuss the computational cost and interpretability trade-offs of this high-dimensional representation in the Discussion (lines 496-540). The relevant text is provided below for the reviewer's convenience:

(Methods 4.2, Lines 622-634) "For ResNet-based analyses, each gene-specific image was passed independently through the pretrained model. ResNet18 produced a 512-dimensional embedding per gene image, whereas ResNet50, ResNet101, and ResNet152 produced 2,048-dimensional embeddings per gene image. These per-gene embeddings were concatenated across all genes to generate one cell-level feature vector. For example, in simulated datasets with three genes, ResNet101 generated a 6,144-dimensional feature vector per cell. For the osmFISH dataset with 33 genes, ResNet101 generated a 67,584-dimensional feature vector per cell. We also evaluated non-ResNet feature extractors, including CTransPath and UNI2-h. CTransPath produced 768 features per gene image whereas UNI2-h produced 1,536

features per gene image in the simulated analyses, with the resulting feature matrix dimensions reported in Supplementary Table 1. For all feature extractors, the resulting cell-by-feature matrix was used as input for dimensionality reduction, clustering, and visualization."

(Discussion, Lines 496-540) "Feature interpretability also presents an ongoing challenge. As we've demonstrated, STARIT is compatible with any downstream computer vision model for feature extraction, but these models generally function as a "black box" with limited biological interpretability. For example, simply applying ResNet-based feature extraction to one rasterized image per gene for thousands of cells can generate a large feature matrix, with each feature potentially reflecting a mixture of signal related to transcript abundance, spatial spread, local density, shape, orientation, or potential technical artifacts. STARIT currently uses all extracted features for downstream dimensionality reduction and clustering without explicit feature selection before dimensionality reduction. While differential testing can identify features that significantly differ between identified clusters, as we have demonstrated, their biological meaning often remains open to interpretation. Likewise, while explainable AI approaches such as GradCAM and GAINext can provide coarse localization of model attention, they do not explain why specific features drive downstream cluster assignments [28,29]. In the bacterial-MERFISH analysis, supervised metrics helped characterize STARIT-derived clusters, but no single metric fully explained cluster identity. These results suggest that while supervised metrics can improve interpretability, they function as approximations of complex latent image features. We also anticipate that future incorporation of semi-supervised statistics regarding subcellular molecular organization could help further interpret cluster differences [30–32]. The choice of feature extractor is another important consideration. We primarily used ResNet101 as the feature extractor though we also evaluated alternative architectures, including smaller and larger ResNet models as well as domain-specific or foundation-model feature extractors such as CTransPath and UNI2-h (Supplementary Table 1). These comparisons suggest that feature extractor choice can influence downstream clustering and should be selected based on the biological question, data, and expected sources of variation. Computational cost is another practical consideration, particularly for large imSRT datasets. Because STARIT rasterizes and extracts features for every gene independently within each cell, runtime scales primarily with the number of cells and genes assayed. For example, applying STARIT to the full osmFISH dataset (4,833 cells, 33 genes) required approximately 2 hours for rasterization. By contrast, analyzing a targeted subset of the bacterial-MERFISH dataset (463 cells, 1 gene) completed rasterization in under 4 minutes. All reported runtimes reflect serial, non-parallelized execution on the hardware specifications described in Methods (4.16) and are intended to give readers a general sense of expected rasterization runtime. Exact runtimes will vary with computer specifications, parallelization strategy, if any, and dataset size. Downstream feature extraction runtime depends on the chosen computer vision model and its embedding dimensionality rather than on STARIT rasterization itself, and varied accordingly across our analyses: ResNet101 feature extraction required over 2 hours for the full osmFISH dataset and approximately 13 minutes for the bacterial-MERFISH subset, while UNI2-h feature extraction of the bacterial-MERFISH subset required just over 2 minutes As the throughput of imSRT data improves, applying STARIT across all cells and genes in imSRT datasets with hundreds to thousands of targets may become computationally demanding.

Parallelization and use of high-performance computing clusters may alleviate runtime concerns. Alternatively, we suggest a strategy in which conventional gene-count clustering is first used to identify cell types of interest, and STARIT is then selectively applied only to cells and/or gene subsets of interest, for example within a single annotated cell-type, to investigate potential subcellular heterogeneity. This approach can reduce computational cost while retaining STARIT's ability to reveal subcellular heterogeneity within specific populations of interest. "

6. **What are the features extracted that has played a significant role in segregating the genes into 4 different clusters? There should be discussion about that.**

We thank the reviewer for bringing attention to this issue. To identify what features played a significant role in segregating genes into their respective clusters, we performed differential feature testing by applying two-sided Wilcoxon rank-sum tests to extracted image features to identify STARIT-derived features that differed between selected clusters, analogous to differential gene expression analysis (Methods 4.7). This is feasible since we know what features correspond with which gene based on per-gene embeddings from their respective feature extraction model (Methods 4.2). For the simulated data, we performed Wilcoxon rank-sum testing on the extracted STARIT ResNet101 image features between the four identified clusters, identifying significant features associated with the expected gene channels driving each cluster (Figure 1H). For the osmFISH mature oligodendrocyte subclusters, the same approach identified five significantly differential genes' STARIT features (*Anln, Ctps, Gfap, Itpr2, Plp1*; Results 2.3, lines 300-319). Those same five genes were also found to be significantly expressed based on gene count clustering, suggesting STARIT captures comparable transcriptional heterogeneity to conventional gene counts analysis (Results 2.3, lines 320-323). For the standardized bacterial-MERFISH clusters, the same approach identified significant STARIT UNI2-h image features driving each cluster (Supplementary Figure 13). In addition, because raw high-dimensional features are difficult to interpret directly, we additionally derived and validated a set of interpretable supervised metrics (spatial spread, total intensity, normalized expression, cell length, total operon count; Methods 4.15, Supplementary Table 3) that we show correlate with the raw extracted features and that characterize what distinguishes each cluster (Results 2.4, lines 376-409; Figure 3C-F; Supplementary Figures 14). We also agree that feature interpretation remains a challenge and have included this in the Discussion (lines 496-513). The relevant text is provided below for the reviewer's convenience, along with accompanying figures showcasing results:

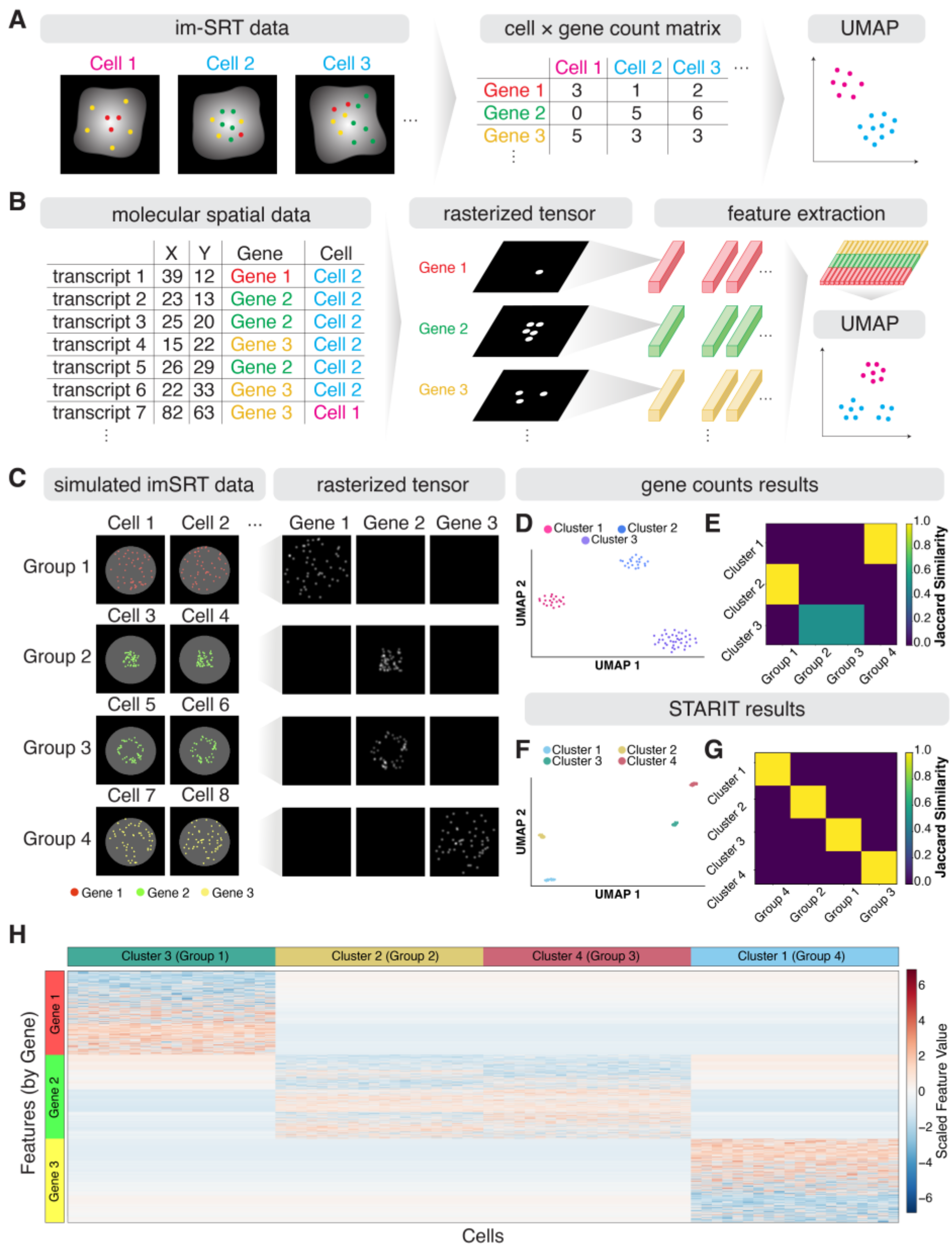

Figure 1. Overview of Spatial Transcriptomics As Rasterized Tensors (STARIT) (A) Schematic of conventional imaging-based spatially resolved transcriptomics (imSRT) data analysis pipeline. Detected transcripts are aggregated into a cell x gene count matrix, which is subsequently used for dimensionality reduction and clustering. Left: Three cells from imSRT data. Middle: Corresponding gene counts matrix. Right: Dimensionality reduction and clustering of imSRT identify distinct cell-types. (B) STARIT (Spatial Transcriptomics As Rasterized Image Tensors) workflow for imSRT data. Left: Molecular representation of imSRT data. Middle: Rasterized image representations for each gene in a cell. Right: Rasterized tensors are then input into a pretrained convolutional neural network (CNN; e.g., ResNet101) for spatial feature extraction. Extracted features are concatenated together to generate a total feature matrix that preserves both molecular and spatial information for downstream clustering and analysis. (C) Simulated imSRT data comprises four groups of cells (Groups 1–4) that exhibit distinct transcriptional and subcellular heterogeneity. Left: Example simulated cells from each group, showing expression of Gene 1 (red), Gene

2 (green), or Gene 3 (yellow). Groups are defined by distinct gene expression (Groups 1 and 4) or distinct subcellular localization patterns of the same gene (nuclear vs. perinuclear; Groups 2 and 3). Right: Rasterized tensor representations of each gene for one simulated cell per group. (D) UMAP visualization based on gene count analysis, with embedding colored by Louvain cluster assignment. (E) Jaccard similarity heatmap comparing ground-truth and gene count-based Louvain clusters. (F) UMAP visualization based on STARIT output, with embedding colored by Louvain cluster assignment. (G) Jaccard similarity heatmap between ground-truth and STARIT-based Louvain clusters. (H) Heatmap of scaled STARIT features grouped by gene channel, shown across the identified clusters. Rows denote extracted features, and columns denote cells.

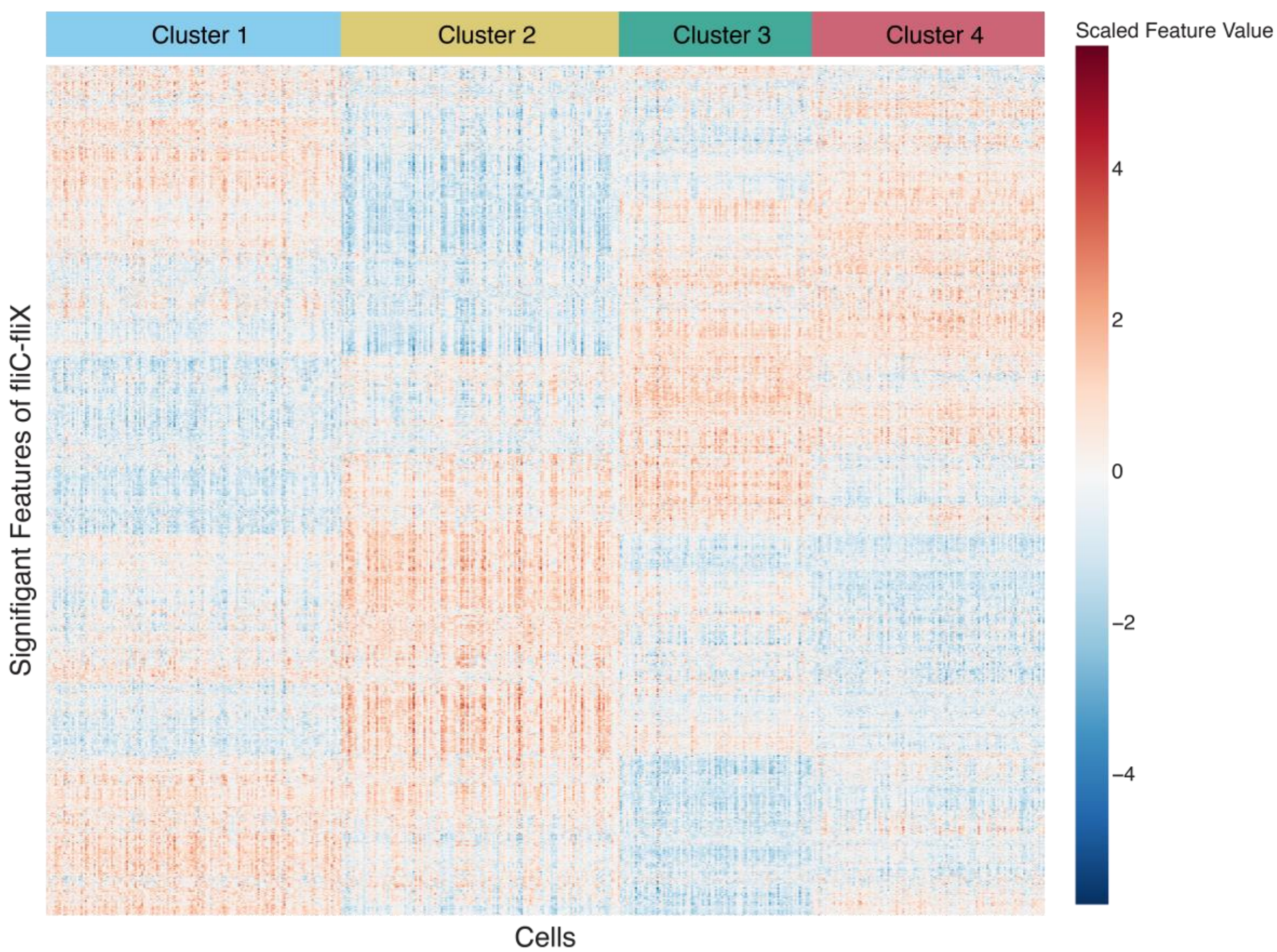

Supplementary Figure 13. Heatmap of significant extracted UNI2-h image features for STARIT-identified clusters in standardized Bacterial-MERFISH *E. coli* data. Heatmap of scaled significant (p-value < 0.001) STARIT features for fliC-fliX, shown across the identified clusters. Rows denote extracted features, and columns denote cells.

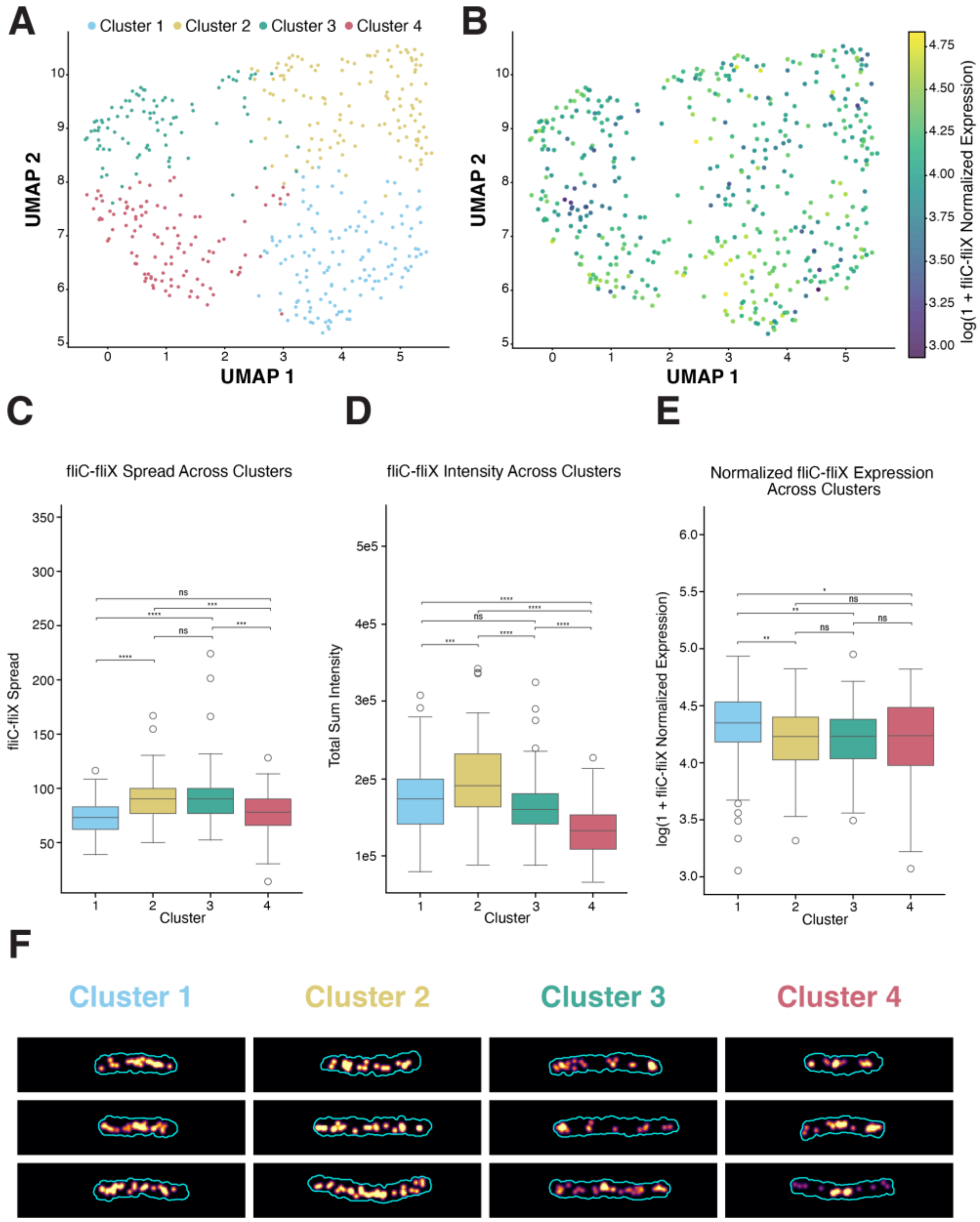

Figure 3. STARIT performance on standardized bacterial-MERFISH 1000X data. (A) Uniform Manifold Approximation and Projection (UMAP) of STARIT-derived UNI2-h features from rotationally standardized 463 *E. coli* cells based fliC-fliX operon localization, imaged with bacterial-MERFISH at 1000X volumetric expansion. (B) UMAP embedding of cells colored by log-transformed fliC-fliX gene expression levels. (C) Distribution of variation in normalized fliC-fliX gene expression across bacterial clusters. Expression levels normalized to total molecular counts per 1000 and log-transformed. Boxplots display median intensity (line), interquartile range (box), and outliers, with colors denoting cluster. Statistical significance determined by Mann-Whitney U test (Bonferroni corrected). (D) Distribution of spatial spread in X of fliC-fliX across bacterial clusters. Boxplots display median intensity (line), interquartile range (box), and outliers, with colors denoting cluster. Statistical significance determined by Mann-Whitney U test (Bonferroni corrected). (E) Distribution of total fliC-fliX signal across bacterial clusters. Boxplots display median intensity (line), interquartile range (box), and outliers, with colors denoting cluster. Statistical significance determined by Mann-Whitney U test (Bonferroni corrected). (F) Select examples of rasterized fliC-fliX transcript distributions for cells in clusters 1-4.

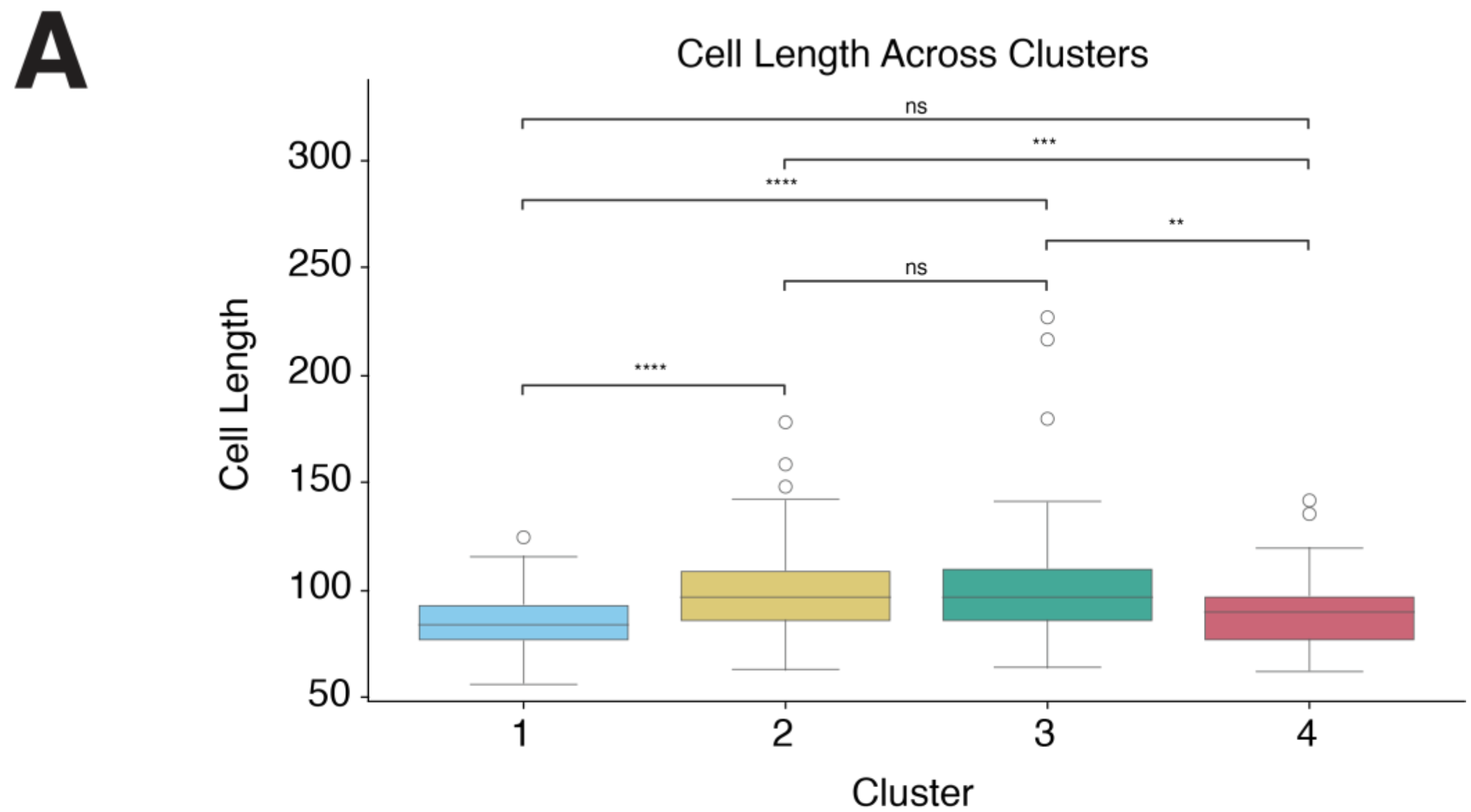

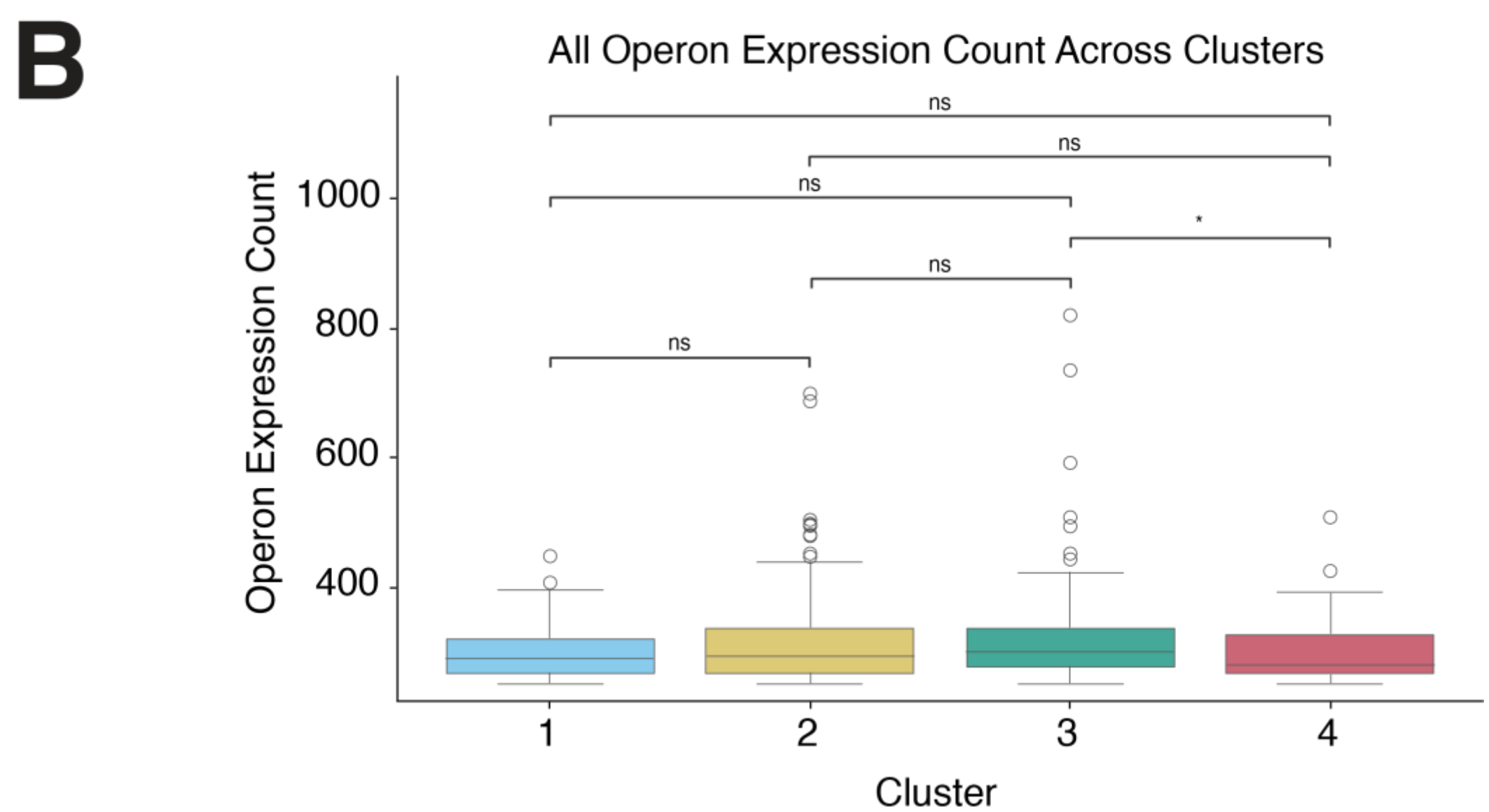

Supplementary Figure 14. Distributions of selected expression and morphology metrics among STARIT-identified bacterial clusters. Additional boxplots showing selected supervised metrics for the four clusters identified from standardized bacterial-MERFISH *E. coli* data. (A) Distribution of total cell length in X across bacterial clusters. Boxplots display median intensity (line), interquartile range (box), and outliers, with colors denoting cluster. Statistical significance determined by Mann-Whitney U test (Bonferroni corrected). (B) Distribution of all operon expression count across bacterial clusters. Boxplots display median intensity (line), interquartile range (box), and outliers, with colors denoting cluster. Statistical significance determined by Mann-Whitney U test (Bonferroni corrected).

(Results 2.3, Lines 300-319) "We do observe some new clusters unique to our STARIT analysis. For example, mature oligodendrocyte cells from the original osmFISH annotation

are now split into clusters 3 and 24. As shown with our simulated data, such clusters may reflect distinct subcellular heterogeneity uniquely distinguishable through our STARIT analysis. We again used the Wilcoxon-rank sum test on the features between these two clusters to identify significantly differential features (Bonferroni-corrected p-value < 0.001) corresponding to the *Anln, Ctps, Gfap, Itpr2,* and *Plp1* genes' STARIT representations. To evaluate whether our newly identified clusters are solely due to STARIT or could be attributable to inherent instability in gene count clustering analysis, we performed normalization, PCA dimensionality reduction, followed by Louvain graph-based and agglomerative clustering to identify 31 clusters from re-clustering on the original gene counts matrix. We were able to observe similar clustering results for the gene counts re-clustering as from our weighted STARIT analysis, reflected in the similar patterns in tissue and UMAP space (Supplementary Figure 8), with a comparable ARI score of 0.342 and on-diagonal Jaccard similarity of 0.295 (Supplementary Figure 8D). Again, these comparable correspondence is consistent across anatomical regions (Supplementary Figure 7).
These clustering results also included a splitting of mature oligodendrocyte cells (Supplementary Figure 8D). Consistent with this, when we used the Wilcoxon-rank sum test on the gene counts between the cells in the mature oligodendrocyte subclusters (cluster 3 and 24) from STARIT, we were able to identify the same genes (*Anln, Ctps, Gfap, Itpr2, Plp1*) as significantly differentially expressed (Bonferroni-corrected p-value < 0.001). "

(Results 2.4, Lines 376-409) "Therefore, to interpret the differences between the clusters identified by STARIT, we applied a supervised approach to extract a set of interpretable magnitude, morphological, and spatial organizational metrics from each bacterium (Methods, Supplementary Table 2). Specifically, we computed each bacterium's normalized fliC-fliX expression magnitude and total operon expression from the original gene counts, the spatial fliC-fliX spread (in x) and cell length from the associated transcript coordinates, and the total fliC-fliX image intensity from STARIT where higher total intensity is associated with a more diffuse spatial distribution while lower total intensity is associated with a more punctated spatial organizational pattern (Methods, Supplementary Table 2). Pairwise two-sided Wilcoxon rank-sum tests on these supervised features revealed distinct combinations of magnitude, morphological, and spatial organizational properties among the 4 clusters (Figure 3C-E; Supplementary Figure 14). For example, Cluster 3 and Cluster 4 do not significantly differ in their normalized fliC-fliX expression magnitude (Figures 3E) and thus likely would not have been distinguished by conventional gene count clustering analysis. Cluster 3 does exhibits significantly greater fliC-fliX total intensity compared to than Cluster 4 but also greater spread, cell length, and total operon count (Figures 3C-D; Supplementary Figure 14A-B). As such, the spatial heterogeneity in fliC-fliX distinguishing these two clusters may be trivially explained by cell size differences. In contrast, Cluster 2 and 3 neither significantly differ in their normalized fliC-fliX expression magnitude, spread, cell length, nor total operon count (Figure 3C, E; Supplementary Figure 14A-B). Yet, they differ significantly in fliC-fliX total intensity, with Cluster 2 cells exhibiting higher total intensities than Cluster 3 cells (Figure 3B). We can thus interpret Cluster 2 cells as having a more diffuse distribution of fliC-fliX transcripts throughout the bacterium and Cluster 3 cells as having a more punctated distribution of fliC-fliX transcripts (Figure 3F). Again, these difference in subcellular spatial organization would be missed by conventional gene count clustering and highlights the ability of STARIT to resolve cells with distinct spatial

heterogeneity. Importantly, we note that these supervised metrics are highly correlated with some STARIT-derived UNIh-2 extracted raw image features (spread maximum Spearman = 0.61; intensity maximum Spearman = 0.64). This correlation suggests that the high-dimensional STARIT-derived features capture these magnitude, morphological, and spatial organizational properties. Still, while fliC-fliX spread and total intensity are significantly different between our 4 identified clusters, these two supervised features do not as clearly delineate all cells across the 4 clusters as well as the UNI2-h raw extracted features (Supplementary Figure 15), suggesting that the bacterial subpopulations may be distinguished by a multivariate combination of features rather than by any two interpretable metrics."

(Discussion, Lines 496-513) "Feature interpretability also presents an ongoing challenge. As we've demonstrated, STARIT is compatible with any downstream computer vision model for feature extraction, but these models generally function as a "black box" with limited biological interpretability. For example, simply applying ResNet-based feature extraction to one rasterized image per gene for thousands of cells can generate a large feature matrix, with each feature potentially reflecting a mixture of signal related to transcript abundance, spatial spread, local density, shape, orientation, or potential technical artifacts. STARIT currently uses all extracted features for downstream dimensionality reduction and clustering without explicit feature selection before dimensionality reduction. While differential testing can identify features that significantly differ between identified clusters, as we have demonstrated, their biological meaning often remains open to interpretation. Likewise, while explainable AI approaches such as GradCAM and GAINext can provide coarse localization of model attention, they do not explain why specific features drive downstream cluster assignments [28,29]. In the bacterial-MERFISH analysis, supervised metrics helped characterize STARIT-derived clusters, but no single metric fully explained cluster identity. These results suggest that while supervised metrics can improve interpretability, they function as approximations of complex latent image features. We also anticipate that future incorporation of semi-supervised statistics regarding subcellular molecular organization could help further interpret cluster differences [30–32]."

7. **What matrix has been used to identify important features and on what basis low important ones were dropped? What was the data matrix before and after?**

We thank the reviewer for the question. To clarify, all extracted features are used for dimensionality reduction and clustering. The feature matrix for each dataset is reported in Supplementary Table 1. STARIT does not perform any feature selection or dropping of any features prior to PCA. We have now made the absence of feature selection explicit in the Discussion (lines 496-504) and note that identifying which features are most informative prior to clustering remains an open challenge. The relevant text is provided below for the reviewer's convenience:

(Discussion, Lines 496-504) "Feature interpretability also presents an ongoing challenge. As we've demonstrated, STARIT is compatible with any downstream computer vision model for feature extraction, but these models generally function as a "black box" with limited

biological interpretability. For example, simply applying ResNet-based feature extraction to one rasterized image per gene for thousands of cells can generate a large feature matrix, with each feature potentially reflecting a mixture of signal related to transcript abundance, spatial spread, local density, shape, orientation, or potential technical artifacts. STARIT currently uses all extracted features for downstream dimensionality reduction and clustering without explicit feature selection before dimensionality reduction."

8. The computation times are missing and should be tested + reported especially for large datasets.

We thank the reviewer for the great suggestion. We address this directly in a new Discussion section (lines 519-540) reporting rasterization runtimes for our two largest, real (non-simulated) datasets to help readers gauge runtime expectations. We note the exact runtimes for even the same dataset will vary depending on the computer specifications, use of parallelization if any, etc. Processing the full osmFISH dataset (4,833 cells, 33 genes = 159,489 rasterized images) required approximately 2 hours for rasterization. The subsetted bacterial-MERFISH dataset (463 cells, 1 gene = 463 rasterized images) completed rasterization in under 4 minutes. Downstream feature extraction runtime depended on the chosen computer vision model: ResNet101 feature extraction required over 2 hours for the full osmFISH dataset and approximately 13 minutes for the bacterial-MERFISH subset, while UNI2-h feature extraction of the bacterial-MERFISH subset required just over 2 minutes. We note that runtime scales primarily with the number of cell-gene image pairs processed, and, motivated by this, we recommend a hierarchical workflow for large imSRT datasets: conventional gene-count clustering to identify cells and genes of interest followed by the application of STARIT selectively to certain cell types or gene subsets rather than uniformly across all cells and genes. The relevant text is provided below for the reviewer's convenience:

(Discussion, Lines 519-540) "Computational cost is another practical consideration, particularly for large imSRT datasets. Because STARIT rasterizes and extracts features for every gene independently within each cell, runtime scales primarily with the number of cells and genes assayed. For example, applying STARIT to the full osmFISH dataset (4,833 cells, 33 genes) required approximately 2 hours for rasterization. By contrast, analyzing a targeted subset of the bacterial-MERFISH dataset (463 cells, 1 gene) completed rasterization in under 4 minutes. All reported runtimes reflect serial, non-parallelized execution on the hardware specifications described in Methods (4.16) and are intended to give readers a general sense of expected rasterization runtime. Exact runtimes will vary with computer specifications, parallelization strategy, if any, and dataset size. Downstream feature extraction runtime depends on the chosen computer vision model and its embedding dimensionality rather than on STARIT rasterization itself, and varied accordingly across our analyses: ResNet101 feature extraction required over 2 hours for the full osmFISH dataset and approximately 13 minutes for the bacterial-MERFISH subset, while UNI2-h feature extraction of the bacterial-MERFISH subset required just over 2 minutes As the throughput of imSRT data improves, applying STARIT across all cells and genes in imSRT datasets with hundreds to thousands of targets may become computationally demanding. Parallelization and use of high-performance computing clusters may alleviate runtime concerns. Alternatively, we suggest a strategy in which conventional gene-count clustering is first used to identify cell types of interest, and

STARIT is then selectively applied only to cells and/or gene subsets of interest, for example within a single annotated cell-type, to investigate potential subcellular heterogeneity. This approach can reduce computational cost while retaining STARIT's ability to reveal subcellular heterogeneity within specific populations of interest. "

Minor Issues:

1. **Overall, the manuscript lacks proper flow and does not clearly explain the methods and findings.**

We thank the reviewer for the feedback. We have substantially revised the manuscript throughout—expanding the Introduction, Methods, Results, and Discussion, adding dataset and configuration summary tables as well as other Supplementary Material, and reorganizing the Methods—to improve overall flow, methodological transparency, and clarity. We have added more detailed explanations on the methods and findings and have included stronger justification of modeling choices and clearer dataset summaries to enhance both readability and rigor.

2. **The abstract structure should match the journal requirements.**

We thank the reviewer and have fixed this in the revision, the main text matching the journal requirements and structure.

3. **There are too many long sentences, e.g., lines 221-228 or 240-241.**

We thank the reviewer for this observation. We have revised the manuscript to improve conciseness and readability by shortening several long sentences, including those noted by the reviewer. The revised text noted by the reviewer is provided below for convenience:

(Results 2.3, Lines 319-326) "These results suggest that STARIT identified new clusters that split annotated mature oligodendrocytes, potentially reflecting distinct cell states. However, re-clustering of gene counts could produce similar results, potentially indicating how subcellular localization changes often accompany magnitude changes. Nonetheless, STARIT captures comparable aspects of transcriptional heterogeneity as conventional gene count analysis. Furthermore, analysis of STARIT-derived image features identified genes driving cluster differences comparable to differential expression with gene count clustering analysis."

(Results 2.4, Lines 343-345) "Notably, these clusters of bacteria are not obviously distinguished based on fliC-fliX expression magnitude (Supplementary Figure 9B). Therefore, they would not have been readily found via conventional count-based analysis ."

4. **The Jupyter notebooks on GitHub seem to work but some of the packages required (git, pandas, torchvision, scikit-learn, community_louvain) could be mentioned before for installation.**

We thank the reviewer for the suggestion. We have added descriptions of the required packages for running the Jupyter notebook tutorials to the STARIT GitHub repository and updated the installation instructions accordingly. Several screenshots are provided below for the reviewer's reference:

Preview | Code | Blame 3154 lines (3154 loc) · 1.12 MB Raw

STARIT Tutorial: Nuclear–Perinuclear Localization-Marker Simulation Dataset

Dataset 1; Supplementary Table 1

This tutorial demonstrates how STARIT converts simulated image-based spatial transcriptomics (imSRT) molecular *x,y* coordinates into gene-specific raster images and how those images retain localization information during downstream analysis. The simulation dataset contains four known groups that differ in gene transcript abundance, gene spatial localization/organization, or both, without any added sparse-gene count noise. More information on STARIT and this datatset can be found in the paper.

Learning Objectives

By the end of the notebook, you will be able to:

1. rasterize transcript coordinates using STARIT
2. extract and concatenate gene-level ResNet-101 image features
3. compare unweighted and expression-weighted image representations
4. cluster cells with a k-nearest-neighbor graph and Louvain clustering and
5. compare image-based results with raw and scaled gene-count clustering

Input Files

- `data/simulated_nuclear_peri/simulation_nuclear_peri_dataset.csv`: one transcript per row, with `x_position`, `y_position`, `gene_id`, `cell_id`, and `group_id`.
- `data/simulated_nuclear_peri/simulation_nuclear_peri_dataset_count_matrix.csv`: one cell per row, with its group and counts for `gene1`, `gene2`, and `gene3`.

Run the notebook from the repository root so these relative paths resolve correctly. The workflow is: environment setup → data inspection → STARIT rasterization → image-feature analysis → gene-counts analysis.

1. Reproducible Environment and Installation

The STARIT package itself requires only NumPy and Matplotlib. This tutorial also uses tabular-data, image-model, dimensionality-reduction, graph-clustering, and notebook packages for the downstream analysis. The versions below were read from the Anaconda `base` kernel in which these notebooks were developed.

Software	Version in the tutorial kernel	Role in this notebook	Installation name
Python	3.11.7	Runtime	`python`
Git	2.50.1 (Apple Git-155)	Obtain and version the repository; not imported by Python	operating-system package
pip	25.3	Install Python packages	`pip`
STARIT	0.1.0	Rasterize spatial transcript coordinates	local project (`pip install -e .`)
NumPy	1.26.4	Arrays and numerical operations	`numpy`
Matplotlib	3.8.0	Image creation and plotting	`matplotlib`
pandas	2.1.4	Read and transform tabular data	`pandas`
Pillow	10.2.0	Load and pad images (`PIL`)	`pillow`
SciPy	1.11.4	Hungarian assignment	`scipy`
scikit-learn	1.2.2	PCA, t-SNE, nearest-neighbor graphs, and metrics	`scikit-learn`
NetworkX	3.1	Graph representation	`networkx`
python-louvain	0.16	Louvain community detection (`community.community_louvain`)	`python-louvain`
PyTorch	2.7.1	ResNet-101 inference	`torch`
TorchVision	0.22.1	Pretrained ResNet-101 and image transforms	`torchvision`
UMAP	0.5.6	Nonlinear two-dimensional embedding	`umap-learn`
natsort	8.4.0	Reproducible natural ordering of cell and gene names	`natsort`
JupyterLab	4.0.11	Notebook interface	`jupyterlab`
Notebook	7.0.8	Notebook document support	`notebook`
IPython kernel	6.28.0	Execute Python cells	`ipykernel`

Git is a system tool rather than a Python dependency:

- **Windows:** Download and run the installer from git-scm.com or run `winget install --id Git.Git` in Command Prompt / PowerShell.
- **macOS:** Installing the Xcode Command Line Tools provides Git (`xcode-select --install`).
- **Debian/Ubuntu:** Use `sudo apt-get install git` .

Confirm the installation in your terminal with `git --version` .

To reproduce the Python environment with Conda, run the following commands in a terminal. Skip `git clone` if you already have the repository.

```
git clone https://github.com/JEFworks-Lab/STARIT.git
cd STARIT

conda create --name starit-tutorials python=3.11.7 pip=25.3
conda activate starit-tutorials

python -m pip install \
  numpy==1.26.4 matplotlib==3.8.0 pandas==2.1.4 pillow==10.2.0 \
  scipy==1.11.4 scikit-learn==1.2.2 networkx==3.1 \
  python-louvain==0.16 torch==2.7.1 torchvision==0.22.1 \
  umap-learn==0.5.6 natsort==8.4.0 \
  jupyterlab==4.0.11 notebook==7.0.8 ipykernel==6.28.0
python -m pip install -e .

python -m ipykernel install --user \
  --name starit-tutorials \
  --display-name "Python 3.11.7 (STARIT tutorials)"
jupyter lab
```

Select **Python 3.11.7 (STARIT tutorials)** as the notebook kernel, then restart the kernel after installation. The first execution of the ResNet101 cell may download pretrained weights (approximately 171 MB) if they are not already cached.

If you are running this notebook in **Google Colab** or a standalone **Jupyter Notebook**, execute the cell below to clone the repository, install the exact package versions, and set up STARIT in editable mode.

> **Note for Google Colab Users:** Google Colab comes with git pre-installed. Once the dependencies install, you may need to restart the runtime (**Runtime > Restart session**) for updated package versions to take effect.

In [1]:

```
# 1. Clone the repository (skip if running locally from within the cloned repository)
#!git clone https://github.com/JEFworks-Lab/STARIT.git
#%cd STARIT

# 2. Install required dependencies with exact pinned versions
"""
!python -m pip install \
  numpy==1.26.4 \
  matplotlib==3.8.0 \
  pandas==2.1.4 \
  pillow==10.2.0 \
  scipy==1.11.4 \
  scikit-learn==1.2.2 \
  networkx==3.1 \
  python-louvain==0.16 \
  torch==2.7.1 \
  torchvision==0.22.1 \
  umap-learn==0.5.6 \
  natsort==8.4.0
  """

# 3. Install the local STARIT package
#!python -m pip install -e ./*
```

Out[1]:

```
'\n!python -m pip install   numpy==1.26.4   matplotlib==3.8.0   pandas==2.1.4   pillow==10.2.0   scipy==1.11.4   scikit-learn==1.2.2   networkx==3.1   pytho
n-louvain==0.16   torch==2.7.1   torchvision==0.22.1   umap-learn==0.5.6   natsort==8.4.0\n  '
```

2. Import Packages and Load the Simulation Dataset

First, we import the required packages to run this tutorial and fix the NumPy and PyTorch random seeds. The fixed seed improves repeatability for stochastic dimensionality-reduction and clustering steps. We also load both the simulated data and count matrix.

```
# Standard-library utilities
import os
import re

# Data handling and visualization
import matplotlib.pyplot as plt
import networkx as nx
import numpy as np
import pandas as pd
from PIL import Image

# STARIT rasterization
from starit import get_bounding_box, save_image, starit

# Feature extraction, embedding, clustering, and evaluation
import community.community_louvain as cl
import umap
from natsort import natsorted
from scipy.optimize import linear_sum_assignment
from sklearn.decomposition import PCA
from sklearn.manifold import TSNE
from sklearn.metrics import adjusted_rand_score, jaccard_score
from sklearn.neighbors import kneighbors_graph
from sklearn.preprocessing import LabelEncoder
import torch
from torchvision import models, transforms

# Fix stochastic components so repeated runs are comparable.
RANDOM_SEED = 42
np.random.seed(RANDOM_SEED)
torch.manual_seed(RANDOM_SEED)

# Select and load the noise-free simulation.
sim_data_folder = "simulated_nuclear_peri"

print("Loading dataset without noise.")
dataset = pd.read_csv(
    f"data/{sim_data_folder}/simulation_nuclear_peri_dataset.csv"
)
counts_matrix = pd.read_csv(
```

5. The Supplementary Figures seem to be replicates of the main figures.

We thank the reviewer for the feedback. Many of the supplementary figures are very similar to the main figures due to being the same embeddings but colored by different annotations, for example. We have updated the Supplementary Figures in the Supplementary Material revision to better emphasize the differences in respective titles. We have also written fully detailed, self-contained captions for all 15 Supplementary Figures so that each figure's distinct purpose is clear without needing to cross-reference the main text.
