# Supplementary material for: Spatial Transcriptomics As Rasterized Image Tensors (STARIT) characterizes cell states with subcellular molecular heterogeneity
Source: PLoS Comput Biol. 2026 Oct 6;22(10):e1014845. doi: 10.1371/journal.pcbi.1014845 (PMC13657327; doi:10.1371/journal.pcbi.1014845)
Supplement: Supplementary file 2 [file pcbi.1014845.s019.docx]

In this work, Velazquez et al introduce STARIT, a workflow for cell type assignment and clustering from imaging based spatial transcriptomics data. They demonstrate the power of STARIT using simulated data and real world MERFISH data. The major contribution of STARIT is that it is able to capture cell types and states defined by differences in the spatial arrangement of molecules within cells (for example cells with distinct nuclear vs perinuclear molecular localization). Overall, I think the method is a useful contribution to the field and provide some specific comments below:

Spatial transcriptomics methods are often applied to 4-5 micron thick tissue sections. In any given section there are fully intact cells, as well as majority and minority intact cells where only part of the nucleus and cytoplasm are captured. STARIT, a method that uses spatial localization of molecules to define cell types and states, may be particularly sensitive to this problem. I suggest the authors conduct another simulated data experiment where they randomly cut pieces from cells and determine how well STARIT can still correctly call cell types. How does the performance of STARIT on this data compare to standard methods?

How well does STARIT apply to diseased tissues where cell states are not discrete but exist along a continuum? For example, cancer cells treated with chemotherapy may possess a heterogeneous mix of responding and resistant characteristics. How can this be captured using the STARIT method?

The authors demonstrated their method on simulated data as well as two FISH data sets. Can they demonstrate the applicability of their software on another imSRT dataset such as Xenium and comment on how the performance is similar or different to MERFISH?
