# Supplementary material for: Spatial Transcriptomics As Rasterized Image Tensors (STARIT) characterizes cell states with subcellular molecular heterogeneity
Source: PLoS Comput Biol. 2026 Oct 6;22(10):e1014845. doi: 10.1371/journal.pcbi.1014845 (PMC13657327; doi:10.1371/journal.pcbi.1014845)
Supplement: Supplementary file 3 [file pcbi.1014845.s020.docx]

The authors have developed the STARIT framework to identify cell states using subcellular molecular information derived from imSRT data. The framework converts transcripts within segmented cells in imaging data into a tensor representation that can be combined with deep learning computer vision models for downstream analysis. It provides deeper insights into subcellular heterogeneity and improves the identification and characterization of cell types and states that may be overlooked when using only gene-count representations.

**Major issues:**

- The Introduction lacks why authors have developed STARIT and how it is better than already existing methods. The section needs more reasoning and background on the existing tools and their limitations.
- The Introduction also does not include much information about rasterization and any other methods based on image rasterization.
- The idea of defining cell states based on subcellular heterogeneity should be supported and motivated for. Also, it is not clear what exactly subcellular means, cytoplasm or nucleus only? Should be clarified.
- The Methods section is too short and I believe such content from the Supplementary Material should be moved to the main text section.
- The authors have not described how they have computed the features using ResNet and should explain how ResNet computes them. Converting 4833x33 matrix to 4833x67584 should also be justified by discussing pros and cons and computational cost aspects more.
- What are the features extracted that has played a significant role in segregating the genes into 4 different clusters? There should be discussion about that.
- What matrix has been used to identify important features and on what basis low important ones were dropped? What was the data matrix before and after?
- The computation times are missing and should be tested + reported especially for large datasets.

**Minor issues:**

- Overall, the manuscript lacks the proper flow and does not clearly explain the methods and findings.
- The abstract structure should match the journal requirements.
- There are too many long sentences, e.g., lines 221-228 or 240-241.
- The Jupyter notebooks on GitHub seem to work but some of the packages required (git, pandas, torchvision, scikit-learn, community_louvain) could be mentioned before for installation.
- The Supplementary Figures seem to be replicates of the main figures.
