# Supplementary material for: Spatial Transcriptomics As Rasterized Image Tensors (STARIT) characterizes cell states with subcellular molecular heterogeneity
Source: PLoS Comput Biol. 2026 Oct 6;22(10):e1014845. doi: 10.1371/journal.pcbi.1014845 (PMC13657327; doi:10.1371/journal.pcbi.1014845)
Supplement: Peer review history

# Peer review: Decision Letter 0

**DOI:** 10.1371/journal.pcbi.1014845.r001

**Authors:** Ferhat Ay, Virginie Uhlmann

31 Mar 2026

PCOMPBIOL-D-26-00215

Spatial Transcriptomics As Rasterized Image Tensors (STARIT) characterizes cell states with subcellular molecular heterogeneity

PLOS Computational Biology

Dear Dr. Fan,

Thank you for submitting your manuscript to PLOS Computational Biology. After careful consideration, we feel that it has merit but does not fully meet PLOS Computational Biology's publication criteria as it currently stands. Therefore, we invite you to submit a revised version of the manuscript that addresses the points raised during the review process.

Please submit your revised manuscript by May 31 2026 11:59PM. If you will need more time than this to complete your revisions, please reply to this message or contact the journal office at ploscompbiol@plos.org. When you're ready to submit your revision, log on to https://www.editorialmanager.com/pcompbiol/ and select the 'Submissions Needing Revision' folder to locate your manuscript file.

Please include the following items when submitting your revised manuscript:

* A letter that responds to each point raised by the editor and reviewer(s). You should upload this letter as a separate file labeled 'Response to Reviewers'. This file does not need to include responses to formatting updates and technical items listed in the 'Journal Requirements' section below.

* A marked-up copy of your manuscript that highlights changes made to the original version. You should upload this as a separate file labeled 'Revised Manuscript with Track Changes'.

* An unmarked version of your revised paper without tracked changes. You should upload this as a separate file labeled 'Manuscript'.

If you would like to make changes to your financial disclosure, competing interests statement, or data availability statement, please make these updates within the submission form at the time of resubmission. Guidelines for resubmitting your figure files are available below the reviewer comments at the end of this letter

We look forward to receiving your revised manuscript.

Kind regards,

Virginie Uhlmann

Academic Editor

PLOS Computational Biology

Ferhat Ay

Section Editor

PLOS Computational Biology

**Journal Requirements:**

1) Please ensure that the CRediT author contributions listed for every co-author are completed accurately and in full.

At this stage, the following Authors/Authors require contributions: Caleb Hallinan, Roujin An, Kalen Clifton, Dee Velazquez, and Jean Fan. Please ensure that the full contributions of each author are acknowledged in the "Add/Edit/Remove Authors" section of our submission form.

The list of CRediT author contributions may be found here: https://journals.plos.org/ploscompbiol/s/authorship#loc-author-contributions

2) We ask that a manuscript source file is provided at Revision. Please upload your manuscript file as a .doc, .docx, .rtf or .tex. If you are providing a .tex file, please upload it under the item type u2018LaTeX Source Fileu2019 and leave your .pdf version as the item type u2018Manuscriptu2019.

3) Please provide an Author Summary. This should appear in your manuscript between the Abstract (if applicable) and the Introduction, and should be 150-200 words long. The aim should be to make your findings accessible to a wide audience that includes both scientists and non-scientists. Sample summaries can be found on our website under Submission Guidelines:

https://journals.plos.org/ploscompbiol/s/submission-guidelines#loc-parts-of-a-submission

4) Please upload all main figures as separate Figure files in .tif or .eps format. For more information about how to convert and format your figure files please see our guidelines:

https://journals.plos.org/ploscompbiol/s/figures

5) We have noticed that you have uploaded Supporting Information files, but you have not included a list of legends. Please add a full list of legends for your Supporting Information files after the references list.

6) Please amend your detailed Financial Disclosure statement. This is published with the article. It must therefore be completed in full sentences and contain the exact wording you wish to be published.

1) State the initials, alongside each funding source, of each author to receive each grant. For example: "This work was supported by the National Institutes of Health (####### to AM; ###### to CJ) and the National Science Foundation (###### to AM)."

2) State what role the funders took in the study. If the funders had no role in your study, please state: "The funders had no role in study design, data collection and analysis, decision to publish, or preparation of the manuscript.".

Note: If the reviewer comments include a recommendation to cite specific previously published works, please review and evaluate these publications to determine whether they are relevant and should be cited. There is no requirement to cite these works unless the editor has indicated otherwise.

**Reviewers' comments:**

Reviewer's Responses to Questions

**Comments to the Authors:**

**Please note that two reviews are uploaded as attachments.**

Reviewer #1: The manuscript presents a novel and well-implemented framework (STARIT) for incorporating subcellular transcript distribution into spatial transcriptomics analysis. The ability to differentiate between nuclear and perinuclear transcript localization is a significant advancement over traditional count-based representations. The overall presentation is clear, and the workflow is understandable.

However, while the core idea is compelling, the manuscript would benefit from greater methodological transparency and clearer experimental organization. Specifically, stronger justification of modeling choices, clearer dataset summaries would enhance both readability and rigor.

First, a concise summary table for each dataset would be helpful. Currently, the dataset number, clustering parameters, and method performance across the simulated, noisy simulated, osmFISH, and bacterial-MERFISH datasets are described throughout the text, making it difficult for readers to compare results across conditions. A structured summary would improve clarity and transparency.

Second, the choice of ResNet101 as the feature extractor needs clearer justification. ResNet101 is an older architecture trained on natural images, and it is unclear why this model was chosen over alternatives.

Reviewer #2: In this manuscript, the authors investigated the use of a computer vision model, ResNet101, to help identify cell-states based on imaging-based spatially resolved transcriptonomics (imSRT). They identify a crucial limitation of the current single-cell gene count based methods which fail to take subcellular heterogeneity into account. They show excellent results on synthetic data but when applying their approach to real imSRT data, the results are more mixed and it is not clear they provide an improvement over existing methods. This work contributes to the field by presenting original research in a valuable area of study. However, revisions are necessary before publication.

Major comments:

1. Based on results, it is not clear to that the approach provides any practical advantage on real world data over existing gene-count approaches. When comparing Supplementary Fig. 5C with Fig. 2H, it is not clear which result is better. The authors present quantitative results for their approach (ARI and on-diagonal Jaccard similarities) on lines 204-205. However, they do not present the same for the existing approaches. I suggest adding the direct quantitative comparison of the approaches. If other comparable non-gene-count approaches exist they should also be included in this quantitative comparison if possible.

2. I am not convinced that ResNet101 is well suited for this task for a variety of reasons.

a. CNNs are known to struggle with sparse data which seems to be the case here (Fig. 1C).

b. With a learned feature vector for each gene, the resulting feature vector is very large (>67,000 here) and uninterpretable. It is unclear how much of the information in this large feature vector is meaningful rather than artifacts of the rasterization or other factors.

c. CNNs are not invariant to orientation or scale.

d. ResNet101 is very large and perhaps a smaller version like ResNet18 (or even simpler CNNs) might provide similar results at reduced computational cost and potentially less overfitting.

Based on these factors, perhaps a graph neural network or point cloud based approach makes more sense. Another idea would be to combine the gene-count data with the ResNet101 feature vector to ensure you are not losing important gene-count information that ResNet101 may not pick up effectively. I do not think it is necessary to run additional calculations but additional motivation for ResNet101 over alternatives would be beneficial.

3a. In section 3.4 you discuss the ability of STARIT to cluster E. coli cells based on the orientation of fliC-fliX molecules. However, given that CNNs are not rotationally invariant, it would be a surprising result if ResNet101 was unable to separate cells with different orientation. Given that the orientation was entirely and trivially driven by the cell orientation, this does not seem like a meaningful result. It would be easy to develop a method to classify cells by orientation without the use of a large DL model like ResNet101. I suggest the authors add further justification/discussion as to why this result is meaningful. Otherwise it just seems to highlight one of the biggest weaknesses of the approach (see 3b below).

3b. Building off 3a above, the fact that a trivial pattern (rotation of cells) can have such a large impact on final clusters is a reason for serious concern and highlights one key limitation of CNNs here. The authors have acknowledged this limitation in their discussion (lines 289-297). As an area of future research, I suggest addressing this issue by augmenting the data (random rotations for example) and then finetuning the model using a contrastive loss to encourage feature vectors for the same cell to be similar. In theory, this could help the approach be more invariant to rotation without any change in overall approach. Alternatively, this could be partially achieved without retraining by passing several augmented inputs for each gene and aggregating the feature vectors (possibly average or max pooling). This is not a comment that needs to be directly addressed, but a suggestion to the authors and something potentially worth mentioning in future directions.

Minor comments:

4. The terminology of "Cluster 1-1", "Cluster 1-0", etc. is not really clear. It seems that the first number denotes gene-count (1) vs STARIT (2) and the second number denotes the cluster index. Perhaps use something like Cluster_GC-1 for the first gene-count cluster would make things more clear. If you keep the current labeling, I suggesting making it more clear. It also seems strange that the approach indexing starts at 1 while the cluster indexing starts at 0.

Reviewer #3: In this work, Velazquez et al introduce STARIT, a workflow for cell type assignment and clustering from imaging based spatial transcriptomics data. They demonstrate the power of STARIT using simulated data and real world MERFISH data. The major contribution of STARIT is that it is able to capture cell types and states defined by differences in the spatial arrangement of molecules within cells (for example cells with distinct nuclear vs perinuclear molecular localization). Overall, I think the method is a useful contribution to the field and provide some specific comments below:

Spatial transcriptomics methods are often applied to 4-5 micron thick tissue sections. In any given section there are fully intact cells, as well as majority and minority intact cells where only part of the nucleus and cytoplasm are captured. STARIT, a method that uses spatial localization of molecules to define cell types and states, may be particularly sensitive to this problem. I suggest the authors conduct another simulated data experiment where they randomly cut pieces from cells and determine how well STARIT can still correctly call cell types. How does the performance of STARIT on this data compare to standard methods?

How well does STARIT apply to diseased tissues where cell states are not discrete but exist along a continuum? For example, cancer cells treated with chemotherapy may possess a heterogeneous mix of responding and resistant characteristics. How can this be captured using the STARIT method?

The authors demonstrated their method on simulated data as well as two FISH data sets. Can they demonstrate the applicability of their software on another imSRT dataset such as Xenium and comment on how the performance is similar or different to MERFISH?

Reviewer #4: Please see the attachment.

**********

**Have the authors made all data and (if applicable) computational code underlying the findings in their manuscript fully available?**

The PLOS Data policyrequires authors to make all data and code underlying the findings described in their manuscript fully available without restriction, with rare exception (please refer to the Data Availability Statement in the manuscript PDF file). The data and code should be provided as part of the manuscript or its supporting information, or deposited to a public repository. For example, in addition to summary statistics, the data points behind means, medians and variance measures should be available. If there are restrictions on publicly sharing data or code —e.g. participant privacy or use of data from a third party—those must be specified.

Reviewer #1: Yes

Reviewer #2: Yes

Reviewer #3: Yes

Reviewer #4: Yes

**********

PLOS authors have the option to publish the peer review history of their article (what does this mean?). If published, this will include your full peer review and any attached files.

If you choose “no”, your identity will remain anonymous but your review may still be made public.

**Do you want your identity to be public for this peer review?** For information about this choice, including consent withdrawal, please see our Privacy Policy.

Reviewer #1: No

Reviewer #2: No

Reviewer #3: No

Reviewer #4: No

[NOTE: If reviewer comments were submitted as an attachment file, they will be attached to this email and accessible via the submission site. Please log into your account, locate the manuscript record, and check for the action link "View Attachments". If this link does not appear, there are no attachment files.]

**Figure resubmission:**

While revising your submission, we strongly recommend that you use PLOS’s NAAS tool (https://ngplosjournals.pagemajik.ai/artanalysis) to test your figure files. NAAS can convert your figure files to the TIFF file type and meet basic requirements (such as print size, resolution), or provide you with a report on issues that do not meet our requirements and that NAAS cannot fix.

After uploading your figures to PLOS’s NAAS tool - https://ngplosjournals.pagemajik.ai/artanalysis, NAAS will process the files provided and display the results in the "Uploaded Files" section of the page as the processing is complete. If the uploaded figures meet our requirements (or NAAS is able to fix the files to meet our requirements), the figure will be marked as "fixed" above. If NAAS is unable to fix the files, a red "failed" label will appear above. When NAAS has confirmed that the figure files meet our requirements, please download the file via the download option, and include these NAAS processed figure files when submitting your revised manuscript.

**Reproducibility:**

To enhance the reproducibility of your results, we recommend that authors of applicable studies deposit laboratory protocols in protocols.io, where a protocol can be assigned its own identifier (DOI) such that it can be cited independently in the future. Additionally, PLOS ONE offers an option to publish peer-reviewed clinical study protocols. Read more information on sharing protocols at https://plos.org/protocols?utm_medium=editorial-email&utm_source=authorletters&utm_campaign=protocols

Attachment Submitted filename: STARIT comments.docx

Attachment Submitted filename: review_PCOMPBIOLD2600215.docx

# Author comment: Author response to Decision Letter 1

**DOI:** 10.1371/journal.pcbi.1014845.r002

3 Sep 2026

Attachment Submitted filename: 0_Point_by_Point_Response_STARIT_clean.pdf

# Peer review: Decision Letter 1

**DOI:** 10.1371/journal.pcbi.1014845.r003

**Authors:** Ferhat Ay, Virginie Uhlmann

22 Sep 2026

Dear Dr. Fan,

We are pleased to inform you that your manuscript 'Spatial Transcriptomics As Rasterized Image Tensors (STARIT) characterizes cell states with subcellular molecular heterogeneity' has been provisionally accepted for publication in PLOS Computational Biology.

Before your manuscript can be formally accepted you will need to complete some formatting changes, which you will receive in a follow up email. A member of our team will be in touch with a set of requests.

Please note that your manuscript will not be scheduled for publication until you have made the required changes, so a swift response is appreciated.

IMPORTANT: The editorial review process is now complete. PLOS will only permit corrections to spelling, formatting or significant scientific errors from this point onwards. Requests for major changes, or any which affect the scientific understanding of your work, will cause delays to the publication date of your manuscript.

Should you, your institution's press office or the journal office choose to press release your paper, you will automatically be opted out of early publication. We ask that you notify us now if you or your institution is planning to press release the article. All press must be co-ordinated with PLOS.

Thank you again for supporting Open Access publishing; we are looking forward to publishing your work in PLOS Computational Biology.

Best regards,

Virginie Uhlmann

Academic Editor

PLOS Computational Biology

Ferhat Ay

Section Editor

PLOS Computational Biology

***********************************************************

Reviewer's Responses to Questions

**Comments to the Authors:**

**Please note here if the review is uploaded as an attachment.**

Reviewer #1: The authors implemented all the changes.

Reviewer #2: The authors have addressed all my comments in a satisfactory manner. I have no further comments and feel that the manuscript is ready for publication.

Reviewer #3: The authors have addressed all of my concerns

Reviewer #4: The authors have satisfactorily replied to all my comments. Based on the new information, I particularly liked their recommendation of having a hierarchical workflow for larger datasets; by first identifying interesting cells/genes using conventional gene-count clustering and then applying STARIT to such selected cell types/gene subsets.

**********

**Have the authors made all data and (if applicable) computational code underlying the findings in their manuscript fully available?**

The PLOS Data policyrequires authors to make all data and code underlying the findings described in their manuscript fully available without restriction, with rare exception (please refer to the Data Availability Statement in the manuscript PDF file). The data and code should be provided as part of the manuscript or its supporting information, or deposited to a public repository. For example, in addition to summary statistics, the data points behind means, medians and variance measures should be available. If there are restrictions on publicly sharing data or code —e.g. participant privacy or use of data from a third party—those must be specified.

Reviewer #1: Yes

Reviewer #2: Yes

Reviewer #3: None

Reviewer #4: Yes

**********

PLOS authors have the option to publish the peer review history of their article (what does this mean?). If published, this will include your full peer review and any attached files.

If you choose “no”, your identity will remain anonymous but your review may still be made public.

**Do you want your identity to be public for this peer review?** For information about this choice, including consent withdrawal, please see our Privacy Policy.

Reviewer #1: No

Reviewer #2: No

Reviewer #3: **Yes:**  Ashley L Kiemen

Reviewer #4: No

# Editor report: Acceptance letter

**DOI:** 10.1371/journal.pcbi.1014845.r004

**Authors:** Ferhat Ay, Virginie Uhlmann

PCOMPBIOL-D-26-00215R1

Spatial Transcriptomics As Rasterized Image Tensors (STARIT) characterizes cell states with subcellular molecular heterogeneity

Dear Dr Fan,

I am pleased to inform you that your manuscript has been formally accepted for publication in PLOS Computational Biology. Your manuscript is now with our production department and you will be notified of the publication date in due course.

The corresponding author will soon be receiving a typeset proof for review, to ensure errors have not been introduced during production. Please review the PDF proof of your manuscript carefully, as this is the last chance to correct any errors. Please note that major changes, or those which affect the scientific understanding of the work, will likely cause delays to the publication date of your manuscript.

Soon after your final files are uploaded, unless you have opted out, the early version of your manuscript will be published online. The date of the early version will be your article's publication date. The final article will be published to the same URL, and all versions of the paper will be accessible to readers.

For Research, Software, and Methods articles, you will receive an invoice from PLOS for your publication fee after your manuscript has reached the completed accept phase. If you receive an email requesting payment before acceptance or for any other service, this may be a phishing scheme. Learn how to identify phishing emails and protect your accounts at https://explore.plos.org/phishing.

Thank you again for supporting PLOS Computational Biology and open-access publishing. We are looking forward to publishing your work!

With kind regards,

Sharmila Kamatchi

PLOS Computational Biology | Carlyle House, Carlyle Road, Cambridge CB4 3DN | United Kingdom ploscompbiol@plos.org | Phone +44 (0) 1223-442824 | ploscompbiol.org | @PLOSCompBiol
